# Amplification of *TLO* Mediator Subunit Genes Facilitate Filamentous Growth in *Candida Spp*.

**DOI:** 10.1371/journal.pgen.1006373

**Published:** 2016-10-14

**Authors:** Zhongle Liu, Gary P. Moran, Derek J. Sullivan, Donna M. MacCallum, Lawrence C. Myers

**Affiliations:** 1 Department of Biochemistry, Geisel School of Medicine at Dartmouth, Hanover, New Hampshire, United States of America; 2 Microbiology Research Unit, Division of Oral Biosciences, Dublin Dental University Hospital, University of Dublin, Dublin, Ireland; 3 Aberdeen Fungal Group, School of Medical Sciences, Institute of Medical Sciences, University of Aberdeen, Foresterhill, Aberdeen, United Kingdom; 4 Department of Medical Education, Geisel School of Medicine at Dartmouth, Hanover, New Hampshire, United States of America; University College Dublin, IRELAND

## Abstract

Filamentous growth is a hallmark of *C*. *albicans* pathogenicity compared to less-virulent ascomycetes. A multitude of transcription factors regulate filamentous growth in response to specific environmental cues. Our work, however, suggests the evolutionary history of *C*. *albicans* that resulted in its filamentous growth plasticity may be tied to a change in the general transcription machinery rather than transcription factors and their specific targets. A key genomic difference between *C*. *albicans* and its less-virulent relatives, including its closest relative *C*. *dubliniensis*, is the unique expansion of the *TLO* (TeLOmere-associated) gene family in *C*. *albicans*. Individual Tlo proteins are fungal-specific subunits of Mediator, a large multi-subunit eukaryotic transcriptional co-activator complex. This amplification results in a large pool of ‘free,’ non-Mediator associated, Tlo protein present in *C*. *albicans*, but not in *C*. *dubliniensis* or other ascomycetes with attenuated virulence. We show that engineering a large ‘free’ pool of the *C*. *dubliniensis* Tlo2 (CdTlo2) protein in *C*. *dubliniensis*, through overexpression, results in a number of filamentation phenotypes typically associated only with *C*. *albicans*. The amplitude of these phenotypes is proportional to the amount of overexpressed CdTlo2 protein. Overexpression of other C. *dubliniensis* and *C*. *albicans* Tlo proteins do result in these phenotypes. Tlo proteins and their orthologs contain a Mediator interaction domain, and a potent transcriptional activation domain. Nuclear localization of the CdTlo2 activation domain, facilitated naturally by the Tlo Mediator binding domain or artificially through an appended nuclear localization signal, is sufficient for the CdTlo2 overexpression phenotypes. A *C*. *albicans med3* null mutant causes multiple defects including the inability to localize Tlo proteins to the nucleus and reduced virulence in a murine systemic infection model. Our data supports a model in which the activation domain of ‘free’ Tlo protein competes with DNA bound transcription factors for targets that regulate key aspects of *C*. *albicans* cell physiology.

## Author Summary

The ascomycete fungus *Candida albicans* is a leading cause of hospital-acquired bloodstream infections in the United States. Due to limited anti-fungal drug options, there is an approximately 40% mortality rate and over 10,000 deaths per year associated with systemic *C*. *albicans* infections. It is unknown why *C*. *albicans* is the primary cause of systemic *Candidiasis*, versus related ascomycetes such as *Candida dubliniensis*. The genomes of *C*. *albicans* and *C*. *dubliniensis* are remarkably similar, yet *C*. *dubliniensis* has reduced virulence and exhibits less phenotypic plasticity. A striking genomic difference between the fungi is the amplification of the *TLO* (TeLOmere-associated) genes in *C*. *albicans*, which encode a fungal-specific subunit of the Mediator co-activator complex. Amplification results in a large pool of ‘free’ (non-Mediator associated) Tlo protein in *C*. *albicans* that is absent in *C*. *dubliniensis*. Engineering a large ‘free’ pool of Tlo protein in *C*. *dubliniensis*, through overexpression, results in phenotypes common in *C*. *albicans*, yet typically absent in *C*. *dubliniensis*. Tlo proteins contain a potent transcriptional activation domain. Nuclear localization of the Tlo activation domain is necessary and sufficient for the *TLO* overexpression phenotypes. This study provides a mechanistic explanation for how *TLO* amplification in *C*. *albicans* may enhance its virulence.

## Introduction

It is estimated that there are between 2 to 5 million species of fungi on earth, of which only a small fraction cause infections in humans [1]. An even smaller fraction of these are capable of causing life-threatening infections. Nonetheless, opportunistic fungal infections have emerged as a major cause of human disease [2,3,4]**.** *Cryptococcus*, *Candida*, *Aspergillus*, and *Pneumocystis* species account for more than 90% of fungal-related deaths [2]. *Candida albicans* is the primary agent of invasive *candidiasis* [3]. *C*. *albicans* can switch from growth as a natural human commensal to a pathogen and, particularly if the host is immuno-compromised, cause life-threatening systemic infections that have limited treatment options [5,6,7,8,9,10]. It is not well understood, from an evolutionary standpoint, why *C*. *albicans* is more potent pathogen in humans than closely related fungi that have attenuated virulence. The genomes of the *ascomycetes C*. *albicans* and *Candida dubliniensis* are remarkably similar, with 96.3% of genes exhibiting >80% identity and 98% of genes being syntenic. This close phylogenetic relationship is contrasted by the observation that *C*. *dubliniensis* is far less pathogenic in a range of infection models and is a far less prevalent cause of systemic infections [11]. Consistent with this observation, the ability to change morphology or adapt to stress in response to environmental cues, which is critical to *C*. *albicans* virulence, is compromised in *C*. *dubliniensis* [11].

The adaptive transitions that underlie *C*. *albicans* virulence are driven by diverse transcriptional programs, which require the coordination of multiple sequence-specific DNA-bound transcription factors. There are two primary morphological transitions, relevant to virulence, which are interrelated and regulated in response to a range of conditions. First, *C*. *albicans* transitions between yeast and filamentous (pseudohyphal and hyphal) forms—a trait that is strongly associated with virulence [10]. Second, *C*. *albicans* switches from planktonic growth to the formation of highly recalcitrant surface-associated biofilms. Biofilm formation requires the ability to switch between yeast and filamentous growth, and the induction of other pathways involved in matrix production and drug resistance [12,13]. *C*. *albicans* is one of the fungi most commonly isolated from catheter-based biofilms [14,15,16,17]. In addition to morphological transitions, there are multiple coordinated responses to stress that help *C*. *albicans* adapt to host niches and cope with the immune response [18]. In addition to impacting virulence, *C*. *albicans*’ transcriptional plasticity also impacts its commensal lifestyle [19], which has most likely exerted significant selective pressure on its evolution. The origin of *C*. *albicans*’ morphological and transcriptional plasticity isn’t clear, but comparative biochemical and genetic studies utilizing *C*. *albicans* and *C*. *dubliniensis* may be able to shed light on this question.

Considerable effort has been spent on identifying *C*. *albicans* genes critical for morphological transitions [10,20], adhesion/biofilm formation [21,13] and stress responses [18], as well as characterizing the array of individual transcription factors that positively and negatively regulate these genes. Most of these transcription factors are important virulence factors [10,13,18], yet these transcription factors are conserved between *C*. *albicans*, and *C*. *dubliniensis*, as well as many other related fungi. Therefore, what property of the *C*. *albicans* regulatory machinery enables the transcriptional plasticity that underlies its virulence? The answer to this question is most likely multi-fold and may include the particular wiring of gene expression networks [20] and the functional properties of the genes (adhesins, *etc*.) regulated in *C*. *albicans* [22]. Rather than a particular gene or network, however, we hypothesize that amplification of a single component of the *C*. *albicans* general transcription machinery, the *TLO* (*MED2*) subunit of the Mediator complex, can affect transcriptional plasticity.

Mediator, a conserved eukaryotic ~25 subunit co-activator complex, is an intermediary between DNA-bound transcription factors and the general transcription machinery in all eukaryotes [23,24]. Based on work in *C*. *albicans* [25–27], *C*. *dubliniensis* [28], and *S*. *cerevisiae*, it is clear that some subunits modulate the transcription of specific subsets of genes [29,30], while others facilitate the transcription of virtually all genes [31]. About half of fungal Mediator subunits are encoded by essential genes, which typically have amino acid identity levels as great as 20–30% with their mammalian counterparts. The Tlo (named Med2 in the formal Mediator nomenclature) and Med3 subunits, however, are widely divergent among *Ascomycetes*, and have no metazoan orthologs that combine similar sequence, structure and function. Mediator subunits Tlo/Med2, Med3 and Med15 are encoded by non-essential genes and are held in the Tail module through mutual interactions [32,33,26,28]. Typically the protein subunits of Mediator, and the genes encoding them are present in a one to one ratio [34]. However, there are as many as 15 *TLO* paralogs encoded by the *C*. *albicans* genome, compared to two in *C*. *dubliniensis* and one in all other sequenced fungi [35,36].

Sequence analysis of the *C*. *albicans TLO*s (*CaTLO*s) divided the paralogs into α, β and γ clades [37], of which the α and β clades were expressed at vastly higher mRNA [37] and protein levels [26] than γ. The *C*. *albicans* strain used in our studies has one β clade Tlo and a roughly equally number of α and γ clade paralogs [37]. The two *C*. *dubliniensis TLO* (*CdTLO*) paralogs, *CdTLO1* and *CdTLO2*, diverge from the *C*. *albicans* clades, and each other, primarily in their C-termini [28]. *CdTLO1* is the primary *TLO* in *C*. *dubliniensis*, and is expressed at least 30X higher at the protein level [28] and ~50x higher at the mRNA level [35] than *CdTLO2*. The CaTlo α and β proteins are incorporated, with comparable affinity, into Mediator. Hence, there are several different pools of Mediator complex in *C*. *albicans*, each with a different Tlo protein [26]. Since each of the CaTlo α and β proteins are expressed in excess of other CaMediator subunits, there is also a large pool of ‘free’ CaTlo protein that is nuclear localized [37], not associated with Mediator [26]. There is no published data to support the idea that the ‘free’ Tlo protein is a stoichiometric member of an alternative complex. In contrast, just enough CdTlo1 is expressed in *C*. *dubliniensis* for it to be a stoichiometric component of CdMediator, leaving no detectable ‘free’ Tlo pool [28]. Null mutants of *C*. *albicans*, or *C*. *dubliniensis*, *MED3*, and *C*. *dubliniensis TLO1/2* exhibit several phenotypes including decreased resistance to oxidative stress, inability to utilize galactose as a carbon source, and varied cellular and colony morphology phenotypes [26–28,35]. The highly acidic C-termini of the CaTlo α and β proteins, and the C-termini of the CdTlo proteins encode transcriptional activation domains (TADs) [38]. Our one-hybrid experiments showed that the Tlo C-termini were potent TADs that functioned independently of incorporation into the Mediator complex, a property encoded exclusively by the Tlo N-terminus [38]. Even though the sequence of CaTlo and CdTlo C-termini diverge substantially from the *S*. *cerevisiae* ortholog (Med2), the potent TAD is also present in the model yeast [38]. Removing these C-terminal TADs in *C*. *dubliniensis* and *S*. *cerevisiae* left a Mediator complex that was deficient in its transcriptional response to alternative carbon sources and certain stresses [38]. We hypothesized that the ‘free’ Tlo population impacts morphological plasticity in *C*. *albicans* through a mechanism that is dependent on its TAD.

Here we show that engineering a large ‘free’ pool of CdTlo2 protein in *C*. *dubliniensis*, through overexpression, results in a number of virulence associated filamentation phenotypes that are primarily associated with *C*. *albicans*. These phenotypes are specific to *CdTLO*2, versus other *CaTLO*s and *CdTLO1*, and their amplitude is proportional to the amount of overexpressed Tlo protein. Nuclear localization of the CdTlo2 TAD, facilitated naturally by the Mediator association domain or artificially through a nuclear localization signal, is sufficient for the *C*. *dubliniensis* Tlo overexpression phenotypes. Mutations that inhibit the nuclear localization of Tlo protein in *C*. *albicans* strongly reduce its virulence in a murine systemic infection model. This data suggests that the ‘free’ pool of Tlo protein may be competing with DNA-bound activators for binding sites on co-activators and co-repressors.

## Results

### Overexpression of *CdTLO1* in *C*. *dubliniensis* does not result in an accumulation of free Tlo protein

**Fig 1:**
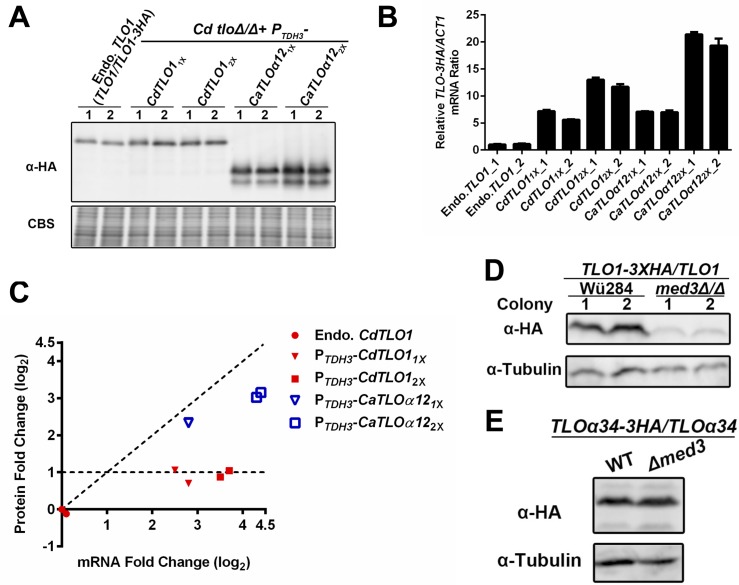
CdTlo1 and CaTlos differ in their ability to be stably expressed in a non-Mediator associated form. **(A)** Immunoblot shows that over-expression of one (1X) and two (2x) copies of HA-tagged *CaTLOα12* (from strains yLM303 and yLM305), but not C*dTLO1* (from strains yLM302 and yLM304), from the *TDH3* promoter results in increased protein levels, compared to a HA-tagged endogenous copy of *CdTLO1* (yLM301) in *C*. *dubliniensis*. Two independent transformants (‘1’ and ‘2’) were tested. Coomassie blue staining (CBS) was used as a loading control. **(B)** RT-qPCR analysis shows that over-expression of one (1X) and two (2x) copies of HA-tagged *CaTLOα12* (from strains yLM303 and yLM305) and C*dTLO1* (from strains yLM302 and yLM304) from the *TDH3* promoter both result in increased mRNA levels, compared to an endogenous copy of *CdTLO1* (yLM301) in *C*. *dubliniensis*. The error bars represent the technical deviation of two sets of qPCR measurements on a given sample. Two independent transformants (‘1’ and ‘2’) were tested. **(C)** Plot of fold-changes of *CdTLO1* and *CaTLOα12* protein versus mRNA (from Fig 1A and 1B) reveals that CdTlo1 protein levels plateau compared to CaTloα12p. The dashed lines represent an idealized slope of 1 in which the fold-change in protein is equal to the fold-change in mRNA, and an idealized slope of zero in which there is no increase in protein with increasing mRNA. **(D)** Immunoblot shows that endogenous HA-tagged CdTlo1 protein level drops in *med3Δ/Δ* (yLM308) versus a wild type (yLM301) *C*. *dubliniensis* strain. Two independent transformants (‘1’ and ‘2’) were tested. An anti-tubulin antibody was used as a loading control. **(E)** Immunoblot shows endogenous protein levels of HA-tagged Tloα34 (yLM391) are not affected upon *med3* deletion (yLM392) in *C*. *albicans*. An anti-tubulin antibody was used as a loading control.

**Fig 2:**
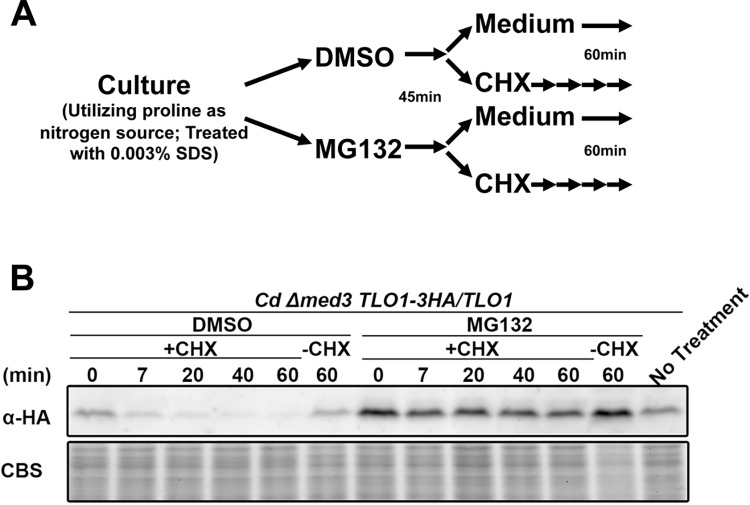
Non-Mediator associated CdTlo1p is rapidly degraded by the proteasome. **(A)** Schematic of treatment cells with cycloheximide (CHX) and the MG132 proteasome inhibitor to determine whether a Tlo protein is subject to proteasome dependent degradation. **(B)** Immunoblot of endogenous HA-tagged CdTlo1p in a wild type a *med3Δ/Δ C*. *dubliniensis* strain (yLM308) after treatment with cycloheximide, in the absence and presence of the proteasome inhibitor MG132. Coomassie blue staining (CBS) was used as a loading control.

Two primary approaches were available to investigate the impact of a ‘free’, non-Mediator associated Tlo pool on virulence related phenotypes. The first approach, depletion of the ‘free’ Tlo pool in *C*. *albicans*, is the most challenging technically. Knocking out both copies of all, or any, of the 14 *CaTLO* paralogs using conventional gene replacement methods has proven to be difficult given the high rates of recombination between *TLO*s at these loci [39]. The second approach, which we have successfully utilized here, was to create a ‘free’ Tlo population in *C*. *dubliniensis*, where it doesn’t normally exist. CdTlo1 and *C*. *albicans* α-clade CaTlo proteins, the predominantly expressed Tlos in their respective organisms, were initially chosen for overexpression in *C*. *dubliniensis*. The strong *TDH3* promoter (*P*_*TDH3*_) was used to give high levels of transcription [40] of the *TLOs* in *C*. *dubliniensis*. Overexpressed proteins were HA tagged on the C-terminus and integrated into the native *CdTLO1* locus (S1 Fig) unless otherwise stated. Immunoblotting revealed that integration of either one or two copies of the *CdTLO1* gene under the control of the *P*_*TDH3*_ promoter yielded only modest increases in CdTlo1p protein versus endogenous HA-tagged CdTlo1p, while integration of increasing copies of *CaTLOα12* led to increasing amounts of the protein that were in excess of the endogenous CdTlo1p (Fig 1A). The lack of increase in CdTlo1p was not related to mRNA levels, as RT-qPCR revealed that the constructs led to substantial increases in mRNA as expected (Fig 1B). Plotting the ratio of fold change in protein versus fold change in mRNA (Fig 1C) showed that the increased amount of CdTlo1 protein plateaued at a level ~2-fold higher than endogenous, while CaTloα12 protein continued to increase with increasing mRNA levels. Co-overexpression of *CdTLO1* and *CaTLOα12* led to a decrease in CdTlo1p (S2 Fig) that suggested CaTloα12p was competing for a factor that stabilized CdTlo1p. Our previous work had shown that the α-clade CaTlo proteins were stable in a non-Mediator associated form in *C*. *albicans* [26]. We speculated that CdTlo1 protein might only be stable when associated with Mediator complex, and that CdTlo1 protein in excess of that amount was rapidly degraded. This idea was supported by the finding that deletion of *MED3*, which releases the Tlo subunit from the Tail module of Mediator [26,28], led to a large decrease of endogenous CdTlo1p in *C*. *dubliniensis* (Fig 1D) and no decrease in an endogenous α-clade *TLO*, CaTloα34p (Fig 1E), in *C*. *albicans*. RT-qPCR showed that the decreased CdTlo1p levels in the *med3* null strain are not a result of decreased mRNA amounts (S3 Fig). The compromised stability of ‘free’ CdTlo1p is intrinsic to its sequence, rather than species specific, as overexpression of *CdTLO1* under the *ACT1* promoter (*P*_*ACT1*_) in *C*. *albicans* shows the same low protein to mRNA ratio (S4 Fig) observed in *C*. *dubliniensis* (Fig 1). Treatment with cycloheximide led to the rapid degradation of non-Mediator associated CdTlo1p in *C*. *dubliniensis*, created by deleting *MED3* in an endogenous *TLO1-HA* tagged strain, while ‘free’ CaTlo protein was stable in *C*. *albicans* (Fig 2 and S5 Fig). A similar difference in stability was observed when HA-tagged *CdTLO1* and *CaTLOα12* were overexpressed in *C*. *albicans* (S6 Fig). Treating the *C*. *dubliniensis* endogenous *CdTLO1-HA med3* null strain with a proteasome inhibitor (MG132) prior to treatment with cycloheximide prevented the degradation of non-Mediator associated CdTlo1 protein (Fig 2). MG132 had a similar effect on the degradation of overexpressed CdTlo1-HA in *C*. *albicans* (S7 Fig). We conclude from this set of experiments that there is an intrinsic property of the CdTlo1 sequence that makes the non-Mediator associated protein subject to rapid proteasome-dependent degradation, hence preventing the accumulation of a large ‘free’ pool of the protein. This result was consistent with the inability of *CdTLO1* overexpression in *C*. *dubliniensis* to confer any notable phenotypes (S1 Table).

### Substitution of two N-terminal stretches from CaTlos confer greater stability to the non-Mediator associated form of the CdTlo1 protein

**Fig 3:**
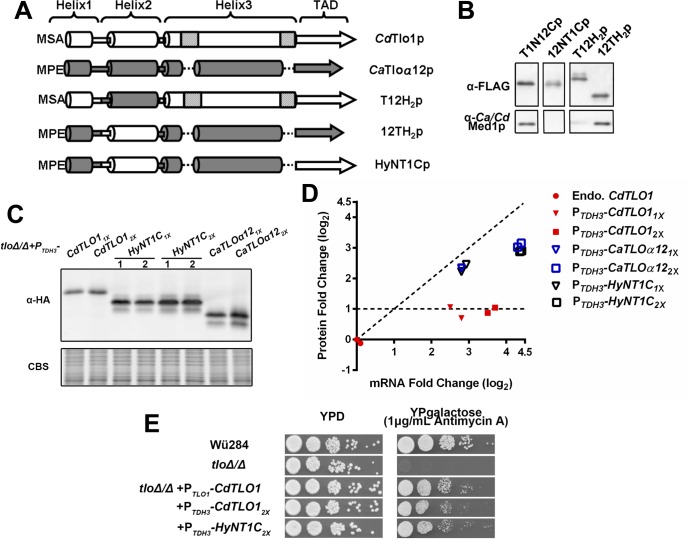
Replacement of ‘Helix 1’ and ‘Helix 3’ of CdTlo1p with the ‘Helix 1’ and ‘Helix 3’ of CaTloα12p creates a stable hybrid protein that is readily incorporated into purified *C*. *dubliniensis* Mediator and complements a *C*. *dubliniensis* tlo null phenotype. **(A)** Schematic of CdTlo1/CaTloα12 hybrid proteins. Specific amino acid sequences for CdTlo1 are: Helix 1 (1–38), Helix 2 (39–74), Helix 3 (75–198) and TAD (199–320). Specific amino acid sequences for CaTloα12 are: Helix 1 (1–38), Helix 2 (39–74), Helix 3 (75–165) and TAD (166–252). In each panel the first three amino-acids (MSA or MPE) are denoted at the beginning of each construct. The segments shaded light grey represent regions of CdTlo1p ‘Helix 3’ that do not have clear homologous sequence in CaTloα12 ‘Helix 3.’ **(B)** Immunoblot analysis of affinity purified 6His-3Flag tagged T1N12Cp (CdTlo1 (1–198))/CaTloα12 (166–252); from yLM315), 12NT1Cp (CaTloα12 (1–165))/CdTlo1 (199–320);from yLM321), T12H_2_p (CdTlo1(1–38)/CaTloα12(39–74)/CdTlo1(75–320);from yLM325) and 12TH_2_p(CaTloα12(1–38)/CdTlo1(39–74)/CaTloα12(75–252); from yLM326). An anti-Med1 antibody is used to show the ability of the 6His-3Flag tagged hybrid protein to pull down intact *C*. *dubliniensis* Mediator. **(C)** Immunoblot showing over-expression of one (1X) and two (2x) copies of HA-tagged C*dTLO1* (yLM302 and yLM304 respectively), *HyNT1C* (CaTloα12(1–38)/CdTlo1(39–74)/CaTloα12(75–165)/CdTlo1(199–320); yLM328 and yLM329 respectively) and *CaTLOα12* (yLM303 and yLM305 respectively), from the *TDH3* promoter in a *C*. *dubliniensis tloΔ/Δ* strain. Two independent transformants (‘1’ and ‘2’) were tested. Coomassie blue staining (CBS) was used as a loading control. **(D)** Plotting of fold-change of *HyNT1C* mRNA and protein into Fig 1C reveals that the hybrid protein has stability similar to CaTloα12p. The dashed lines represent an idealized slope of 1 in which the fold-change in protein is equal to the fold-change in mRNA, and an idealized slope of zero in which there is no increase in protein with increasing mRNA. **(E)** When over-expressed in a *C*. *dubliniensis tloΔ/Δ* strain, *HyNT1C* complements *gal-* phenotype of the null mutant with comparable efficiency to native or overexpressed *CdTLO1*.

Despite its relative stability in the ‘free’ form, overexpression of *CaTLOα12* in *C*. *dubliniensis* also did not confer any notable phenotypes (S1 Table). We speculated that divergent sequences in the CaTlo proteins, compared to CdTlo1, might prevent them from impacting phenotypes in *C*. *dubliniensis*. Since we were unable to isolate fully intact Mediator complex from a *Cdtlo* null strain that expressed *CaTLOα12* (S8 Fig), we speculated that inefficient Mediator interactions could prevent CaTloα12p from impacting phenotype in *C*. *dubliniensis*. Hence, to engineer a version of CdTlo1p that could be stably expressed in a ‘free’ form, and to gain insight into native CdTlo1p instability relative to CaTloα12p, we engineered a hybrid overexpression construct that combined sequences from *CaTLOα12* and *CdTLO1*. To determine what C-terminal sequence of CaTloα12p was critical for its differing stability from CdTlo1 in the non-Mediator associated form, HA-tagged overexpression constructs were made in which increasing lengths of CaTloα12p C-terminal sequence was replaced by the corresponding C-terminal sequence from CdTlo1p (S9 Fig). Evaluating these hybrid proteins by comparing protein to mRNA ratios showed that ‘unstructured’ C-terminal CaTloα12p TAD was not required, but the predicted ‘Helix 3’ (PSIPRED[41]) in the N-terminus of CaTloα12p was required for its relative stability in the ‘free’ form in *C*. *dubliniensis* (S10 Fig) and *C*. *albicans* (S11 Fig). A similar approach with N-terminal sequences revealed that the predicted ‘Helix 1’ of CaTloα12 also was important to its relative stability when overexpressed in *C*. *albicans* (S12 and S13 Figs). Purifying the N- and C-terminal hybrid Tlo proteins with a 6His-3Flag tag from a *C*. *dubliniensis* overexpression strain showed that retention of predicted ‘Helix 2’ from CdTlo1 was necessary to ensure incorporation into Mediator (S14 Fig). Using this information, a hybrid protein, HyNT1Cp (Hybrid N (HyN)-CdTlo1 C-terminus (T1C)), was created that had the CdTlo1 TAD, was more stable in its ‘free’ form and, by virtue of the CdTlo1 ‘Helix 2,’ was able to be readily incorporated into *C*. *dubliniensis* Mediator (Fig 3). The N-terminus of HyNT1Cp is identical to the N-terminus of 12TH_2_p, which is stably incorporated into CdMediator (Fig 3A and 3B). The N-terminus of the Tlo proteins contains all the Mediator association properties of the Tlo proteins [38]. Increasing copy numbers of the HyNT1Cp overexpression cassette leads to increases in mRNA and protein equivalent to CaTloα12 (Fig 3C and 3D). These properties were further confirmed by demonstrating that co-overexpression of HyNT1Cp and CdTlo1 in *C*. *dubliniensis* led to decreased amounts of CdTlo1p (S15 Fig). HyNT1Cp was also able to complement the *tlo* null *gal-* phenotype [28] in *C*. *dubliniensis* (Fig 3E). Despite these ‘optimized’ properties, overexpression of HyNT1Cp did not confer any notable phenotypes in *C*. *dubliniensis* (S1 Table). Although the other *C*. *dubliniensis TLO* gene, *CdTLO2*, was not typically expressed at high levels in *C*. *dubliniensis*, its sequence diverged from *CdTLO1* and the *CaTLO*s. Hence, we tested whether overexpression of *CdTLO2* might impact *C*. *dubliniensis* phenotype.

### Overexpression of *CdTLO2* in *C*. *dubliniensis* creates a large ‘free’ pool of CdTlo2 protein and promotes filamentous growth

**Fig 4:**
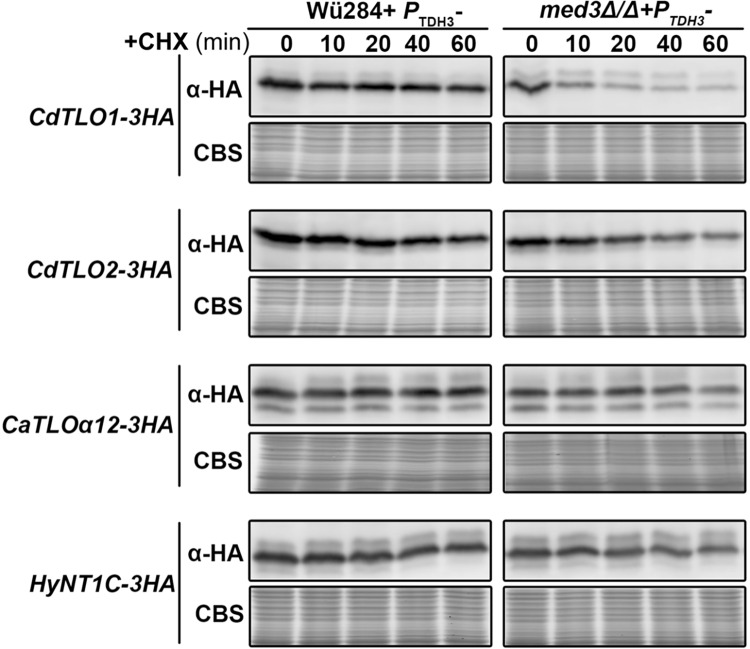
‘Free’ CdTlo2 protein has enhanced stability compared to CdTlo1 in *C*. *dubliniensis* after treatment with cycloheximide (CHX). Immunoblot of overexpressed HA-tagged CdTlo1p, CdTlo2p, CaTloα12p and HyNT1Cp in a wild type Wü284 *C*. *dubliniensis* strain (yLM337, yLM339, yLM335 and yLM341 respectively) and *med3Δ/Δ C*. *dubliniensis* strain (yLM338, yLM340, yLM336 and yLM342 respectively) after treatment with cycloheximide over a time course (minutes). Coomassie blue staining (CBS) was used as a loading control.

**Fig 5:**
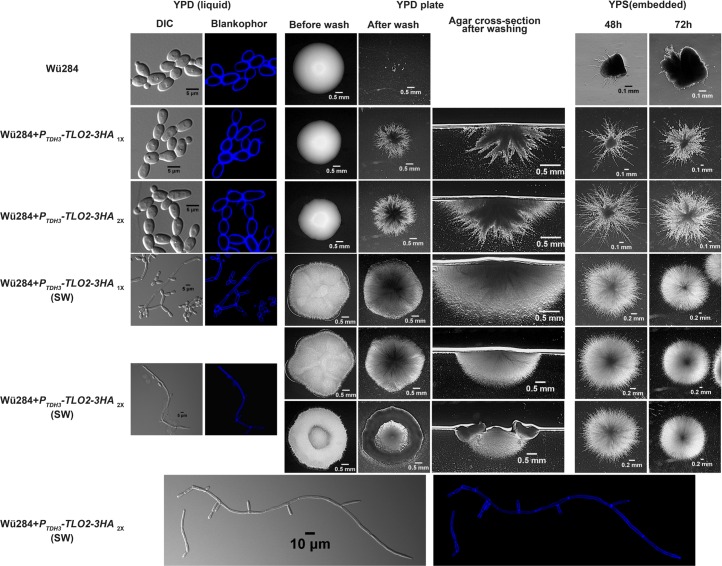
Over-expression of *CdTLO2-3HA* in *C*. *dubliniensis* leads to increased agar invasion and ‘embedded agar’ filamentation. Phenotypic analysis of one (1X) and two (2x) copies of HA-tagged *CdTLO2* expressed from the *TDH3* promoter in a wild type Wü284 *C*. *dubliniensis* strain (yLM339 and yLM344 respectively). A fraction of *CdTLO2-3HA*_*1X*_ and *CdTLO2-3HA*_*2X*_ overexpression construct transformants resulted in colonies with wrinkled surfaces, instead of smooth. These super wrinkled (SW) transformants (yLM343 and yLM345, derived from *CdTLO2-3HA*_*1X*_ and *CdTLO2-3HA*_*2X*_ transformation respectively) were also analyzed. Cells grown in YPD liquid media were analyzed by differential contrast (DIC) microscopy (left column, and bottom left panel) and fluorescent microscopy with the cell wall stained with Blankophor (second column from left, and bottom right panel). Agar invasion is analyzed by growing colonies on YPD/2% agar plates, washing the plates with running water, cutting a cross section through the residual colony and inspecting the cross-section on a stereoscope. Embedded agar filamentation is analyzed by diluting cells in YPS agar (2%) and inspecting the ‘colony’ growth in the agar after 48 and 72 hours using a stereoscope. At least two distinct colony morphologies, which are stably inherited, for SW colonies (*CdTLO2-3HA*_*2X*_ (SW) upper row and lower row) are observed.

**Fig 6:**
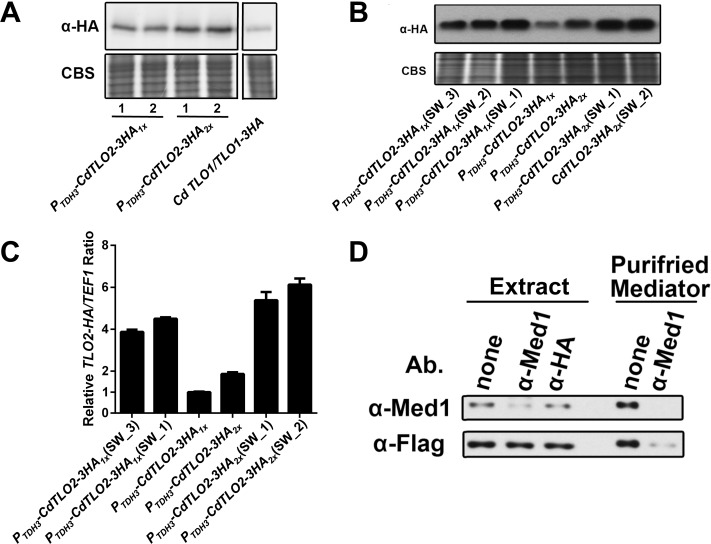
Overexpression of *CdTLO2* in smooth and ‘SW’ cells show increasing amounts of protein and mRNA. **(A)** Immunoblot showing protein overexpression of *CdTLO2-3HA*_*1X*_ (yLM339) and *CdTLO2-3HA*_*2X*_ (yLM344) in *C*. *dubliniensis* compared to endogenous expression of *CdTLO1* (yLM301). Coomassie blue staining (CBS) was used as a loading control. The panels are from different lanes of the same blot/gel. **(B)** Immunoblot showing that multiple isolates of spontaneous SW cells derived from *CdTLO2-3HA*_*1X*_ (yLM343) and *CdTLO2-3HA*_*2X*_ (yLM345) over-expression cassette transformation have higher protein levels than their smooth *CdTLO2-3HA*_*1X*_ (yLM339) and *CdTLO2-3HA*_*2X*_ (yLM344) over-expression counterparts in *C*. *dubliniensis*. Numbering of individual isolates corresponds to numbering in S16 Fig. Coomassie blue staining (CBS) was used as a loading control. **(C)** RT-qPCR analysis showing that multiple isolates of spontaneous SW cells derived from *CdTLO2-3HA*_*1X*_ (yLM343) and *CdTLO2-3HA*_*2X*_ (yLM345) over-expression cassette transformation have higher mRNA levels than their smooth *CdTLO2-3HA*_*1X*_ (yLM339) and *CdTLO2-3HA*_*2X*_ (yLM344) over-expression counterparts in *C*. *dubliniensis*. Numbering of individual isolates corresponds to numbering in above. *CdTLO2* mRNA levels are normalized against *TEF1* mRNA. The error bars represent the technical deviation of two sets of qPCR measurements on a given sample. **(D)** Immunoblot showing immunodepletion of Tlo and Med1 from a crude extract and purified CdMediator sample. A crude sample from a strain in which CdTlo2-6His-3Flag was overexpressed, and a purified intact Mediator sample from a CdTlo1-6His-3Flag strain were used for pull-down experiments. An anti-Med1 antibody was used to deplete Med1 from the two samples. Immunodepletion using anti-HA antibody was used a control. In the purified Mediator sample the CdTlo1-6His-3Flag protein was depleted to the same degree as the Med1p, while in the extract the overexpressed CdTlo2-6His-3Flag protein was largely unaffected by the anti-Med1 depletion.

Overexpression of CdTlo2 results in a stability profile that is increased compared to CdTlo1, but slightly less stable than CaTlos and HyNT1Cp (Fig 4). Most importantly, it shows that the typically weakly expressed CdTlo can accumulate in the ‘free’ form (Fig 4). Unlike what we had observed with *CdTLO1* or *CaTLOs*, over expression of the 1X and 2X *CdTLO2* constructs (S1 Fig) in an otherwise wild type *C*. *dubliniensis* background led to dramatic increases in filamentous growth in embedded agar, and agar invasion from colonies (Fig 5). These 1X and 2X *CdTLO2* strains, when grown in liquid YPD media, do not exhibit any apparent morphological differences from the wild type *C*. *dubliniensis* strain (Fig 5). When PCR positive transformants were isolated from the integration of the 1X and 2X *CdTLO2* OE constructs, two colony morphologies were observed–‘smooth’ and ‘super wrinkled’ (SW) (Fig 5). These SW colonies constituted ~25% of the 1X *CdTLO2* OE and ~50% of the 2X *CdTLO2* OE integration positive transformants. The wrinkled SW colony appearance could vary between transformants (Fig 5). In addition, both 1X and 2X *CdTLO2* OE SW strains grew in a filamentous form in liquid YPD media, and demonstrated a greater degree of agar invasion and embedded filamentation than their non-SW counterparts (Fig 5). We have never observed conversion of the ‘smooth’ form to the ‘SW’ form once a 1X or 2X *CdTLO2* OE strain adopted the ‘smooth’ morphology. We did, however, infrequently observe that the 1X or 2X *CdTLO2* OE ‘SW’ strains adopt the smooth morphology, an event initially manifested as a sectored colony. qPCR of *CdTLO2* in genomic DNA from 1X and 2X *CdTLO2* OE ‘smooth’ and ‘SW’ colonies showed that there were multiple copies of *CdTLO2* over expression cassettes in the ‘SW’ strain compared to the ‘smooth’ strains (S16 Fig). It appears that 4 total copies of HA-tagged CdTLO2 are sufficient to induce a SW phenotype and that additional copies amplify the phenotype (S16 Fig). Since there is no ploidy increase in Chromosome 7 (on which *CdTLO1* is encoded) relative to other chromosomes in these strains (S17 Fig), we believe that the overexpression construct has been inserted in additional genomic locations as opposed to the amplification of Chromosome 7. The insertion location(s) for these extra *CdTLO2* copies in the ‘SW’ strains is currently unknown, but does not appear to be the second *CdTLO1* locus since we continue to detect *CdTLO1* genomic DNA in these strains. We observed that increases in *CdTLO2* OE copy number led to increased amounts of *CdTLO2* mRNA and protein product, which correlated with an increasing amount of filamentation (Figs 5 and 6). A majority of the overexpressed CdTlo2 protein is in a non-Mediator associated form as shown by the ratio of CdTlo2 to Med1 protein in a cell extract versus the ratio of Tlo to Med1 in a purified Mediator sample (Fig 6D). The existence of this ‘free’ CdTlo2p pool is also supported by the observation that an anti-Med1 antibody is able to pull-down almost all of the Tlo subunit in a purified Mediator sample, but only a small fraction of the overexpressed CdTlo2 protein in an extract (Fig 6D). From these studies, it appears that a *CdTLO2* OE cassette copy number of approximately four is sufficient to induce the super wrinkled phenotype (S16C Fig). The increased filamentation, as well as the emergence of the SW phenotype, were not specific to using the *TDH3* promoter for *CdTLO2* overexpression or to the Wü284 strain background. Using *ACT1* and *ENO1* promoters to overexpress *CdTLO2* in Wü284 and two other *C*. *dubliniensis* strain backgrounds resulted in similar morphologies to the *TDH3* promoter driven overexpression in Wü284 (S18 Fig). The filamentation phenotypes were specific to *CdTLO2* overexpression, as similar overexpression of *CdTLO1*, *CaTLOα12* or the stabilized hybrid construct, *HyNT1C*, did not result in agar invasion or filamentation in embedded agar (S19 Fig). We had previously shown that *C*. *dubliniensis med3* and *tlo1* null strains exhibited growth defects under a number of stress and alternative carbon source conditions [28]. Testing *CdTLO2* OE strains for an effect on these phenotypes using plate-based assays, however, revealed only minor growth differences compared to WT (S20 Fig). Interestingly, colonies from the *CdTLO2* and *CdTLO2 (SW)* overexpression strains grown with galactose as the sole carbon source showed increased filamentation compared to control strains (S20 Fig).

### Both the N-terminal Mediator association domain and C-terminal activation domain are required for *CdTLO2* overexpression driven filamentation in *C*. *dubliniensis*

**Fig 7:**
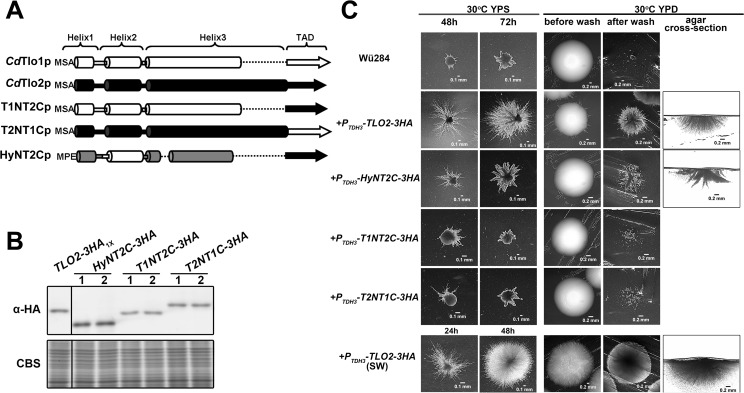
The CdTlo2p C-terminal TAD, but not the CdTlo1p C-terminal TAD fused to a stabilized CdTlo1 N-terminus is able to confer overexpression filamentation phenotypes similar to CdTlo2p. **(A)** Schematic of *CdTLO1*/*CdTLO2* hybrid constructs for overexpression in *C*. *dubliniensis*. Specific amino acid sequences for T1NT2Cp are CdTlo1p (1–198)/CdTlo2p (255–367). Specific amino acid sequences for T2NT1Cp are CdTlo2p (1–254)/CdTlo1p (199–320). The HyNT2Cp construct contains the stabilized CdTlo1/CaTloα12 hybrid N-terminal domain (**Fig 3A**) fused to the CdTlo2p TAD (255–367). The first three amino-acids (MSA or MPE) are denoted at the beginning of each construct. **(B)** Immunoblot showing overexpression of one copy of HA-tagged C*dTLO2*(yLM339), *HyNT2C*(yLM352), *T1NT2C*(yLM350) and *T2NT1C*(yLM351) from the *TDH3* promoter in a wild type *C*. *dubliniensis* strain. Two independent transformants (‘1’ and ‘2’) were tested. Coomassie blue staining (CBS) was used as a loading control. **(C)** Embedded agar filamentation (two left columns) and agar invasion (three right columns) phenotype analysis with one copy of HA-tagged C*dTLO2*(yLM339), *HyNT2C*(yLM352), *T1NT2C*(yLM350) and *T2NT1C*(yLM351) overexpressed from the *CdTDH3* promoter in a wild type (Wü284) *C*. *dubliniensis* strain. The ‘SW’ strain (yLM343) derived from *CdTLO2-3HA*_*1X*_ transformation was also included in the comparison.

**Fig 8:**
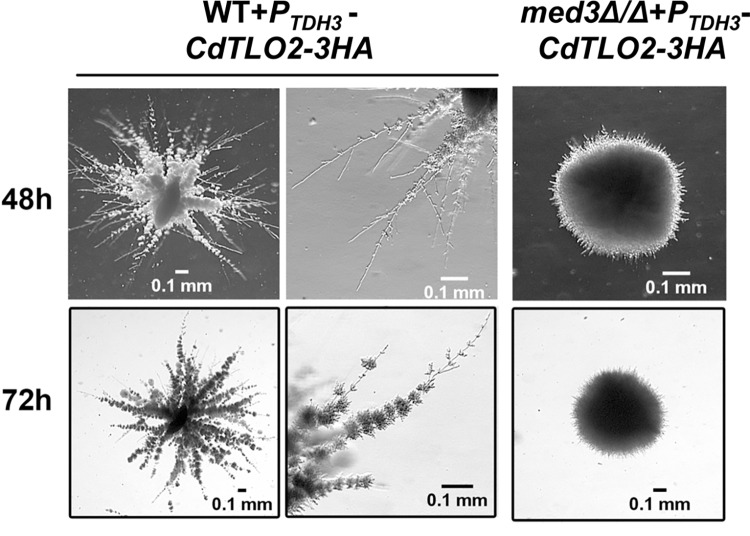
The *CdTLO2* overexpression embedded agar filamentation phenotype observed in wild type *C*. *dubliniensis* is not observed in a *med3Δ/Δ C*. *dubliniensis* strain. Embedded agar filamentation in wild type (yLM339) and *med3Δ/Δ* (yLM340) *C*. *dubliniensis* strains overexpressing *CdTLO2* from a *TDH3* promoter is analyzed by diluting cells in YPS agar and inspecting the ‘colony’ growth in the agar after 48 and 72 hours using a stereoscope.

In addition to the stability of the ‘free’ form of CdTlo2p, the C-terminal CdTlo2p TAD was also required for overexpression phenotypes. Replacing the C-terminal activation domain of CdTlo2p with the CdTlo1p activation domain in our overexpression constructs resulted in the loss of the filamentation phenotype (Fig 7). Fusing the C-terminal *CdTLO2* TAD to the N-terminus of the stabilized CdTlo1 hybrid protein, HyNT2Cp (Hybrid N (HyN)-CdTlo2 C-terminus (T2C)), in an overexpression construct resulted in similar, albeit weaker filamentation phenotypes to the *CdTLO2* overexpression construct, while fusing this same activation domain to the intrinsically unstable *CdTLO1* N-terminal domain did not (Fig 7). We have previously shown that the N-terminus of a Tlo protein was necessary and sufficient for incorporation of the subunit into Mediator through interactions with the Med3 subunit [38]. To test whether association with Mediator was important for the *CdTLO2* overexpression phenotypes, we integrated the *CdTLO2* overexpression construct into a *med3* null *C*. *dubliniensis* background. The phenotypes associated with *CdTLO2* overexpression, such as filamentation in embedded agar (Fig 8), were not observed upon *CdTLO2* overexpression in a *med3* null *C*. *dubliniensis* strain. Further insight into why Med3, and Mediator association, were critical for *CdTLO2* overexpression phenotypes resulted from evaluating the nuclear localization of the Tlo proteins.

### Nuclear localization of the *TLOs* is dependent on the Med3 and Med15 subunits that link Tlo subunit to Mediator, and is correlated with virulence in *C*. *albicans*

**Fig 9:**
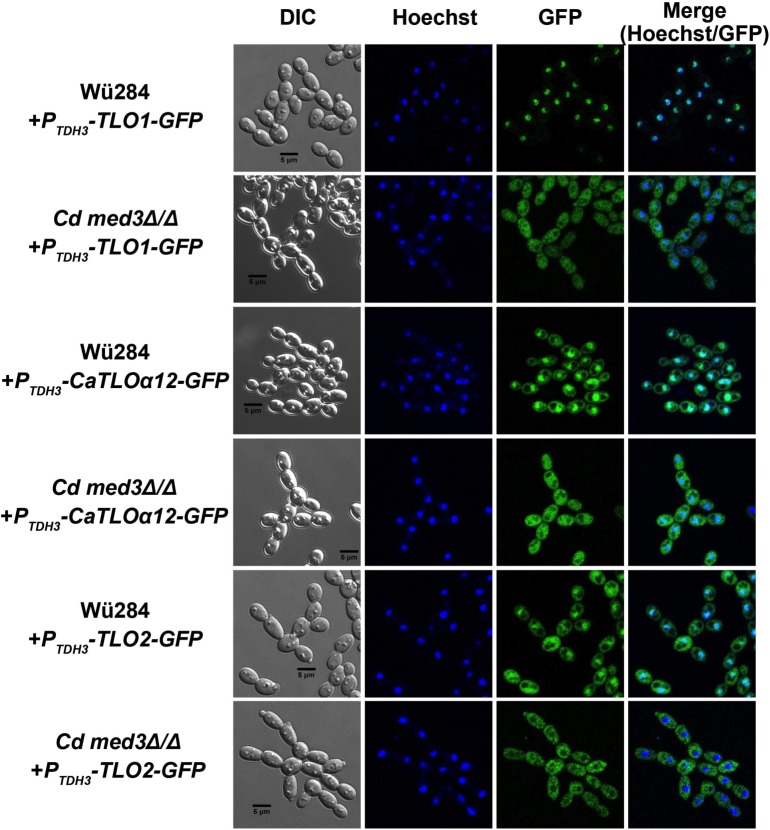
Overexpressed Tlo proteins are localized to the nucleus in *C*. *dubliniensis* through a Med3 dependent mechanism. GFP tagged *CdTLO1*, *CaTLOα12* and *CdTLO2* constructs were overexpressed from a *TDH3* promoter in wild type *C*. *dubliniensis* strain (yLM355, yLM353 and yLM357 respectively) and in *med3Δ/Δ C*. *dubliniensis* strain (yLM356, yLM354 and yLM358 respectively). Differential contrast (DIC) and fluorescence microscopy were used to visualize GFP localization, while Hoechst staining was used to stain the nuclei. All cells were grown in synthetic complete media overnight, diluted into the same media and grown for 5–6 hours before visualization.

**Fig 10:**
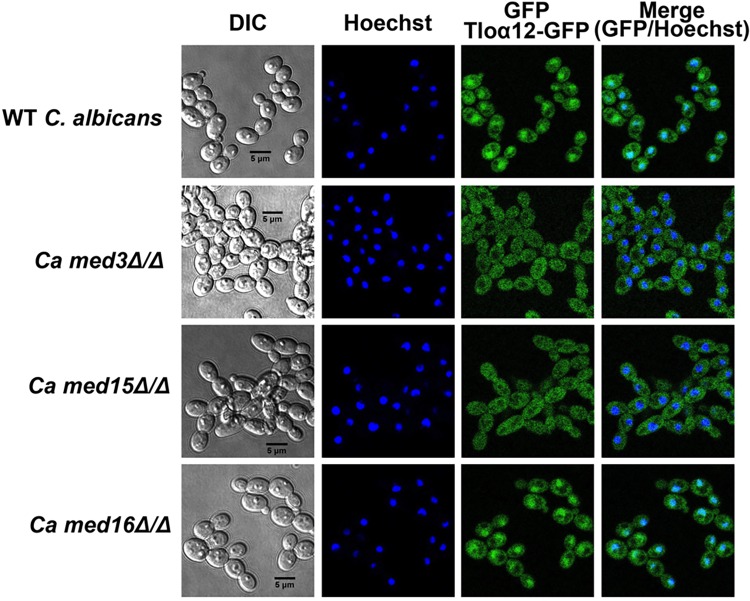
Nuclear localization of overexpressed CaTloα12 protein in *C*. *albicans* is dependent on Med3p and Med15p, but not Med16p. A GFP tagged *CaTLOα12* construct was overexpressed from an *ACT1* promoter in wild type (yLM409), *med3Δ/Δ* (yLM410), *med15Δ/Δ* (yLM411) and *med16Δ/Δ* (yLM412) *C*. *albicans* strains. Differential contrast (DIC) and fluorescence microscopy were used to visualize GFP localization, while Hoechst staining was used to stain the nuclei. All cells were grown in synthetic complete media overnight, diluted into the same media and grown for 5–6 hours before visualization.

**Fig 11:**
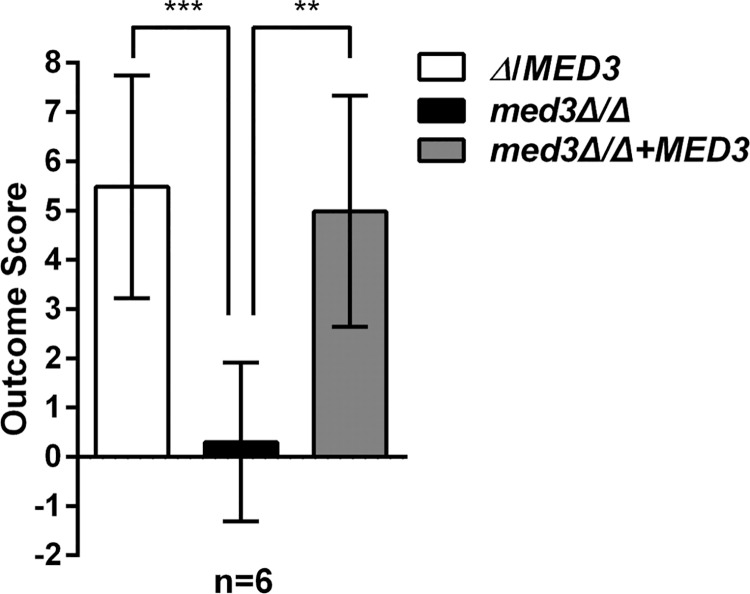
A *C*. *albicans* med3 null strain has attenuated virulence in a murine model for disseminated Candidiasis. Equal innocula of a *med3* heterozygous null strain (yLM116), homozygous null strain (yLM119) and complemented strain (yLM120) were injected into mouse tail veins and the outcome score is calculated from fungal kidney burden and body weight [42]. The outcome score correlates inversely with 28 day survival. The outcome score in the null mutant was compared to a heterozygous null and a complemented null mutant using a t test. Bars represent means ± SD. *, P ≤ 0.05; **, P ≤ 0.01; ***, P ≤ 0.001. The kidney burden and weight change used to calculate the outcome scores were *Δ*/*MED3*(-1.9±3.7%, 4.5±0.6 log_10_ CFU/g), *med3Δ/Δ* (3.8±2.6%, 2.2±0.7 log_10_ CFU/g), and *med3Δ/Δ*+*MED3* (-0.9±3.6%, 4.5±0.8 log_10_ CFU/g).

Fluorescence microscopy revealed that overexpressed GFP-tagged versions of CdTlo1p, CaTloα12p, and CdTlo2p were all localized to the nucleus in a wild type *C*. *dubliniensis* background (Fig 9). CdTlo1p, which is quickly degraded in the ‘free’ form (Fig 2), is cleanly localized to the nucleus, while CaTloα12p and CdTlo2p were focused in the nucleus with some remaining signal in the cytoplasm (Fig 9). Performing this same experiment in a *med3Δ/Δ C*. *dubliniensis* strain revealed that *MED3* was required for nuclear localization of overexpressed CdTlo2p-GFP in *C*. *dubliniensis* (Fig 9). In the case of CdTlo1p-GFP and CaTloα12-GFP overexpression in *C*. *dubliniensis*, the absence of Med3 appears to decrease, but not completely abrogate, its nuclear localization. The absence of both CdTlo2p nuclear localization (Fig 9) and *CdTLO2* overexpression phenotypes (Fig 8) in the *med3Δ/Δ C*. *dubliniensis* strain suggests that these two phenomena may be linked. From a structural standpoint, it is interesting to note that the nuclear localization of Tlo proteins and Med3 is mutually dependent as CdMed3 fails to localize to the nucleus in a *tlo1Δ/Δ C*. *dubliniensis* strain (S21 Fig). Earlier work has shown that the endogenous α and β clade CaTlos are nuclear localized [37]. Here, we again used fluorescence microscopy to determine whether this nuclear localization of the endogenous free population of CaTlos in *C*. *albicans* is dependent on subunits required for Mediator association. We have previously shown that the Med3 and Med15 subunits, but not the Med16 subunit, of the *C*. *albicans* Mediator Tail module are required for the CaTlo subunit to be incorporated into the complex [26]. The nuclear localization of the endogenous CaTlo proteins was dependent on Med3 and Med15, but not Med16 (Fig 10). In both *C*. *dubliniensis* and *C*. *albicans* there is a direct correlation between Mediator association and nuclear localization for the ‘free’ Tlo population. Deletion of *C*. *albicans MED3*, which is required for nuclear localization, also leads to a strain that has highly attenuated virulence in the murine model for disseminated *candidiasis* (Fig 11). We currently don’t know, however, whether it is the lack of CaTlo nuclear localization, the multiple morphological defects present in the *C*. *albicans med3Δ/Δ* strain [26], or the combination of the two that result in attenuated virulence in the *med3* null strain.

### Nuclear localization of the CdTlo2 TAD is necessary and sufficient for the overexpression phenotypes

**Fig 12:**
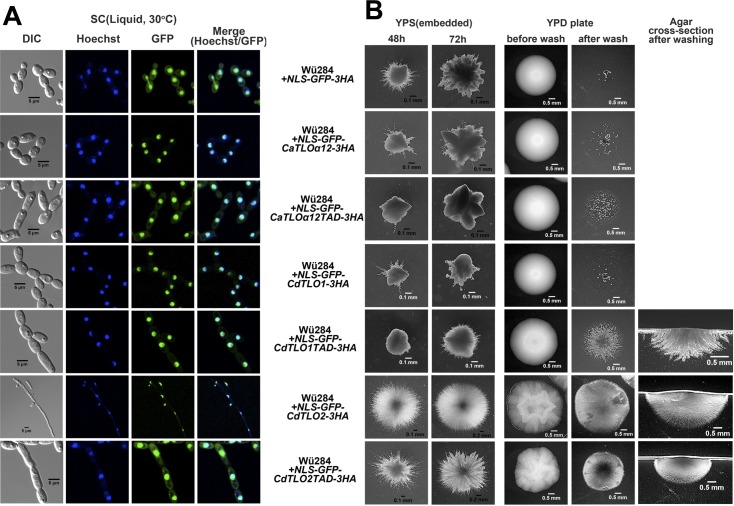
Nuclear localization of the overexpressed transcriptional activation domain of CdTlo2 in *C*. *dubliniensis*, by fusion of an NLS to its N-terminus, facilitates embedded agar and agar invasion phenotypes. **(A)** *TLO* constructs, which were N-terminally fused with NLS-GFP coding sequence and C-terminally HA-tagged, were overexpressed from a *TDH3* promoter in a wild type (Wü284) *C*. *dubliniensis* strain (yLM362 for over-expression of *NLS-GFP-3HA*, yLM363 for *CaTLOα12*, yLM365 for *CdTLO1* and yLM367 for *CdTLO2*). The *CaTLOα12TAD* strain (yLM364) contained residues 164–252 of *CaTLOα12*. The *CdTLO1TAD* strain (yLM366) contained residues 199–320 of *CdTLO1*. The *CdTLO2TAD* (yLM368) strain contained residues 255–367 of *CdTLO2*. Differential contrast (DIC) and fluorescence microscopy were used to visualize GFP localization, while Hoechst staining was used to stain the nuclei. All cells were grown in synthetic complete media overnight, diluted into the same media and grown for 5–6 hours before visualization. **(B)** Embedded agar filamentation (two left columns) and agar invasion (three right columns) phenotype analysis with NLS-GFP *C*. *dubliniensis* strains described in A.

**Fig 13:**
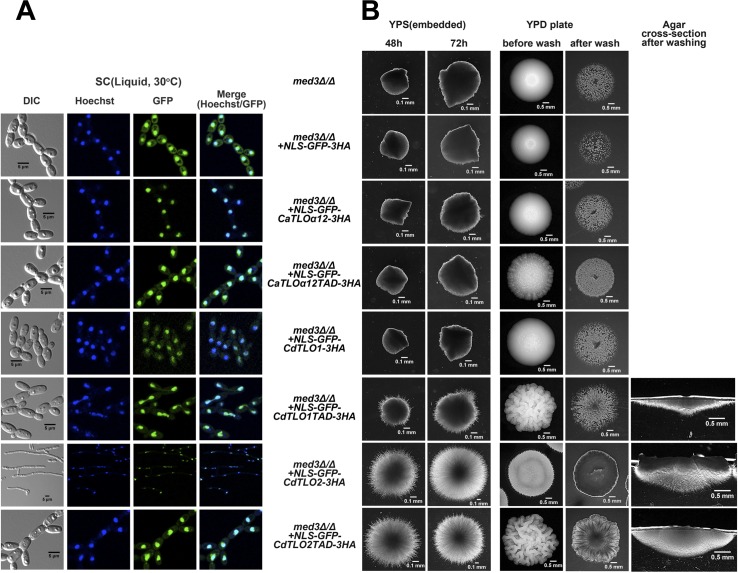
Nuclear localization of the overexpressed transcriptional activation domain of CdTlo2 in a *med3Δ/Δ C*. *dubliniensis* by fusion of a NLS to its N-terminus facilitates embedded agar and agar invasion phenotypes. **(A)** *TLO* constructs, which were N-terminally fused with NLS-GFP coding sequence and C-terminally HA-tagged, were overexpressed from a *TDH3* promoter in a *med3Δ/Δ C*. *dubliniensis* strain (yLM377 for over-expression of *NLS-GFP-3HA*, yLM378 for *CaTLOα12*, yLM380 for *CdTLO1* and yLM382 for *CdTLO2*). The *CaTLOα12TAD* strain (yLM379) contained residues 164–252 of *CaTLOα12*. The *CdTLO1TAD* strain (yLM381) contained residues 199–320 of *CdTLO1*. The *CdTLO2TAD* strain (yLM383) contained residues 255–367 of *CdTLO2*. Differential contrast (DIC) and fluorescence microscopy were used to visualize GFP localization, while Hoechst staining was used to stain the nuclei. All cells were grown in synthetic complete media overnight, diluted into the same media and grown for 5–6 hours before visualization. **(B)** Embedded agar filamentation (two left columns) and agar invasion (three right columns) phenotype analysis with NLS-GFP *C*. *dubliniensis* strains described in A.

Based on the CdTlo2 TAD requirement for *CdTLO2* overexpression phenotypes (Fig 7), and the correlation between nuclear localization and these phenotypes (Figs 8 and 9), we hypothesized that appending a nuclear localization signal (NLS) to the CdTlo2 TAD would be sufficient to confer *CdTLO2* overexpression phenotypes. Fusion of an NLS-GFP sequence to various Tlo ORF fragments resulted in a highly focused nuclear localization (Fig 12A), even in the absence of the Med3 subunit (Fig 13A). The NLS driven nuclear localization is far more efficient than Mediator-dependent localization, as these strains exhibited almost none of the residual cytoplasmic accumulation observed in *CdTLO2-GFP* overexpression strains (Fig 9). NLS driven nuclear localization of 1X overexpression constructs, expressing *GFP-CdTLO2* and *GFP-CdTLO2* TAD, gave potent filamentation phenotypes (Fig 12B) that resembled the *CdTLO2* SW phenotypes (Fig 5). These phenotypes included the constitutive filamentous growth in liquid media. Unlike the *CdTLO2* SW phenotypes observed earlier, the ‘SW-like’ phenotypes in the *NLS-GFP-CdTLO2* strains were not accompanied by increased copy numbers of the *NLS-GFP-CdTLO2* overexpression construct (S22 Fig). This finding suggests that although a larger amount of total CdTlo2 protein in the cell can amplify filamentation phenotypes, the most important factor is the amount in the nucleus. Nuclear localization of the *GFP-CdTLO1* TAD gave a weak agar invasion phenotype, but still had no effect on filamentation in embedded agar (Fig 12). NLS driven nuclear localization of GFP alone, GFP-CaTloα12p, GFP-CaTloα12TAD, GFP-CdTlo1p had no effect on agar invasion or filamentation in embedded agar (Fig 12). Both the NLS and ‘non-NLS’ driven phenotypes were documented in strains that had a C-terminal 3HA epitope tag, which allowed for monitoring protein expression and stability. Repetition of the NLS and ‘non-NLS’ driven phenotypes in strains which overexpress *CdTLO2* constructs lacking the 3HA tag, demonstrated that the epitope tag was not required for the effect (S23 Fig). We speculated that the previously observed deficiency of *CdTLO2* overexpression phenotypes in a *med3Δ/Δ C*. *dubliniensis* strain (Fig 8) was caused by the lack of nuclear localization (Fig 9), rather than by an indirect effect of *med3* deletion on transcription. We tested this idea using the NLS fusions to drive nuclear localization of *GFP-CdTLO2* in a *med3Δ/Δ C*. *dubliniensis* strain. The embedded agar filamentation and agar invasion phenotypes that resulted from *NLS-GFP-TLO* overexpression in the wild type background (Fig 12) were recapitulated in the absence of *MED3* (Fig 13). The dispensability of the Tail module for the *CdTLO2-TAD* driven overexpression phenotypes, under conditions in which the activation domain was localized to the nucleus via a dedicated NLS, was further demonstrated by the presence of these phenotypes in a *C*. *dubliniensis* strain that overexpressed *NLS-GFP-CdTLO2-TAD* in a *tlo1Δ/Δ* background (S24 Fig). Considered as a whole, these observations support a paradigm in which the Mediator Tail module is a vehicle to bring CdTlo2 into the nucleus so its TAD can promote greater sensitivity to induction of filamentation.

## Discussion

Comparative genomics of the closely related fungi *C*. *albicans* and *C*. *dubliniensis* [35] provided the foundation for mechanistic studies to elucidate the origin of *C*. *albicans*’ enhanced virulence and phenotypic plasticity [11] versus *C*. *dubliniensis*. The presence of a large ‘free’ Tlo population, and the presence of a mixture of Mediator complexes with different Tlo subunits [26] were both potential explanations for how *TLO* amplification in *C*. *albicans*, versus *C*. *dubliniensis*, might impact virulence gene expression. Two central considerations led us to pursue the large ‘free’ population as the primary mechanism by which *TLO* amplification could influence the virulence properties of *C*. *albicans* at the transcriptional level. First, the observation that the *CaTLOs* are themselves transcriptionally regulated by pathways that impact virulence [43,44] suggested that modulation of the size of this free pool could play a role in these processes. Second, our recent discovery that the Tlo proteins contained a potent TAD [38] suggested that the large nuclear pool of Tlo protein, which possessed no recognizable DNA binding domain, could impact gene expression by competing for the targets of sequence-specific DNA bound transcriptional activators. The sequestration of activator targets off chromatin by overexpression of a TAD, or ‘squelching’ as this phenomenon has been called, has been demonstrated in artificial [45,46], and a limited number of physiological [47] systems. The ‘free’ pool of Tlo TAD could impact the interactions between DNA-bound transcriptional activators and their co-activators targets [24,48]. They could also impact interactions with co-repressors that negatively regulate transcription by ‘masking’ the TAD of certain DNA-bound transcriptional activators [49] and preventing co-activator interactions. Our finding that engineering a large ‘free’ population of Tlo protein in *C*. *dubliniensis*, through overexpression of *CdTLO2*, enables filamentous growth provides a mechanistic explanation for how genomic differences between *C*. *dubliniensis* and *C*. *albicans* could manifest themselves at the phenotypic level.

Several key findings reported here, support the Tlo TAD competition model and provide the first direct evidence that the ‘free’ population of *C*. *albicans* Tlo proteins resulting from gene amplification could impact virulence related phenotypes in the pathogen. Although we have focused on filamentation in this report, the Tlo TAD competition model supported by our studies could provide for a general plasticity in gene expression that is typically associated with *C*. *albicans* virulence. This study has shown that there are three key properties for an overexpressed *TLO* gene to impact filamentous growth in *C*. *dubliniensis*. First, it must be stable in a non-Mediator associated form. Second, it must possess a transcriptional activation domain that has, presumably, evolved to interact with specific co-activator and co-repressor targets in its native species. And third, it must possess a mechanism to efficiently transport the TAD to the nucleus where it can interact with these targets.

The N-terminal sequence of the Tlos is highly conserved, yet we have found that the primary expressed Tlo protein in *C*. *dubliniensis*, CdTlo1p, possesses instability in the ‘free’ form relative to CaTloα12 and CdTlo2. We have narrowed this property down to sequences in the first and third Helix in the predicted structure of the CdTlo1 N-terminus. The finding that CdTlo1 degradation is proteasome dependent suggests it might be a target for ubiquitylation. We have not, however, been able to find a particular amino acid in the CdTlo1 N-terminus that confers this property or direct evidence for ubiquitylation. It is possible that this intrinsic instability of CdTlo1 may have prevented the amplification of the Tlo genes in *C*. *dubliniensis*. If there were a selective advantage for the accumulation of a ‘free’ Tlo pool, amplification of *CdTLO1* would never have conferred this advantage. Since CdTlo2 expression is inherently very low [28,35], amplification of this copy would also not have conferred any selective advantage. The failure of our stabilized hybrid Tlo1 protein (HyNT1C), however, showed that stability alone was not enough to confer the overexpression phenotypes observed with *CdTLO2*.

Our earlier studies had shown that the C-termini of CaTlo α and β, the CdTlo, and the *S*. *cerevisiae* Med2 (Tlo ortholog) proteins all contain potent transcriptional activation domains [38]. Of the CaTloα12, CdTlo1 and CdTlo2 TADs, however, we found only the CdTlo2 TAD can drive filamentation phenotypes in *C*. *dubliniensis*. TADs are typically unstructured and highly modular, in that appending them to any variety of DNA binding domains will lead to the increased expression of a reporter gene placed next to a binding site for the particular DNA binding domain [50,51]. The inability of the CaTlo proteins, and CdTlo1 TAD (appended to either the N-termini of the stabilized hybrid Tlo1 protein (HyNT1C) or the inherently stable CdTlo2) to confer the overexpression phenotypes indicates that possessing potent activity in our one-hybrid assay in *C*. *albicans* and *S*. *cerevisiae* is not sufficient for promoting filamentation. The activation domains of CaTloα12, CdTlo1 and CdTlo2 all have activation potential that equals or exceeds the prototypical VP16 activation domain when measured in *S*. *cerevisiae* [38]. Of these, the CdTlo2p activation domain potential was highest. The activation domains of CaTloα12 and CdTlo1 showed a similar pattern in a *C*. *albicans* one-hybrid reporter system. These findings suggest that there are particular *C*. *dubliniensis* co-activators, or co-repressors, that strongly interact with the CdTlo2 TAD compared to the TADs of CdTlo1 and CaTloα12. We infer from this result, that a converse situation may take place in *C*. *albicans* in which there are specific targets of the CaTlo TADs that do not interact as strongly with the CdTlo2 TAD. This specificity will facilitate future efforts to identify these targets.

Our model for the competition of the ‘free’ Tlo population with DNA-bound transcription factors for co-activator and co-repressor targets requires that these interactions take place in the nucleus. Prior to this study, however, very little was known about how individual or groups of Mediator subunits are imported into the nucleus. Studies in *S*. *cerevisiae* have shown that the fungal Tail module subunits Med2 (Tlo), Med3 and Med15 can exist as a stable trimer, both within the context of Mediator [32] and independently [52]. Our studies show that there is substantial co-dependence among these subunits for their nuclear import as well. Previous studies, which have tried to interpret the consequence of deletion of Mediator subunits on the phenotype, work under the tacit assumption that the nuclear localization of the remaining subunits persists unchanged in these deletion mutants [30]. Our results show that, at least in some instances, this is clearly not the case and localization of additional subunits should be taken into account when interpreting these results. The inability of Tlo protein to get into the nucleus in the absence of interactions with Med3 (and Med15) subunits allowed us to perform incisive experiments on the importance of the Tlo TAD. Appending an NLS to the CdTlo2 TAD, in the absence of its Mediator associating N-terminal domain, showed that the CdTlo2 TAD was sufficient to drive the filamentation phenotypes, while the CaTloα12 TAD was not. The ability of *NLS-GFP-CdTLO2*, but not *CdTLO2*, overexpression to drive filamentation phenotypes in a *med3Δ/Δ C*. *dubliniensis* strain demonstrates nuclear localization of the Tlo protein is necessary for these phenotypes. The CdTlo1 TAD appeared to have a weak agar invasion phenotype when fused to the NLS-GFP construct, even though the filamentation phenotypes were absent when the CdTlo1 TAD was fused to the CdTlo2 or HyNT1C N-termini. This result, the fluorescence microscopy studies, as well as the fact that the *NLS-GFP-CdTLO2(TAD)* overexpression phenotypes mimicked the phenotypes achieved at the highest levels of CdTlo2 overexpression (the SW phenotypes), suggests that high levels of nuclear localization are the key driver of overexpression phenotypes. The molecular origin of the *CdTLO2* ‘SW’ phenotypes appears to originate from gene amplification without increase in chromosome 7 ploidy, but it does not appear to include increased nuclear localization of CdTlo2 per se (S25 Fig). The finding that the *NLS-GFP-CdTLO2* strains generate a phenotype similar to the ‘SW’ strains suggests that this phenomenon is connected most directly to the increase in *CdTLO2* expression rather than the location of the second insertion. Although both *NLS-GFP-CdTLO2* and *NLS-GFP-CdTLO2TAD* overexpression resulted in filamentation phenotypes, the phenotypes differed from each other. Our earlier studies generally showed that full length Tlo/Med2 constructs had a weaker activation potential than their TAD by itself [38]. It is consistent with our model that variation of the activation potential could lead to varied phenotypes.

We speculate that the mechanism used by the ‘free’ Tlo protein in *C*. *albicans* evolved from properties that were inherently important for the Tlo/Med2 subunit in the context of the intact Mediator complex. Our previous studies of *S*. *cerevisiae* and *C*. *dubliniensis* Mediator showed that the presence of transcriptional activation domains in Tail module subunits of Mediator were important for the Tail module to facilitate the Mediator dependent induction of certain genes [38]. When the gene encoding this subunit became amplified in *C*. *albicans*, Mediator membership conferred two critical properties, a TAD and nuclear localization, necessary to impact the transcriptional programing of the cell. The mechanism by which Mediator membership facilitates the accumulation of a pool of ‘free’ Tlo protein is still a black box. Exchange of Mediator associated Tlo subunits is likely to be required for one molecule of Mediator to bring in multiple molecules of Tlo protein and accumulate a ‘free’ Tlo population. Since the N-termini of the *C*. *albicans* paralogs are nearly identical, it is likely that the ancestral *C*. *albicans* Tlo protein also possessed the property of being stable outside the context of association with Mediator prior to amplification. The ancestral *C*. *albicans* Tlo Mediator subunit was primed to take on its new role in its amplified form. It is unclear, however, what evolutionary history led to *C*. *dubliniensis* having two Tlo paralogs versus one in all other sequenced fungi besides *C*. *albicans*.

The results of our study beg the question whether *TLO* amplification represents a special case or could overexpression/amplification of Mediator subunits and/or TAD containing proteins represent a broader regulatory mechanism in health and disease. Amplification dependent and independent overexpression of the human Mediator Cdk8 kinase subunit, or other members of the Cdk8 module, occurs in a large percentage (>50%) of colon, breast, and gastric cancers and is proposed to act as an oncogene [53]. An additional Mediator subunit, *S*. *cerevisiae* Med3 [38], contains a TAD. Although no amplification dependent overexpression of Tlos, or other TAD containing proteins, had been identified in other fungal pathogens, it is still possible that future studies could find that amplification independent overexpression of Tlo/Med2 or Med3 orthologs could contribute to their virulence.

## Materials and Methods

### Strain construction

The *C*. *dubliniensis* and *C*. *albicans* strains used in this study are listed in the **S2**and **S3 Tables** respectively. Each *C*. *dubliniensis* strain over-expressing *TLO* variants, listed in the **S2 Table**, was generated by transforming the parental strain with the indicated integrating plasmid by electroporation. The plasmids and the restriction enzymes used to release the integrative DNA cassettes from each plasmid are listed in the **S4 Table**. The sequences of the primers used to verify the correct insertion of the cassette at the *TLO1* genomic locus are listed in the **S5 Table**. Each *C*. *albicans* strain overexpressing a *TLO* variant, listed in the **S3 Table**, was generated by transforming the parental strain with the indicated integrating plasmid by electroporation. The plasmids and the restriction enzymes used to release the integrative DNA cassettes from each plasmid are listed in the **S4 Table**. Their appropriate insertion at the *RPS10* locus was confirmed by the primers pairs, whose sequences are listed in the **S5 Table**. Additional strains in the **S2**and **S3 Tables** were created by introducing certain PCR products into the genomes of *C*. *dubliniensis* or *C*. *albicans*. Specifically, DNA cassettes amplified by ZL422/ZL423 from *pFA-3HA-SAT1* [26] was used to tag endogenous *TLO1* in *Cdmed3Δ/Δ* to generate yLM308. C-terminal 3XHA tagging of endogenous *TLOα12* in *C*. *albicans* SN152 (resulting in yLM388), *TLOα34* in *C*. *albicans* SN152 (resulting in yLM391) and *C*. *albicans med3Δ/Δ* (resulting in yLM392), were performed by transforming the strains with PCR product amplified from *pFA-3HA-SAT1* by KPP035/KPP037 (targeting *TLOα12*) or KPP035/KPP041 (targeting *TLOα34*) accordingly. Deleting one or both alleles of *MED3*, and complementing the *med3* null mutant in SN152 was achieved as described [26].

### Cell growth and treatment

If not otherwise specified, *C*. *dubliniensis* and *C*. *albicans* strains were grown in YPD (2% Bacto Peptone (BD), 1% Bacto Yeast Extract (BD) and 2% Glucose) liquid media or maintained on YPD agar plate at 30°C. All the YP-based media used in this study were supplemented with 100 μM uridine. Steady-state protein levels of overexpressed *TLO* variants were compared using mid-log phase cells grown in YPD liquid culture.

For confocal microscopy, mid-log phase cells grown in YPD media were used for Blankophor staining (0.2% (wt/vol) Blankophor; 5 minutes at room temperature). To monitor GFP-tagged protein localization, mid-log phase cells, grown in SC media (6.7 g/L Difco YNB without amino acids (BD), 2 g/L Drop-out Mix Synthetic without uracil (US Biological), 2% Glucose, 200 μM uridine), was stained with 100 μg/mL of Hoechst 33342 with ~30 minutes incubation at room temperature in the dark without agitation before imaged by confocal microscopy with a Nikon A1 microscope.

To assess protein stability, overnight cultures were diluted to OD_600_ = 0.2 in fresh YPD media and grown for 2–3 cell divisions. Cycloheximide (10 mg/mL freshly dissolved in YPD) was added to a final concentration of 2 mg/mL [40] after saving an aliquot as the ‘0 min’ sample. Samples were collected at each indicated time point, washed and flash-frozen in liquid nitrogen.

MG132 inhibition of the proteasome degradation pathway in *C*. *dubliniensis* and *C*. *albicans* was performed following the previously described protocol established in *S*. *cerevisiae* [54]. Specifically, the indicated *Candida* strains were grown overnight in synthetic media (1.7 g/L YNB without ammonium sulfate, 1 g/L L-proline, 2% glucose, 200 μM uridine, 20 mg/L L-Histidine, 20 mg/L L-Arginine, 100 mg/L L-Leucine), diluted in the same media to OD_600_ = 0.2, and further grown for ~2 cell divisions. Cultures were supplemented with SDS to a final concentration of 0.003%, grown for 4 hours, and split into two for addition of MG132 (42 mM in DMSO, APExBIO) to a final concentration of 40 μM, or an equal volume of DMSO. After treatment for 45–60 minutes, cells were pelleted by centrifugation and directly flash-frozen in liquid nitrogen without any washing step, or further treated with cycloheximide (dissolved in the specified synthetic media instead of in YPD) as described above to monitor protein half life.

### Spot dilution growth assays

*C*. *dubliniensis* strains were grown overnight in YPD. After washing, the cells were diluted to 3X10^6^, 3X10^5^ 3X10^4^ and 3X10^3^ cells/mL and spotted on YP (2% peptone, 1% yeast extract, 100μM uridine)-based 2% agar plates. Plates were incubated at 30°C if not otherwise specified.

### Cell/colony morphology assays

To assess agar invasive growth 200 μL (~1 cell/μL) diluted overnight culture of each strain was spread on an YPD agar plate. After incubation at 30°C for 4 days, representative colony morphologies were imaged under a Nikon SMZ1500 stereoscope. Surface cells were removed by washing the plates under distilled water, and the agar-invasive growth was imaged directly or after cross-sectioning an agar slice.

Embedded filamentation assays were performed as described [55] with modifications. Specifically, cells from a fresh colony were grown overnight in liquid YPD media, diluted into 3 mL fresh media (~1000 cells/mL), and further grown for 4 hours at 30°C. 100 μL of this culture was mixed with ~25 mL of lukewarm (~45°C) YPS (2% Bacto Peptone (BD), 1% Bacto Yeast Extract (BD), 2% sucrose, 2% Bacto Agar (BD), 0.1 mM uridine) media and poured into a Petri dish. Plates were incubated at 30°C and the embedded colonies with representative morphology were imaged by Nikon SMZ1500 stereoscope at 48h and 72h. Images representing the embedded hyphae development at different time points are not necessarily taken from the same colony within the agar.

### Protein purification

Affinity purification of C-terminal 6His3FLAG tagged Tlo protein variants to test for incorporation into intact CdMediator complex was performed as described previously [26], except the TALON step was omitted. For pull down experiments, a crude cell lysate of yLM416 was fractioned by Heparin Sepharose and the fraction which enriched with CdTlo2 and Med1 was incubated with Protein G Dynabeads which have been pre-coated either with anti-Med1 antibody or a non-related anti-HA antibody (Santa Cruz, F-7) for 2 hours at 4°C. Mediator complex purified though 6His3FLAG-tagged Tlo1 (as prepared [38]) was used as the standard for subunit stoichiometry and treated by the same depletion process.

### Immunoblotting

To monitor the levels of Tlo variants in whole cell lysates, samples were prepared from frozen cell pellets (~4–5 OD of cells) following the ESB method [56], resolved by 10% SDS-PAGE and then either stained with coomassie blue or blotted by the indicated antibody [anti-HA (Roche, 3F10) or anti-FLAG (Sigma, F7425)] as described previously [57]. Western blot signals of given samples were measured by ImageQuant (Molecular Dynamics) and normalized to their relative total protein concentration, which was quantified by the total coomassie staining signals in the same gel area cross the samples (measured by UN-SCAN-IT (Silk Scientific)). *Ca/Cd*Med1p antibody was the same rabbit polyclonal antibody generated against recombinant *C*. *albicans* Med1p in [26]. This antibody showed cross-reactivity to *C*. *dubliniensis* Med1p was used to monitor CdMed1 in semi-purified or purified CdMediator samples.

### RT-qPCR

RNA samples were prepared from frozen cell pellets collected from mid-log phase cultures in YPD, and reverse-transcribed as described previously [27]. qPCR was performed using ‘Relative Standard Curve’ method (StepOne, Life Technologies). Relative *Ca/CdACT1* level, quantified by AZq026/AZq027, was used as the internal reference between samples. *TEF1* level (measured by ZL386/ZL387), instead of *ACT1*, was used as the reference when *TLO2-3HA* mRNA abundance was compared between *C*. *dubliniensis* strains with ‘super-wrinkled’ morphology versus their smooth counterparts, because *ACT1* is reported to be differentially expressed in hyphae and yeast state [58,59]. mRNA levels of C-terminal 3XHA tagged *TLO* variants driven by an overexpression promoter, or the endogenous promoter, were measured by using ZL313/ZL201. When *TLO* variants were over-expressed in the non-tagged form, gene specific primers were used to evaluate their expression level: ZL190/191 for *CdTLO1*, ZL315/ZL316 for *CaTLOα12*, ZL190/ZL318 for *HyNT1C* and ZL426/ZL427 for *CdTLO2*.

### Ploidy analysis in *C*. *dubliniensis*

A qPCR-based ploidy analysis for *C. dubliniensis* strains was modified from the method developed in *C. albicans* [60]. Genomic DNA was extracted from strains (yLM339, yLM343, yLM344, yLM345, yLM347 and yLM367) grown in 5 mL YPD liquid media over-night, 25 ng of which was quantified by qPCR using specific primer pairs to determine the copy number of nine representative genomic loci with the parental wild type strain Wü284 as the reference. Details are described in Supplemental Material and Methods section.

### Quantification of *TLO2* copy number in *C*. *dubliniensis TLO2* over-expression strains

*TLO2* copy number in a given strain was determined by qPCR using yLM339, yLM344, and Wü284 as reference strains as described in details in Supplemental Material and Methods.

### Murine model for disseminated *Candidiasis*

The three day murine intravenous challenge model was used to assay fungal virulence as previously described [61]. Immunocompetent female BALB/c mice (6–8 weeks; Harlan, UK) were randomly assigned to groups of 6 and housed separately in individually ventilated cages. Group size was determined from power analyses using data obtained from previous experiments using the same infection model and parental fungal strain. Group size (n = 6) is the minimum groups size required to determine statistically significant differences in the parameters measured, where P ≤0.05, Power = 0.8. Food and water were provided *ad libitum*. *C*. *albicans* was grown overnight in NGY medium at 30°C with shaking. Fungal cells were harvested, washed, resuspended in sterile saline, and cells were counted. Approximately 1x10^6^ cells were injected into each mouse via the lateral tail vein. The mice were monitored and weighed daily. At 72 h post-infection, mice were weighed, killed humanely by cervical dislocation, and their kidneys removed aseptically for determination of fungal burdens. Virulence outcome scores were determined by assessing renal fungal burden and percentage weight change at 72 h using the formula: outcome score = log (renal CFU g^-1^)—(0.5 × percentage weight change) [61]. Results were compared using Kruskal-Wallis comparison across all data sets and Mann-Whitney U tests for pair-wise comparisons using IBM SPSS (version 23) * P ≤0.05; ** P ≤0.01; *** P ≤0.001.

### Ethics statement

Animal experimentation was performed under UK Home Office Project license 60/4135, which was approved by the UK Home Office and by the University of Aberdeen Animal Welfare and Ethical Review Body (AWERB). All work conformed to European Directive 2010/63/EU.

During the colonization and infection studies, animals were monitored carefully for signs of illness and distress. Suffering was minimized by expert handling. Animals were monitored for changes in condition at least twice per day, and were weighed once per day. If animals had shown signs of severe illness (e.g. ruffled coat, hunched posture, unwillingness to move and 20% loss of initial body weight) euthanasia would have been performed by cervical dislocation. During these studies there were no animals were euthanized prior to the 72 h sampling time point. Analgesia and anesthesia were not employed in this study.

## Supporting Information

S1 Fig Schematic diagram of transformation cassettes for high level expression of *TLO* genes in *C*. *dubliniensis* and *C*. *albicans*. **(A)** Constructs for integrating one or two copies of a *TLO* gene into the *TLO1* locus in *C*. *dubliniensis*. The constructs bearing a C-terminal 3XHA tag are shown here. In certain experiments, un-tagged or 6His3Flag tagged constructs were used to determine the phenotypes of non-tagged *TLO* variants or to facilitate the affinity purification of a Tlo protein respectively. **(B)** Construct for integrating a *TLO* gene into the *RPS10* locus in *C*. *albicans*. **(C)** Constructs for integrating a combination of Cd*TLO1* and *CaTLOα12* genes into the *TLO1* locus in *C*. *dubliniensis*.
(TIF)

S2 Fig Co-expression of CaTloα12p and CdTlo1p in *C*. *dubliniensis* de-stabilizes CdTlo1p. Immunoblot showing that *TDH3* promoter driven co-expression of HA-tagged *CdTLO1* and Ca*TLOα12* in a *tlo* null *C*. *dubliniensis* strain (yLM306 and yLM307) leads to a decrease in the steady-state level of CdTlo1p when compared to the CdTlo1p level in a strain solely over-expressing CdTlo1 (yLM302). Coomassie blue staining (CBS) was used as a loading control.
(TIF)

S3 Fig Deletion of *MED3* does not change *CdTLO1* mRNA level in *C*. *dubliniensis*. Liquid culture of two independent colonies (‘1’ and ‘2’) from *TLO1* C-terminal HA tagged wild type (yLM301) and *med3* null (yLM308) *C*. *dubliniensis* strains were grown and the RNA extracted. The steady state ratio of *TLO1* mRNA levels to *ACT1* mRNA was determined by RT-qPCR and normalized by setting the measurement of the first colony mRNA to one. The error bars represent the technical deviation of two sets of qPCR measurements on a given sample.
(TIF)

S4 Fig CdTlo1p is unstable, compared to CaTloα12p, when over-expressed in *C*. *albicans*. **(A)** Immunoblot showing that HA-tagged CdTlo1p (yLM390) levels are lower than CaTloα12p (yLM389) levels when expressed from the same strong promoter (*pACT1*) in *C*. *albicans*. Two independent transformants (‘1’ and ‘2’) were tested and compared with two independent *C*. *albicans* transformants, each with one endogenous *TLOα12* tagged by the same 3XHA tag (yLM388). Coomassie blue staining (CBS) was used as a loading control. **(B)** RT-qPCR analysis showing that *CdTLO1* mRNA levels in yLM390 are comparable to *CaTLOα12* levels in yLM389 when expressed from the same strong promoter (*pACT1*) in *C*. *albicans*. Two independent transformants (‘1’ and ‘2’) were tested. The steady state mRNA levels were calculated as a ratio to *ACT1* mRNA and normalized by setting the measurement of one of the endogenous *CaTLOα12* tagged strains (yLM388) to one. The error bars represent the technical deviation of two sets of qPCR measurements on a given sample.**(C)** Plot of fold-change of overexpressed *CdTLO1* and *CaTLOα12* mRNA and protein (from A. and B.) compared to the endogenous levels of *CaTLOα12*. The dashed line represents an idealized slope of 1 in which the fold-change in protein is equal to the fold-change in mRNA.
(TIF)

S5 Fig Non-Mediator associated CdTlo1 protein has a short half-life compared to CaTloα12p. **(A)** Immunoblot of endogenous HA-tagged CdTlo1p in a wild type *C*. *dubliniensis* strain (yLM301) after treatment with cycloheximide (CHX). Coomassie blue staining (CBS) was used as a loading control. **(B)** Immunoblot of endogenous HA-tagged CaTloα34 in a wild type *C*. *albicans* strain (yLM391) after treatment with cycloheximide. Coomassie blue staining was used as a loading control. **(C)** Immunoblot of endogenous HA-tagged CdTlo1p in a *med3Δ/Δ C*. *dubliniensis* strain (yLM308) after treatment with cycloheximide. Coomassie blue staining was used as a loading control. **(D)** Immunoblot of endogenous HA-tagged CaTloα34 in *med3Δ/Δ C*. *albicans* strain (yLM392) after treatment with cycloheximide. Coomassie blue staining was used as a loading control.
(TIF)

S6 Fig Non-Mediator associated CdTlo1 protein has a short half-life compared to CaTloα12p. **(A)** Immunoblot of overexpressed HA-tagged CaTloα12p in a wild type *C*. *albicans* strain (yLM393) after treatment with cycloheximide (CHX). Coomassie blue staining (CBS) was used as a loading control. **(B)** Immunoblot of overexpressed HA-tagged CdTlo1p in a wild type *C*. *albicans* strain (yLM394) after treatment with cycloheximide. Coomassie blue staining was used as a loading control.
(TIF)

S7 Fig Over-expressed CdTlo1 in *C*. *albicans* is stabilized by proteasome inhibition. **(A)** Immunoblot of overexpressed HA-tagged CaTloα12p and CdTlo1p in a wild type *C*. *albicans* strain (yLM389 and yLM390 respectively) in the presence and absence of the proteasome inhibitor MG132. Coomassie blue staining (CBS) was used as a loading control. **(B)** Plot of the increase in CdTloα12p and CdTlo1p upon treatment with MG132 quantified from the blot in part A.
(TIF)

S8 Fig CaTloα12, expressed in *C*. *dubliniensis*, is stably incorporated into the Tlo/Med3/Med15 trimer, but not the entire CdMediator. **(A)** Silver staining SDS PAGE analysis of affinity purified protein form an untagged (Wü284), 6His-3Flag tagged CaTloα12 (yLM309) and 6His-3Flag tagged CdTlo1 *C*. *dubliniensis* strain (yLM415). **(B)** Immunoblot analysis of affinity purified 6His-3Flag tagged CaTloα12 and 6His-3Flag tagged CdTlo1 from *C*. *dubliniensis*. An anti-Med1 antibody is used to show that 6His-3Flag tagged CdTlo1p is able to pull down the Middle module subunit, Med1, but 6His-3Flag tagged CaTloα12p could not in the affinity purified sample in A.
(TIF)

S9 Fig Construction details of chimeric CaTloα12/CdTlo1 proteins used to determine the minimal N-terminal sequence of Tloα12 required to stabilize CdTlo1 when over-expressed in *C*. *albicans* or *C*. *dubliniensis*. **(A)** Detailed schematic of predicted secondary structure of Tloα12 and CdTlo1. **(B)** Detailed schematic of chimeric proteins containing various Tloα12 N-terminal and CdTlo1 C-terminal sequences. In each panel the first three amino-acids (MSA or MPE) are denoted at the beginning of each construct. The segments shaded light grey represent regions of CdTlo1p ‘Helix 3’ that do not have clear homologous sequence in CaTloα12 ‘Helix 3.’
(TIF)

S10 Fig CaTloα12 ‘Helix 3’, but not the C-terminal TAD, is required for its stability when overexpressed in a *C*. *dubliniensis tloΔ/Δ* strain. **(A)** Immunoblot showing over-expression of HA-tagged *CaTLOα12*, C*dTLO1*, and *CaTLOα12/CdTLO1* chimeras from the *TDH3* promoter in a *C*. *dubliniensis tloΔ/Δ* strain. Strains used to generate the data are yLM303(*CaTLOα12*), yLM310(*12NT1C*), yLM311(*12N-1*), yLM312(*12N-2*), yLM313(*12N-3*), yLM314(*12N-4*) and yLM302(C*dTLO1*). Two independent transformants (‘1’ and ‘2’) of each strain were tested. Coomassie blue staining (CBS) was used as a loading control. **(B)** Bottom half contains plot showing relative expression of HA-tagged *CaTLOα12*, C*dTLO1*, and *CaTLOα12/CdTLO1* chimeric proteins (from immunoblot in A.) and mRNA (from RT-qPCR) normalized to *CaTLOα12*, and top half contains shows a plot of protein to mRNA ratios derived from the corresponding data on the bottom half of the graph.
(TIF)

S11 Fig CaTloα12 ‘Helix 3’, but not the C-terminal TAD, is required for its stability when overexpressed in a wild type *C*. *albicans* strain. **(A)** Immunoblot showing over-expression of HA-tagged *CaTLOα12*, C*dTLO1*, and *CaTLOα12/*CdTLO1 chimeras from the *TDH3* promoter in a wild type *C*. *albicans* strain. Strains used to generate the data are yLM389(*CaTLOα12*), yLM395(*12NT1C*), yLM396(*12N-1*), yLM397(*12N-2*), yLM398(*12N-3*), yLM399(*12N-4*) and yLM390(C*dTLO1*). Two independent transformants (‘1’ and ‘2’) were tested for each strain. Coomassie blue staining (CBS) was used as a loading control. **(B)** Bottom half contains plot showing relative expression of HA-tagged *CaTLOα12*, C*dTLO1*, and *CaTLOα12/CdTLO1* chimeric proteins (from immunoblot in A.) and mRNA (from RT-qPCR) normalized to *CaTLOα12*, and top half contains shows a plot of protein to mRNA ratios derived from the corresponding data on the bottom half of the graph.
(TIF)

S12 Fig Construction details of chimeric CaTloα12/CdTlo1 proteins used to determine what minimal C-terminal sequences of CaTloα12 are required to stabilize CdTlo1 when over-expressed in *C*. *albicans*. Detailed schematic of chimeric proteins containing various CaTloα12 C-terminal and CdTlo1 N-terminal sequences. The first three amino-acids (MSA or MPE) are denoted at the beginning of each construct. The segments shaded light grey represent regions of CdTlo1p ‘Helix 3’ that do not have clear homologous sequence in CaTloα12 ‘Helix 3.’
(TIF)

S13 Fig Replacement of CaTloα12 N-terminal residues 1–38, and residues 1–104, with orthologous sequence from CdTlo1 leads to progressive destabilization of overexpressed hybrid proteins expressed in a wild type *C*. *albicans* strain. **(A)** Immunoblot showing over-expression of HA-tagged *CaTLOα12*, C*dTLO1*, and *CdTLO1*/*CaTLOα12* chimeras from the *TDH3* promoter in a wild type *C*. *albicans* strain. Strains used to generate the data are yLM390(*CdTLO1*), yLM400(*T1N12C*), yLM401(*TN-1*), yLM402(*TN-2*), yLM403(*TN-3*), yLM404(*TN-4*), yLM405(*TN-5*), yLM406(*TN-6*), yLM407(*TN-7*) and yLM389(*CaTLOα12*). Two independent transformants (‘1’ and ‘2’) were tested. Coomassie blue staining (CBS) was used as a loading control. **(B)** Bottom half contains plot showing relative expression of HA-tagged *CaTLOα12*, C*dTLO1*, and *CdTLO1/CaTLOα12* chimeric proteins (from immunoblot in A.) and mRNA (from RT-qPCR) normalized to *CaTLOα12*, and top half contains shows a plot of protein to mRNA ratios derived from the corresponding data on the bottom half of the graph.
(TIF)

S14 Fig Analysis of affinity purified 6His-3Flag tagged CaTloα12/CdTlo1 chimeric proteins demonstrates ‘Helix 2’ of CdTlo1 is required for stable incorporation of chimeric Tlo proteins into *C*. *dubliniensis* Mediator. Immunoblot analysis of cell lysate, heparin purified, and Flag-purified samples derived from a *C*. *dubliniensis tloΔ/Δ* strain over expressing 6His-3Flag tagged CdTlo2 (yLM327) and CaTloα12/CdTlo1 chimeric proteins including *T1N12C*(yLM315), *TN-1*(yLM316), *TN-2*(yLM317), *TN-4*(yLM318), *TN-5*(yLM319), *TN-7*(yLM320), *12NT1C*(yLM321), *12N-1*(yLM322), *12N-3*(yLM323), *12N-4*(yLM324), *T12H*_*2*_(yLM325) and *12NTH*_*2*_(yLM326). There is a contaminant (*) that cross reacts with the α-Flag antibody in the cell lysate and heparin stages of the isolation. An α-Med1 antibody is used to track the presence of intact Mediator. Coomassie blue staining (CBS) was used as a loading control. The final Flag-purified 6His-3Flag tagged CaTloα12/CdTlo1 chimeric proteins are analyzed by silver stain analysis to reveal the presence of Mediator subunits from the Middle, Head and Cdk8 modules. Arrow heads point to a silver stained band representing the chimeric protein.
(TIF)

S15 Fig HyNT1C protein is stable when overexpressed in *C*. *albicans* and *C*. *dubliniensis*. (A) Immunoblot showing over-expressed HA-tagged *HyNT1C* in *C*. *albicans* (yLM408) led to accumulation of protein equivalent to overexpression of *CaTLOα12* (yLM389) and exceeding *CdTLO1*(yLM390). Two independent transformants (‘1’ and ‘2’) were tested. Coomassie blue staining (CBS) was used as a loading control. **(B)** Immunoblot showing that *TDH3* promoter driven co-expression of HA-tagged *HyNT1C* and *CdTLO1* in a *tlo* null *C*. *dubliniensis* strain (yLM330), leads to a decrease in the steady-state level of CdTlo1p when compared to CdTlo1p levels in a strain (yLM302) solely over-expressing *CdTLO1*. Two independent transformants (‘1’ and ‘2’) were tested. Coomassie blue staining (CBS) was used as a loading control.
(TIF)

S16 Fig CdTlo2 overexpression isolates possessing the super wrinkled (SW) phenotype have 4 or more copies of the *pTDH3-CdTLO2* construct. **(A)** Radar plot shows extra copies of *TLO2-HA* ORF and *SAT1* ORF are present in 3 independent ‘SW’ (yLM343) isolates compared with an isolate of their smooth (yLM339) counterpart when wild type *C*. *dubliniensis* (Wü284) is transformed with the *CdTLO2-HA*_*1x*_ cassette (one copy of *TLO2-HA* driven by a *TDH3* promoter). Genomic DNA extracted from an overnight YPD culture of the indicated strains was quantified for exogenous *TLO2* and *SAT1* copy number by qPCR using primer pairs annealing to different regions within the *TLO2* ORF. *SAT1#* is the number of copies of the *SAT1* cassette; TLO2-3HA# is the number of HA-tagged *CdTLO2* copies measured using one primer to the HA-tag coding sequence and one primer to the *CdTLO2* coding sequence; and exogenous *TLO2#* is the number of copies of *CdTLO2* using one of two primer pairs (‘1’ and ‘2’) minus the two endogenous *CdTLO2* copies. The *CdTLO2-HA*_*2x*_ (two copies of *TLO2-HA* each driven by a *TDH3* promoter) smooth transformant (yLM344) and the parental wild type *C*. *dubliniensis* (Wü284) are also included as reference. **(B)** Radar plot shows extra copies of *TLO2-HA* ORF and *SAT1* ORF are present in 3 independent ‘SW’ (yLM345) isolates compared with an isolate of their smooth (yLM344) counterpart when wild type *C*. *dubliniensis* (Wü284) is transformed with the *CdTLO2-HA*_*2x*_ cassette (two copies of *TLO2-HA* driven by a *TDH3* promoter with a single SAT1 cassette). Genomic DNA extracted from an overnight YPD culture of the indicated strains was quantified for exogenous *TLO2* and *SAT1* copy number by qPCR using primer pairs annealing to different regions within the *TLO2* ORF. *SAT1#* is the number of copies of the *SAT1* cassette; TLO2-3HA# is the number of HA-tagged *CdTLO2* copies measured using one primer to the HA-tag coding sequence and one primer to the *CdTLO2* coding sequence; and exogenous *TLO2#* is the number of copies of *CdTLO2* using one of two primer pairs (‘1’ and ‘2’) minus the two endogenous *CdTLO2* copies. The *CdTLO2-HA*_*2x*_ (one copy of *TLO2-HA* driven by a *TDH3* promoter) smooth transformant (yLM339) and the parental wild type *C*. *dubliniensis* (Wü284) are also included as reference. **(C)** Scatter plot summarizes the exogenous *TLO2* ORF copy number in various *C*. *dubliniensis TLO2* over-expression strains with different colony morphology (see parts A. and B.).
(TIF)

S17 Fig Increased copies of *CdTLO2* in SW strains are not a result of increased Chromosome 7 ploidy compared to other *C*. *dubliniensis* chromosomes. Radar plots showing relative ploidy analysis of: **(A)** the parental wild type Wü284 *C*. *dubliniensis* strain; **(B)** a *C*. *dubliniensis* strain over-expressing one copy (1X) of HA-tagged *TLO2* driven by a *TDH3* promoter with smooth colony morphology (yLM339); **(C)** a *C*. *dubliniensis* strain over-expressing two copies (2X) of HA-tagged *TLO2* each driven by a *TDH3* promoter with smooth colony morphology (yLM344); **(D)** nine independent transformants with ‘super-wrinkled’ morphology obtained by transforming Wü284 with the *TLO2-HA*_*1X*_ cassettes (yLM343); **(E)** three independent transformants with ‘super-wrinkled’ morphology obtained by transforming Wü284 with the *TLO2-HA*_*2X*_ cassettes (yLM345); and **(F)** two independent transformants with ‘super-wrinkled’ morphology obtained by transforming Wü284 with a DNA cassette containing two copies of *TDH3* promoter-driven non-tagged *TLO2* (yLM347). A value of ‘1’ indicates a given strain has kept the same copy number at a tested chromosomal locus compared to the parental Wü284 strain, while ‘0.5’ and ‘1.5’ respectively indicate missing and acquiring one copy of a given locus during strain construction. Nine chromosomal loci were tested for each strain and annotated as Chr(Chromosome)_’# (chromosome in the assembled *C*. *dubliniensis* genome contains the given locus)_L/R (‘L’ and ‘R’ stand for ‘Left’ and ‘Right’ respectively indicating the locus is on which arm of the chromosome)_K(chromosome(s) in *C*. *dubliniensis* karyotype that contain the locus).
(TIF)

S18 Fig The *C*. *dubliniensis* agar invasion and embedded agar filamentation phenotypes are also observed when *CdTLO2* overexpression is driven by the *CaACT1* and *CdENO1* promoters in Wü284 and additional *C*. *dubliniensis* strain backgrounds. **(A)** Embedded agar filamentation (two left columns) and agar invasion (two right columns) phenotype analysis with one copy of HA-tagged *CdTLO2* overexpressed from the *CaACT1* promoter in a wild type (Wü284) *C*. *dubliniensis* strain (yLM348). Spontaneous transformants that possessed the ‘SW’ phenotype (yLM349) were also observed when using the *CaACT1* promoter. **(B)** Overexpression of *CdTLO2* in different *C*. *dubliniensis* backgrounds produces similar phenotypes. The *pNAT-ENO1* cassette described by Milne et al. (Yeast 2011;28:833–41.) was inserted upstream of Cd*TLO2* in each isolate. Transformants produced wrinkled colonies with filamentous fringes on YPD Medium at 30˚C. Strain CD36 yielded mixed smooth/wrinkled colonies at approximately 50/50 frequency. In embedded agar conditions (YPS medium at 25˚C) all strains exhibited enhanced filamentous growth following insertion of the *ENO1p* promoter. Again, CD36 produced a mixed colony morphology. Strain AV5 was the only wild-type strain capable of filamentous growth under these conditions (approximately 40% wrinkled), however this was greatly enhance following insertion of the *ENO1p* cassette (100% wrinkled).
(TIF)

S19 Fig Overexpression of *CaTLOα12*, *CdTLO1* or *HyNT1C* is unable to recapitulate the filamentation phenotypes resulting from *CdTLO2* overexpression. **(A)** Agar invasion phenotype in *CdTLO2* (yLM339)**,** *CdTLO2* ‘SW” (yLM343) *CdTLO1* (yLM337), *CaTLOα12* (yLM335) and *HyNT1C* (yLM341) overexpression in a wild type (Wü284) *C*. *dubliniensis* strain monitored by resistance to washing. (B) Filamentation in embedded agar phenotype in *CdTLO2* (yLM339)**,** *CdTLO2* ‘SW” (yLM343) *CdTLO1* (yLM337), *CaTLOα12* (yLM335) and *HyNT1C* (yLM341) overexpression in a wild type (Wü284) *C*. *dubliniensis* strain.
(TIF)

S20 Fig Overexpression of *CdTLO2* in *C*. *dubliniensis*, with the exception of the ‘SW’ strain, results in minor effects on stress related phenotypes previously observed to be affected in *tlo1Δ/Δ* and/or *med3Δ/Δ C*. *dubliniensis* strains. **(A)** Dilution series of strains over-expressing *TLO* genes in WT (Wü284 as the parental strain, yLM337 for *CdTLO1* over-expression, yLM339 for *CdTLO2*, yLM335 for *CaTLOα12* and yLM341 for *HyNT1C*) and *med3Δ/Δ* (yLM300 as the parental strain, yLM338 for overexpressing *CdTLO1*, yLM340 for *CdTLO2*, yLM336 for *CaTLOα12* and yLM342 for *HyNT1C*) *C*. *dubliniensis* backgrounds. A *tlo1Δ/Δ* (yLM123) *C*. *dubliniensis* strain and the ‘SW’ transformant derived from *CdTLO2-3HA*_*1X*_ transformation (yLM343) are also included in the comparison. Growth is tested on agar plates under the conditions listed above the each panel with the base conditions being YPD media at 30°C unless otherwise noted. *CdTLO2* over-expression SW strains showed slow growth upon exposure to high temperatures (41.5°C) and SDS. **(B)** When utilizing galactose as carbon source, colonies from the *CdTLO2* overexpression strains, particularly the ‘SW’ isolates, tend to undergo further enhanced filamentous growth.
(TIF)

S21 Fig Nuclear localization of endogenous CdMed3 protein in *C*. *dubliniensis* is dependent on CdTlo1. One endogenous copy of CdMed3 was GFP tagged at its C-terminus and its localization was observed in wild type (yLM359), *tlo1Δ/Δ* (yLM360) and *tloΔ/Δ* (yLM361) *C*. *dubliniensis* strains. Differential contrast (DIC) and fluorescence microscopy were used to visualize GFP localization, while Hoechst staining was used to stain the nuclei. All cells were grown in synthetic complete media overnight, diluted into the same media and grown for 5–6 hours before visualization.
(TIF)

S22 Fig The enhanced phenotype caused by nuclear localized *CdTLO2* is not due to multiple copies of the *NLS-GFP-TLO2-HA* over-expression cassette. Radar plot of *CdTLO2* copy number in strains containing Cd*TLO2-HA* over-expression cassettes with and without a nuclear localization sequence. Genomic DNA of two independent transformants of a *C*. *dubliniensis* strain over-expressing *TDH3* promoter-driven *NLS-GFP-TLO2-HA* (yLM367) was extracted from over-night YPD cultures and analyzed by qPCR to quantify the exogenous *TLO2* ORF and *SAT1* ORF copy number. The result is presented in comparison with the numbers obtained from *C*. *dubliniensis* strains over-expressing one copy (1X) and two copies (2X) of *TDH3* promoter-driven *TLO2-3HA* (yLM339 and yLM344 respectively).
(TIF)

S23 Fig Overexpression of *CdTLO2*, without an HA-tag, recapitulates the filamentation phenotypes resulting from the HA-tagged *CdTLO2* overexpression. **(A)** Embedded agar filamentation (two left columns) and agar invasion (three right columns) phenotype analysis with two copies of *CdTLO2* (yLM346), or one copy of *NLS-GFP-TLO2* (yLM369) overexpressed from a *TDH3* promoter in wild type (Wü284) *C*. *dubliniensis* strain. ‘SW’ colonies (yLM347), with multiple phenotypes (a & b), also occurred among the transformants with the CdTLO2 without the HA tag. **(B)** *NLS-GFP-TLO2* overexpression strain (yLM369) grown in liquid media and visualized by differential contrast (DIC) and fluorescence microscopy were used to detect GFP localization, while Hoechst staining was used to stain the nuclei. All cells were grown in synthetic complete media overnight, diluted into the same media and grown for 5–6 hours before visualization.
(TIF)

S24 Fig Nuclear localization of the over-expressed transcriptional activation domain of CdTlo2 in a *tlo1Δ/Δ C*. *dubliniensis* strain, by fusion of a NLS to its N-terminus, facilitates embedded agar and agar invasion phenotypes. **(A)** *TLO* constructs, which were N-terminally fused with NLS-GFP sequence and C-terminally HA-tagged, were over-expressed from a *TDH3* promoter in a *tlo1Δ/Δ C*. *dubliniensis* strain (yLM370 for over-expression of *NLS-GFP-3HA*, yLM371 for *CaTLOα12*, yLM373 for *CdTLO1* and yLM375 for *CdTLO2*). The *CaTLOα12TAD* (yLM372) strain contained residues 164–252 of *CaTLOα12*. The *CdTLO1TAD* (yLM374) strain contained residues 199–320 of *CdTLO1*. The *CdTLO2TAD* strain (yLM376) contained residues 255–367 of *CdTLO2*. Differential contrast (DIC) and fluorescence microscopy were used to visualize GFP localization, while Hoechst staining was used to stain the nuclei. All cells were grown in synthetic complete media overnight, diluted into the same media and grown for 5–6 hours before visualization. **(B)** Embedded agar filamentation (two left columns) and agar invasion (three right columns) phenotype analysis with NLS-GFP *C*. *dubliniensis* strains described in A.
(TIF)

S25 Fig ‘SW’ colonies do not exhibit a greater degree of CdTlo2 nuclear localization than ‘smooth’ colonies, when a *GFP-CdTLO2* construct is overexpressed in *C*. *dubliniensis*. *CdTLO2*, which was N-terminally tagged with a GFP sequence and C-terminally HA-tagged, was overexpressed from a *TDH3* promoter in a wild type (Wü284) *C*. *dubliniensis* strain, and produced transformants of both the ‘smooth’ (yLM384) and ‘SW’ (yLM385) colony phenotypes. (Left) Differential contrast (DIC) and fluorescence microscopy were used to visualize GFP localization, while Hoechst staining was used to stain the nuclei. All cells were grown in synthetic complete media overnight, diluted into the same media and grown for 5–6 hours before visualization. (Right) Embedded agar filamentation and agar invasion phenotype analysis with the *GFP-CdTLO2* ‘smooth’ and ‘SW’ *C*. *dubliniensis* strains.
(TIF)

S1 Text Supplemental Material and Methods. (PDF)

S1 Table Growth conditions tested for phenotypes caused by overexpression of *CaTLOα12*(yLM331), *CdTLO1* (yLM332) and *HyNT1C* (yLM333). (PDF)

S2 Table List of *C*. *dubliniensis* strains used in this study. (PDF)

S3 Table List of *C*. *albicans* strains used in this study. (PDF)

S4 Table List of plasmids used in this study. (PDF)

S5 Table List of primers used in this study. (PDF)

S6 Table Construction details for Series A and Series B Chimeric Genes. (PDF)

S7 Table Construction details for ‘non-series A/B’ chimeric genes. (PDF)
