# Supplementary figures and images for: Amplification of TLO Mediator Subunit Genes Facilitate Filamentous Growth in Candida Spp
Source: PLoS Genet. 2016 Oct 14;12(10):e1006373. doi: 10.1371/journal.pgen.1006373 (PMC5065183; doi:10.1371/journal.pgen.1006373)

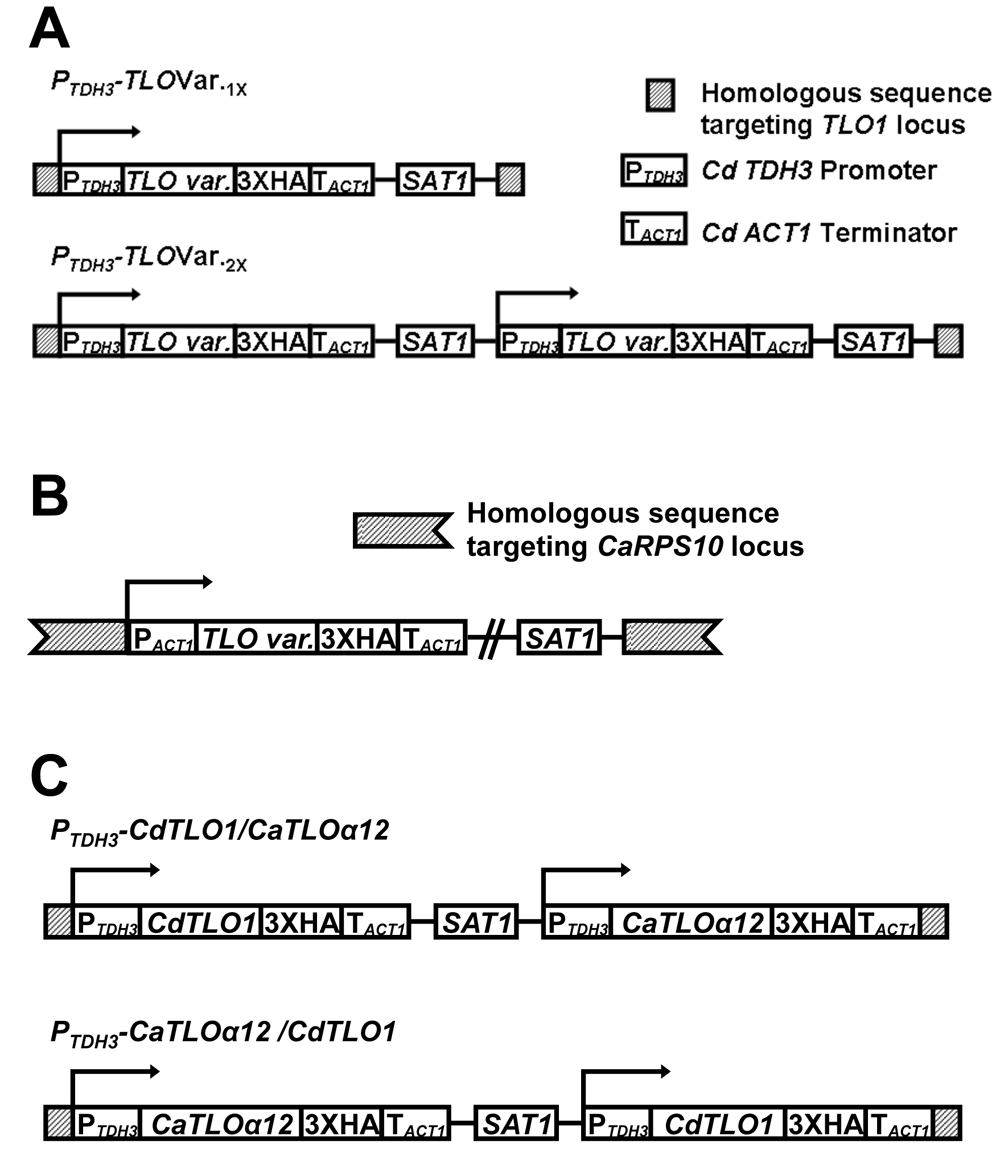

Supplement: S1 Fig — (A) Constructs for integrating one or two copies of a TLO gene into the TLO1 locus in C. dubliniensis. The constructs bearing a C-terminal 3XHA tag are shown here. In certain experiments, un-tagged or 6His3Flag tagged constructs were used to determine the phenotypes of non-tagged TLO variants or to facilitate the affinity purification of a Tlo protein respectively. (B) Construct for integrating a TLO gene into the RPS10 locus in C. albicans. (C) Constructs for integrating a combination of CdTLO1 and CaTLOα12 genes into the TLO1 locus in C. dubliniensis. (TIF) [file pgen.1006373.s001.tif]

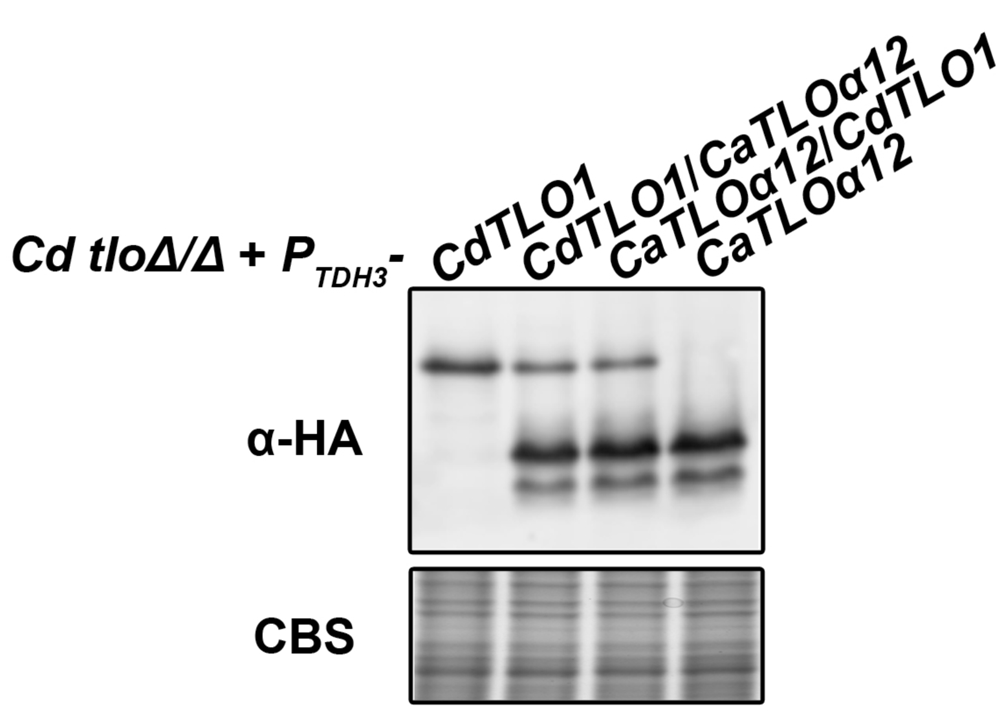

Supplement: S2 Fig — Immunoblot showing that TDH3 promoter driven co-expression of HA-tagged CdTLO1 and CaTLOα12 in a tlo null C. dubliniensis strain (yLM306 and yLM307) leads to a decrease in the steady-state level of CdTlo1p when compared to the CdTlo1p level in a strain solely over-expressing CdTlo1 (yLM302). Coomassie blue staining (CBS) was used as a loading control. (TIF) [file pgen.1006373.s002.tif]

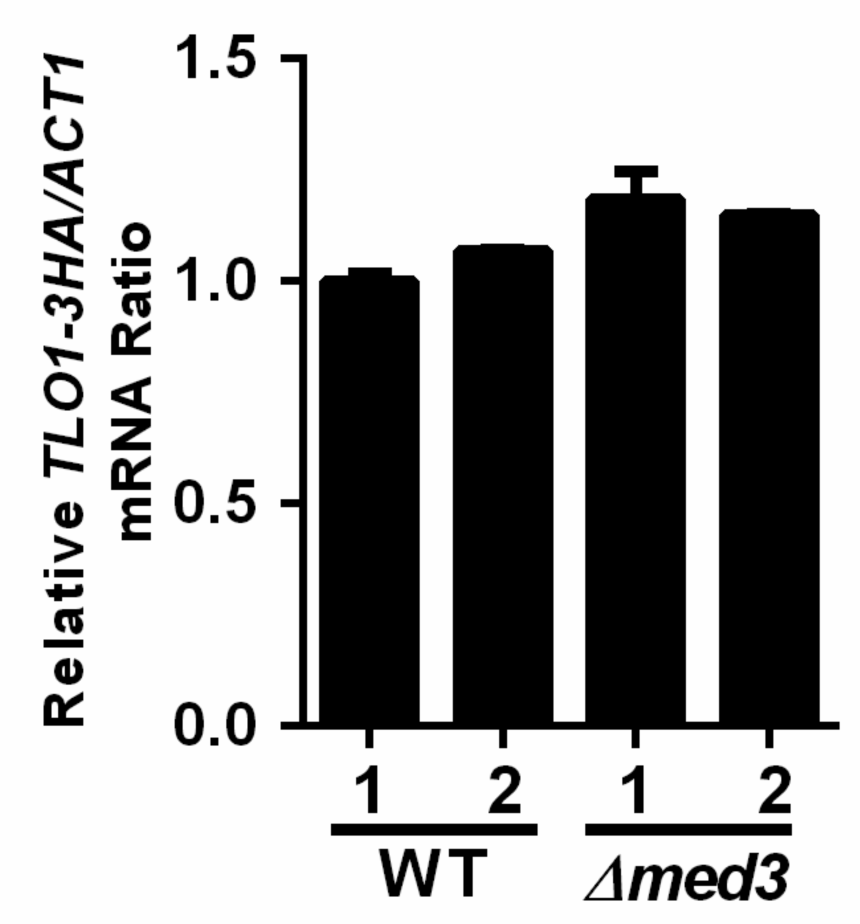

Supplement: S3 Fig — Liquid culture of two independent colonies (‘1’ and ‘2’) from TLO1 C-terminal HA tagged wild type (yLM301) and med3 null (yLM308) C. dubliniensis strains were grown and the RNA extracted. The steady state ratio of TLO1 mRNA levels to ACT1 mRNA was determined by RT-qPCR and normalized by setting the measurement of the first colony mRNA to one. The error bars represent the technical deviation of two sets of qPCR measurements on a given sample. (TIF) [file pgen.1006373.s003.tif]

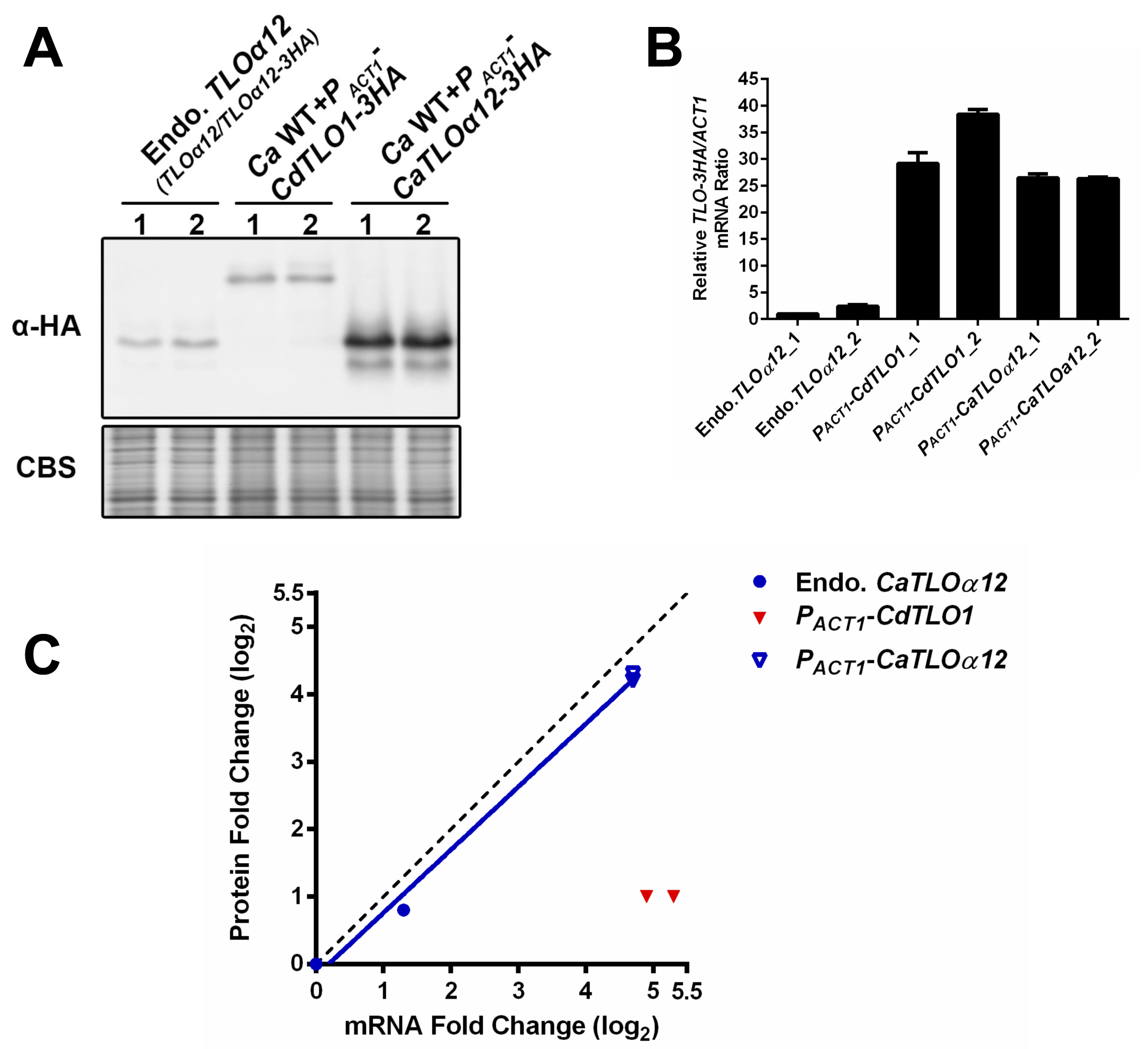

Supplement: S4 Fig — (A) Immunoblot showing that HA-tagged CdTlo1p (yLM390) levels are lower than CaTloα12p (yLM389) levels when expressed from the same strong promoter (pACT1) in C. albicans. Two independent transformants (‘1’ and ‘2’) were tested and compared with two independent C. albicans transformants, each with one endogenous TLOα12 tagged by the same 3XHA tag (yLM388). Coomassie blue staining (CBS) was used as a loading control. (B) RT-qPCR analysis showing that CdTLO1 mRNA levels in yLM390 are comparable to CaTLOα12 levels in yLM389 when expressed from the same strong promoter (pACT1) in C. albicans. Two independent transformants (‘1’ and ‘2’) were tested. The steady state mRNA levels were calculated as a ratio to ACT1 mRNA and normalized by setting the measurement of one of the endogenous CaTLOα12 tagged strains (yLM388) to one. The error bars represent the technical deviation of two sets of qPCR measurements on a given sample.(C) Plot of fold-change of overexpressed CdTLO1 and CaTLOα12 mRNA and protein (from A. and B.) compared to the endogenous levels of CaTLOα12. The dashed line represents an idealized slope of 1 in which the fold-change in protein is equal to the fold-change in mRNA. (TIF) [file pgen.1006373.s004.tif]

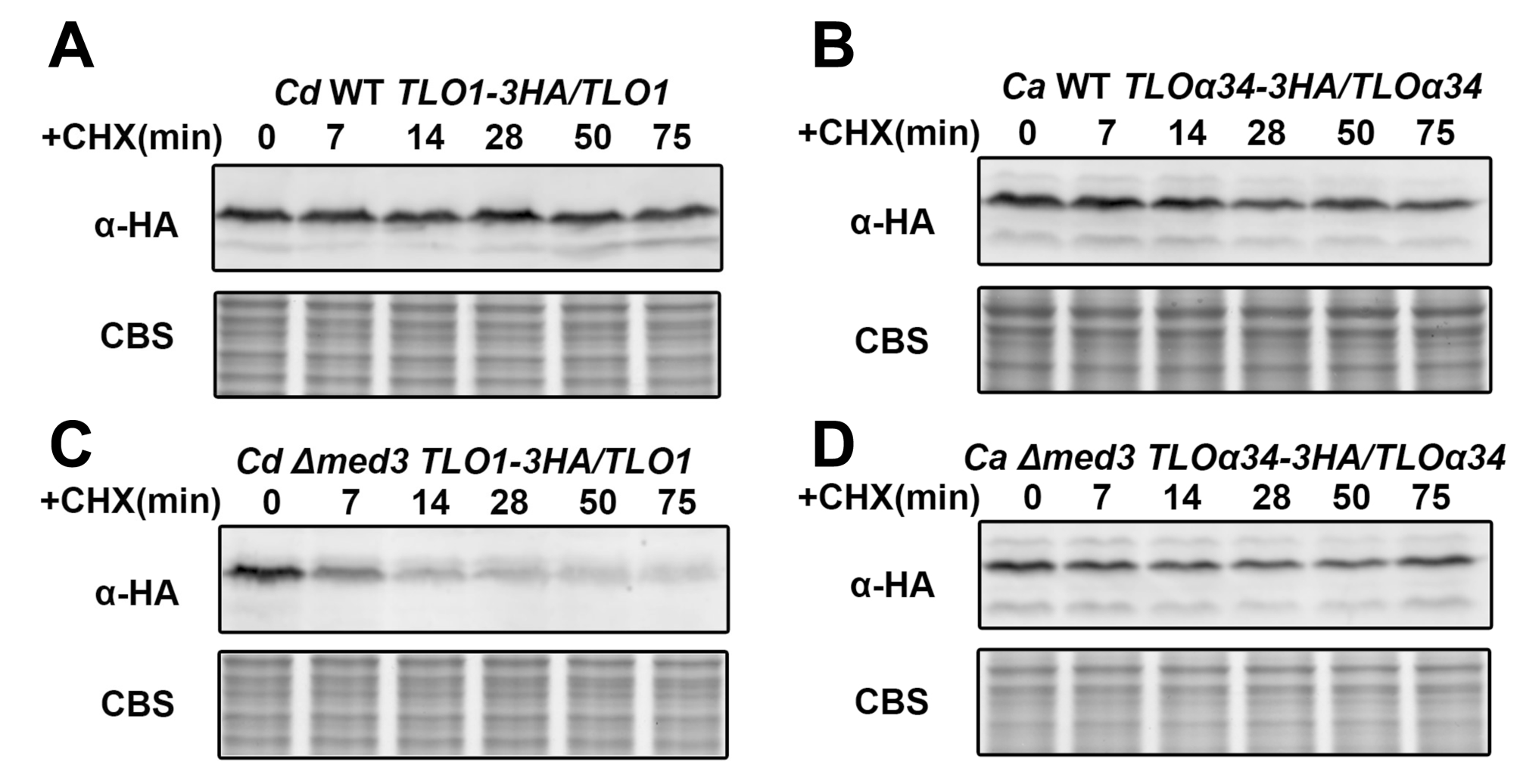

Supplement: S5 Fig — (A) Immunoblot of endogenous HA-tagged CdTlo1p in a wild type C. dubliniensis strain (yLM301) after treatment with cycloheximide (CHX). Coomassie blue staining (CBS) was used as a loading control. (B) Immunoblot of endogenous HA-tagged CaTloα34 in a wild type C. albicans strain (yLM391) after treatment with cycloheximide. Coomassie blue staining was used as a loading control. (C) Immunoblot of endogenous HA-tagged CdTlo1p in a med3Δ/Δ C. dubliniensis strain (yLM308) after treatment with cycloheximide. Coomassie blue staining was used as a loading control. (D) Immunoblot of endogenous HA-tagged CaTloα34 in med3Δ/Δ C. albicans strain (yLM392) after treatment with cycloheximide. Coomassie blue staining was used as a loading control. (TIF) [file pgen.1006373.s005.tif]

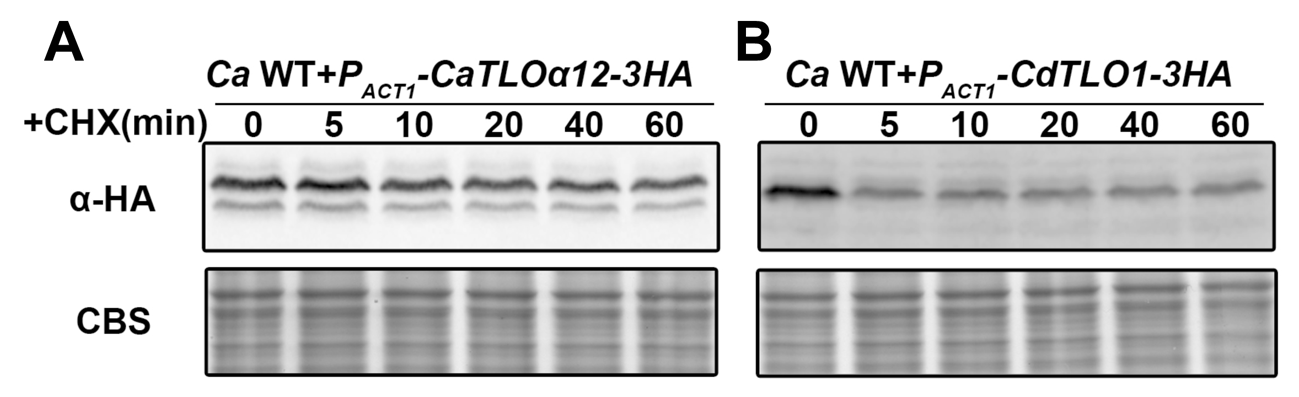

Supplement: S6 Fig — (A) Immunoblot of overexpressed HA-tagged CaTloα12p in a wild type C. albicans strain (yLM393) after treatment with cycloheximide (CHX). Coomassie blue staining (CBS) was used as a loading control. (B) Immunoblot of overexpressed HA-tagged CdTlo1p in a wild type C. albicans strain (yLM394) after treatment with cycloheximide. Coomassie blue staining was used as a loading control. (TIF) [file pgen.1006373.s006.tif]

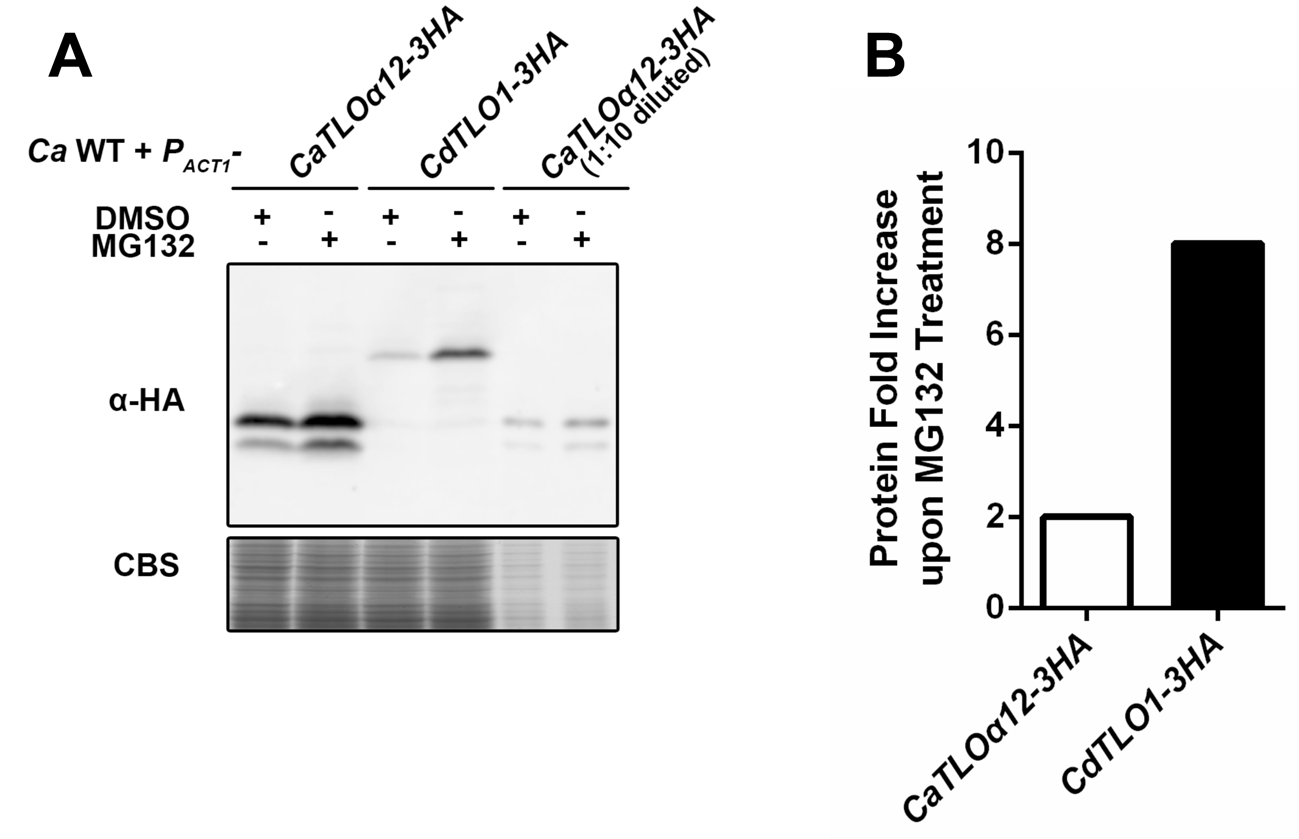

Supplement: S7 Fig — (A) Immunoblot of overexpressed HA-tagged CaTloα12p and CdTlo1p in a wild type C. albicans strain (yLM389 and yLM390 respectively) in the presence and absence of the proteasome inhibitor MG132. Coomassie blue staining (CBS) was used as a loading control. (B) Plot of the increase in CdTloα12p and CdTlo1p upon treatment with MG132 quantified from the blot in part A. (TIF) [file pgen.1006373.s007.tif]

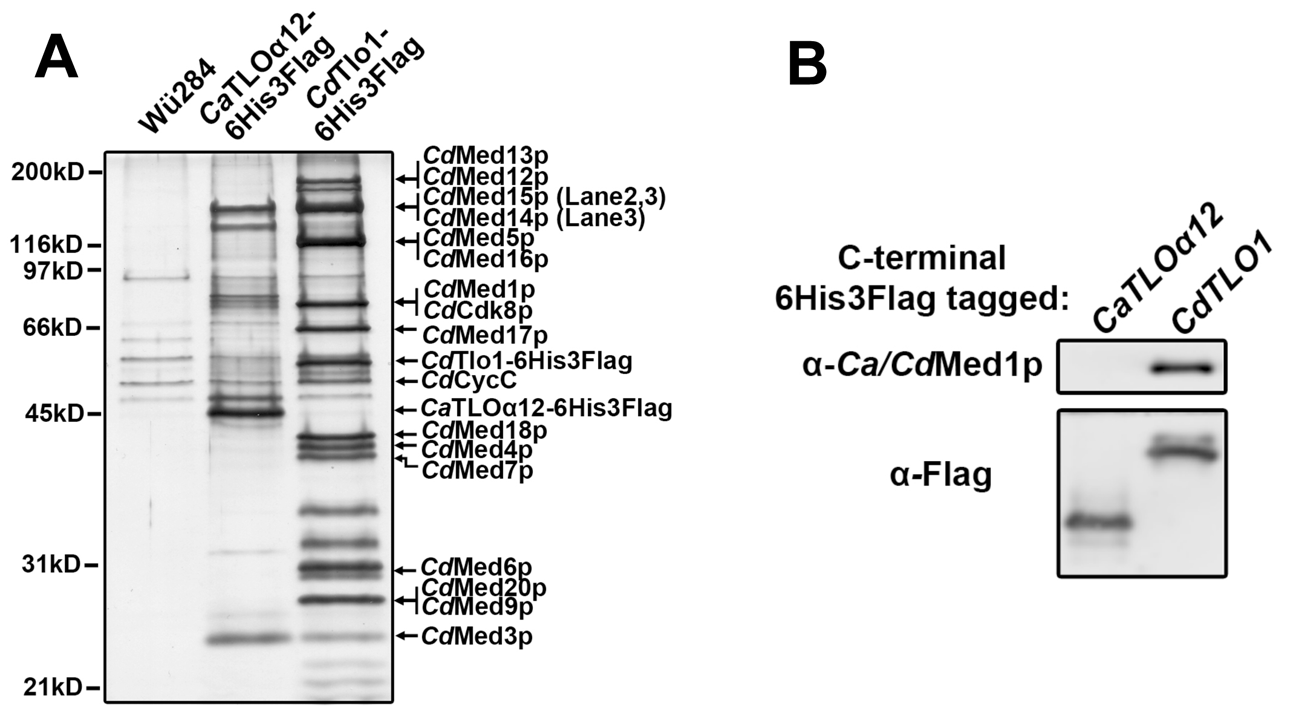

Supplement: S8 Fig — (A) Silver staining SDS PAGE analysis of affinity purified protein form an untagged (Wü284), 6His-3Flag tagged CaTloα12 (yLM309) and 6His-3Flag tagged CdTlo1 C. dubliniensis strain (yLM415). (B) Immunoblot analysis of affinity purified 6His-3Flag tagged CaTloα12 and 6His-3Flag tagged CdTlo1 from C. dubliniensis. An anti-Med1 antibody is used to show that 6His-3Flag tagged CdTlo1p is able to pull down the Middle module subunit, Med1, but 6His-3Flag tagged CaTloα12p could not in the affinity purified sample in A. (TIF) [file pgen.1006373.s008.tif]

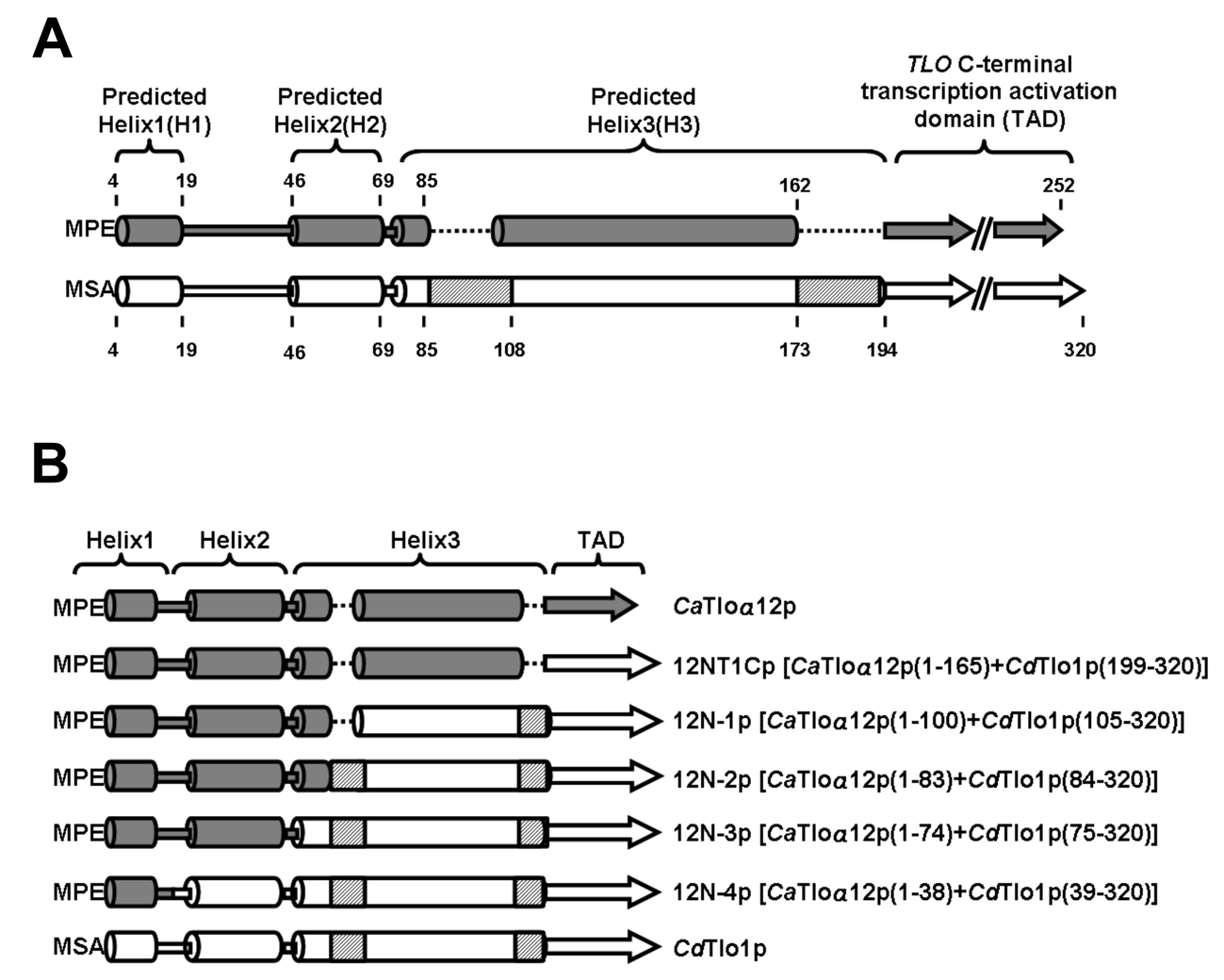

Supplement: S9 Fig — (A) Detailed schematic of predicted secondary structure of Tloα12 and CdTlo1. (B) Detailed schematic of chimeric proteins containing various Tloα12 N-terminal and CdTlo1 C-terminal sequences. In each panel the first three amino-acids (MSA or MPE) are denoted at the beginning of each construct. The segments shaded light grey represent regions of CdTlo1p ‘Helix 3’ that do not have clear homologous sequence in CaTloα12 ‘Helix 3.’ (TIF) [file pgen.1006373.s009.tif]

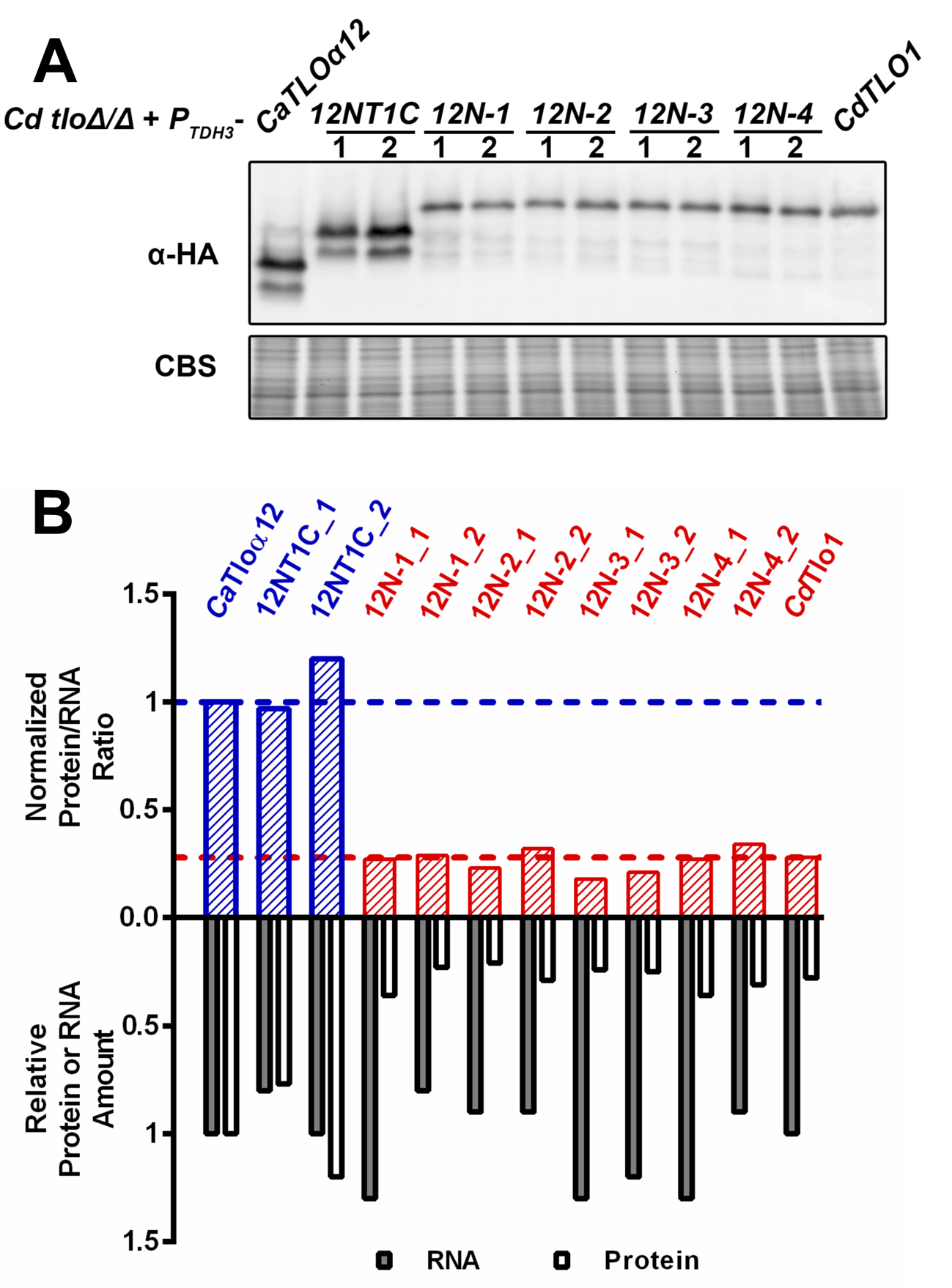

Supplement: S10 Fig — (A) Immunoblot showing over-expression of HA-tagged CaTLOα12, CdTLO1, and CaTLOα12/CdTLO1 chimeras from the TDH3 promoter in a C. dubliniensis tloΔ/Δ strain. Strains used to generate the data are yLM303(CaTLOα12), yLM310(12NT1C), yLM311(12N-1), yLM312(12N-2), yLM313(12N-3), yLM314(12N-4) and yLM302(CdTLO1). Two independent transformants (‘1’ and ‘2’) of each strain were tested. Coomassie blue staining (CBS) was used as a loading control. (B) Bottom half contains plot showing relative expression of HA-tagged CaTLOα12, CdTLO1, and CaTLOα12/CdTLO1 chimeric proteins (from immunoblot in A.) and mRNA (from RT-qPCR) normalized to CaTLOα12, and top half contains shows a plot of protein to mRNA ratios derived from the corresponding data on the bottom half of the graph. (TIF) [file pgen.1006373.s010.tif]

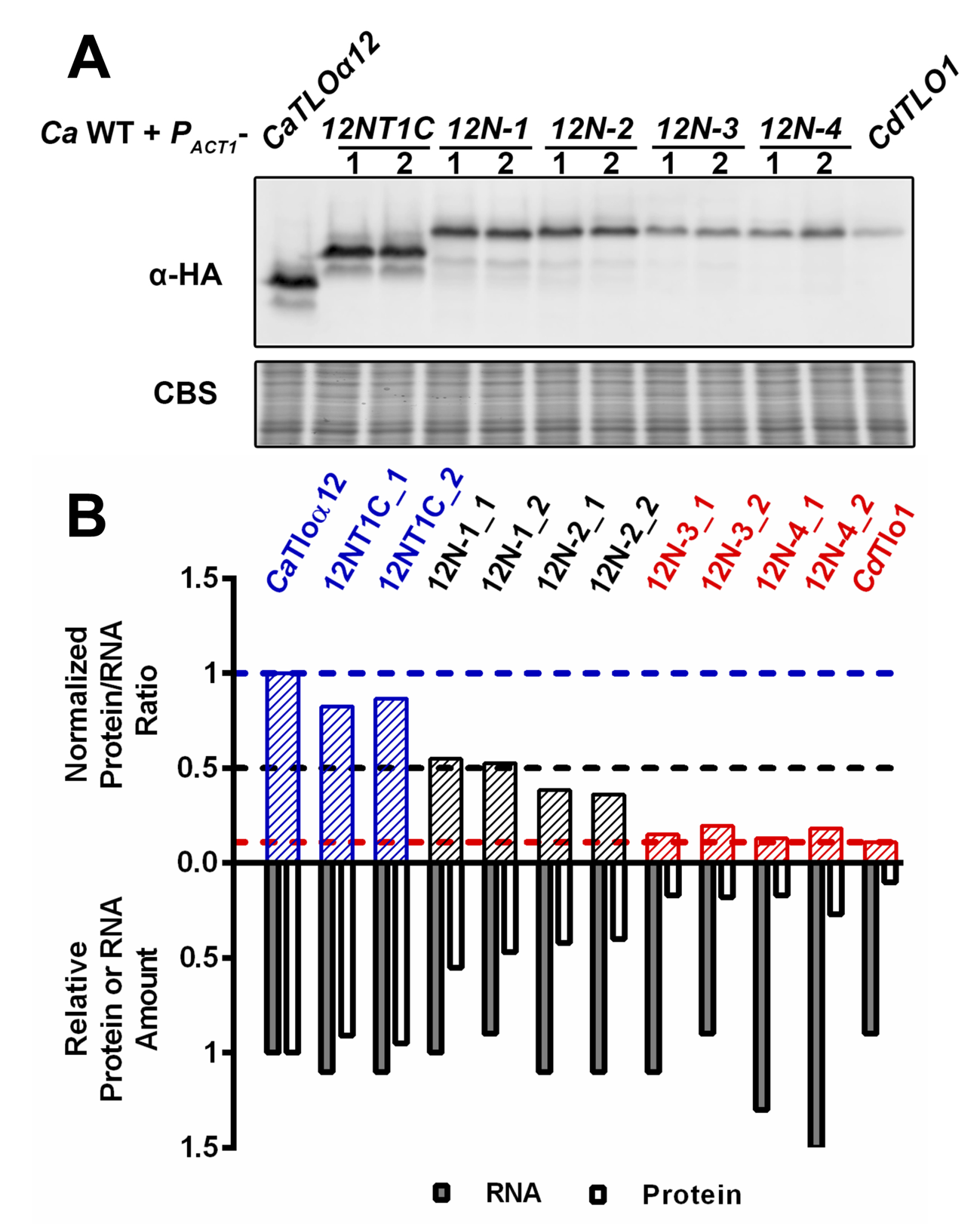

Supplement: S11 Fig — (A) Immunoblot showing over-expression of HA-tagged CaTLOα12, CdTLO1, and CaTLOα12/CdTLO1 chimeras from the TDH3 promoter in a wild type C. albicans strain. Strains used to generate the data are yLM389(CaTLOα12), yLM395(12NT1C), yLM396(12N-1), yLM397(12N-2), yLM398(12N-3), yLM399(12N-4) and yLM390(CdTLO1). Two independent transformants (‘1’ and ‘2’) were tested for each strain. Coomassie blue staining (CBS) was used as a loading control. (B) Bottom half contains plot showing relative expression of HA-tagged CaTLOα12, CdTLO1, and CaTLOα12/CdTLO1 chimeric proteins (from immunoblot in A.) and mRNA (from RT-qPCR) normalized to CaTLOα12, and top half contains shows a plot of protein to mRNA ratios derived from the corresponding data on the bottom half of the graph. (TIF) [file pgen.1006373.s011.tif]

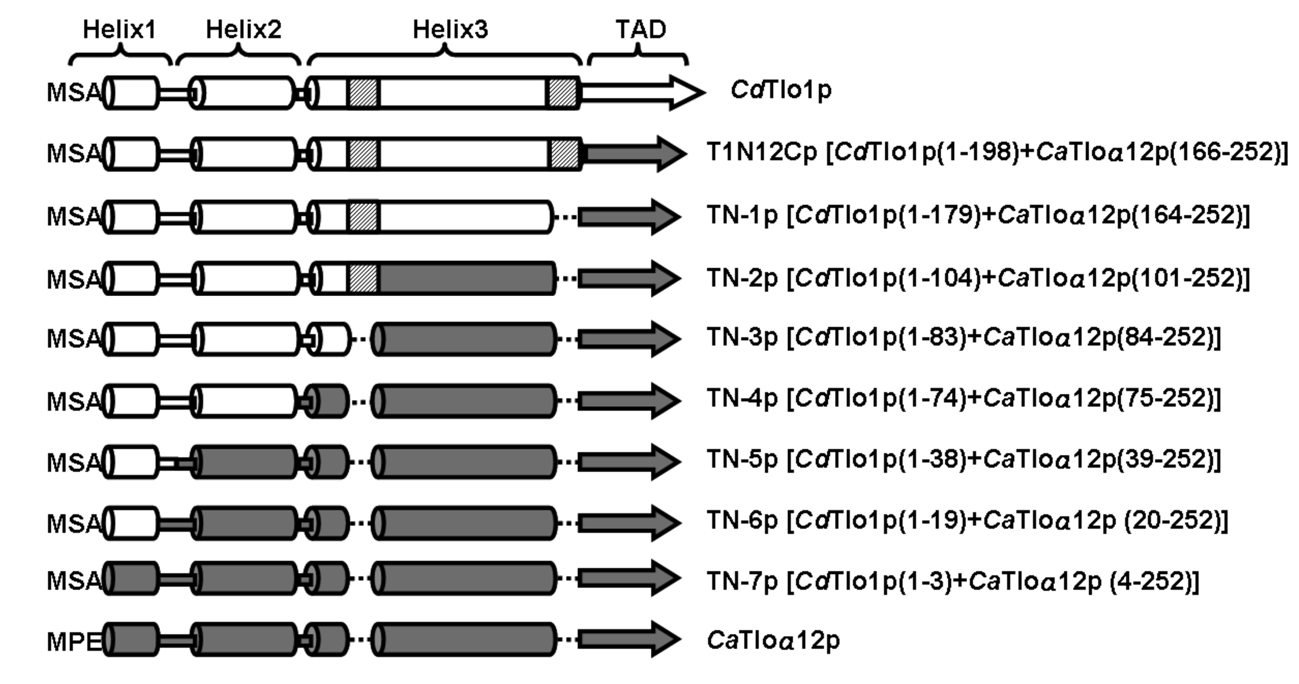

Supplement: S12 Fig — Detailed schematic of chimeric proteins containing various CaTloα12 C-terminal and CdTlo1 N-terminal sequences. The first three amino-acids (MSA or MPE) are denoted at the beginning of each construct. The segments shaded light grey represent regions of CdTlo1p ‘Helix 3’ that do not have clear homologous sequence in CaTloα12 ‘Helix 3.’ (TIF) [file pgen.1006373.s012.tif]

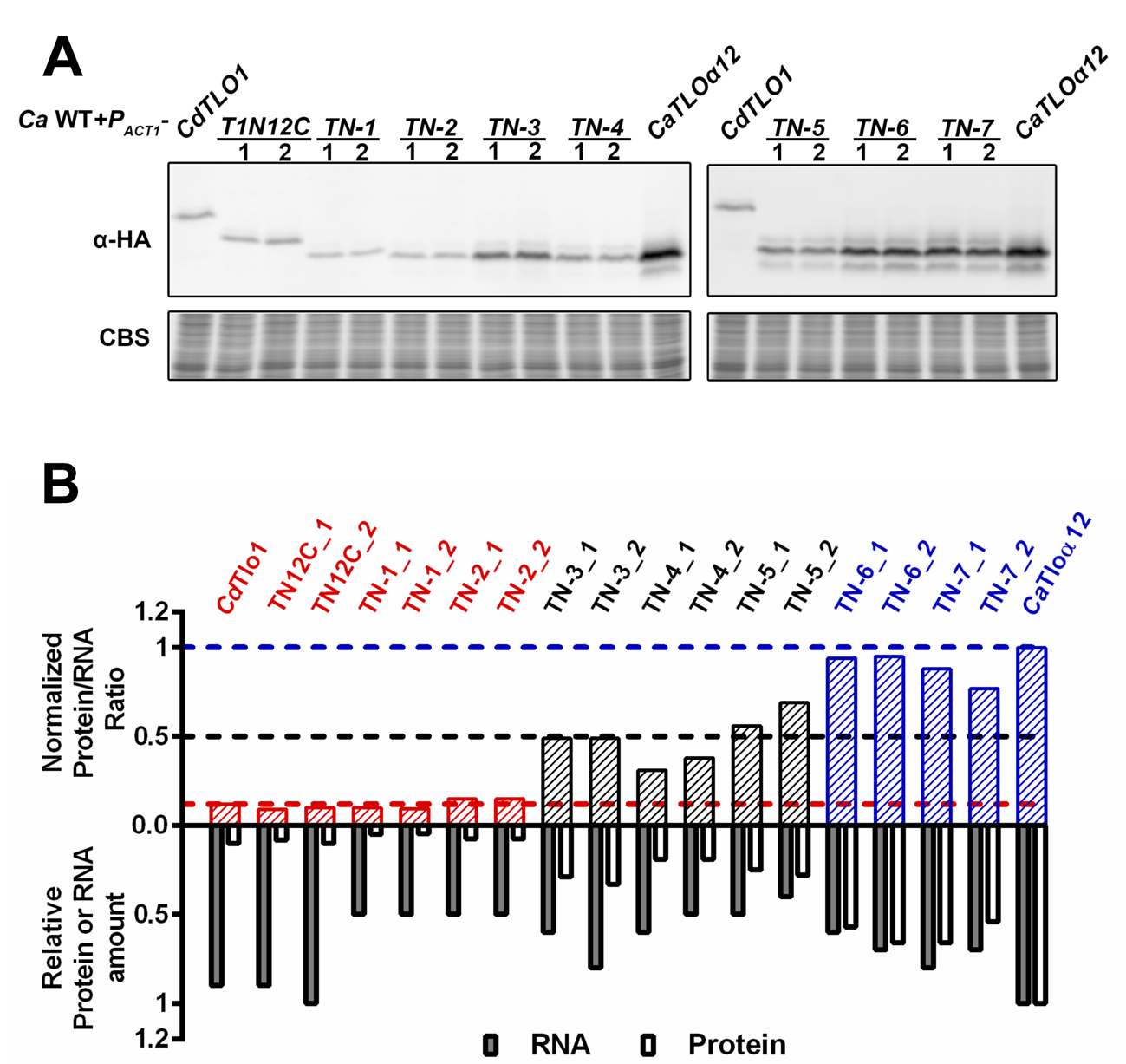

Supplement: S13 Fig — (A) Immunoblot showing over-expression of HA-tagged CaTLOα12, CdTLO1, and CdTLO1/CaTLOα12 chimeras from the TDH3 promoter in a wild type C. albicans strain. Strains used to generate the data are yLM390(CdTLO1), yLM400(T1N12C), yLM401(TN-1), yLM402(TN-2), yLM403(TN-3), yLM404(TN-4), yLM405(TN-5), yLM406(TN-6), yLM407(TN-7) and yLM389(CaTLOα12). Two independent transformants (‘1’ and ‘2’) were tested. Coomassie blue staining (CBS) was used as a loading control. (B) Bottom half contains plot showing relative expression of HA-tagged CaTLOα12, CdTLO1, and CdTLO1/CaTLOα12 chimeric proteins (from immunoblot in A.) and mRNA (from RT-qPCR) normalized to CaTLOα12, and top half contains shows a plot of protein to mRNA ratios derived from the corresponding data on the bottom half of the graph. (TIF) [file pgen.1006373.s013.tif]

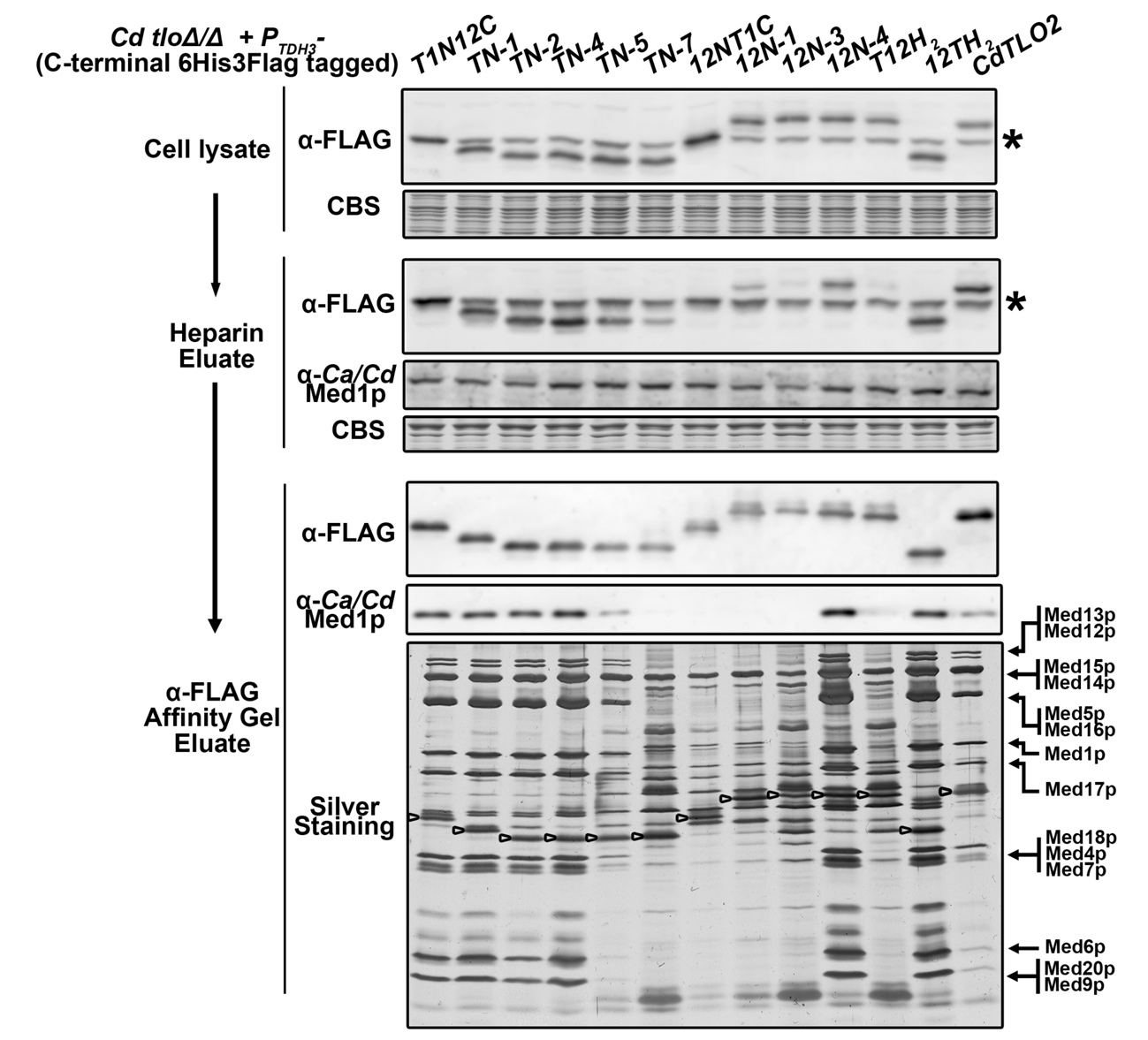

Supplement: S14 Fig — Immunoblot analysis of cell lysate, heparin purified, and Flag-purified samples derived from a C. dubliniensis tloΔ/Δ strain over expressing 6His-3Flag tagged CdTlo2 (yLM327) and CaTloα12/CdTlo1 chimeric proteins including T1N12C(yLM315), TN-1(yLM316), TN-2(yLM317), TN-4(yLM318), TN-5(yLM319), TN-7(yLM320), 12NT1C(yLM321), 12N-1(yLM322), 12N-3(yLM323), 12N-4(yLM324), T12H2(yLM325) and 12NTH2(yLM326). There is a contaminant (*) that cross reacts with the α-Flag antibody in the cell lysate and heparin stages of the isolation. An α-Med1 antibody is used to track the presence of intact Mediator. Coomassie blue staining (CBS) was used as a loading control. The final Flag-purified 6His-3Flag tagged CaTloα12/CdTlo1 chimeric proteins are analyzed by silver stain analysis to reveal the presence of Mediator subunits from the Middle, Head and Cdk8 modules. Arrow heads point to a silver stained band representing the chimeric protein. (TIF) [file pgen.1006373.s014.tif]

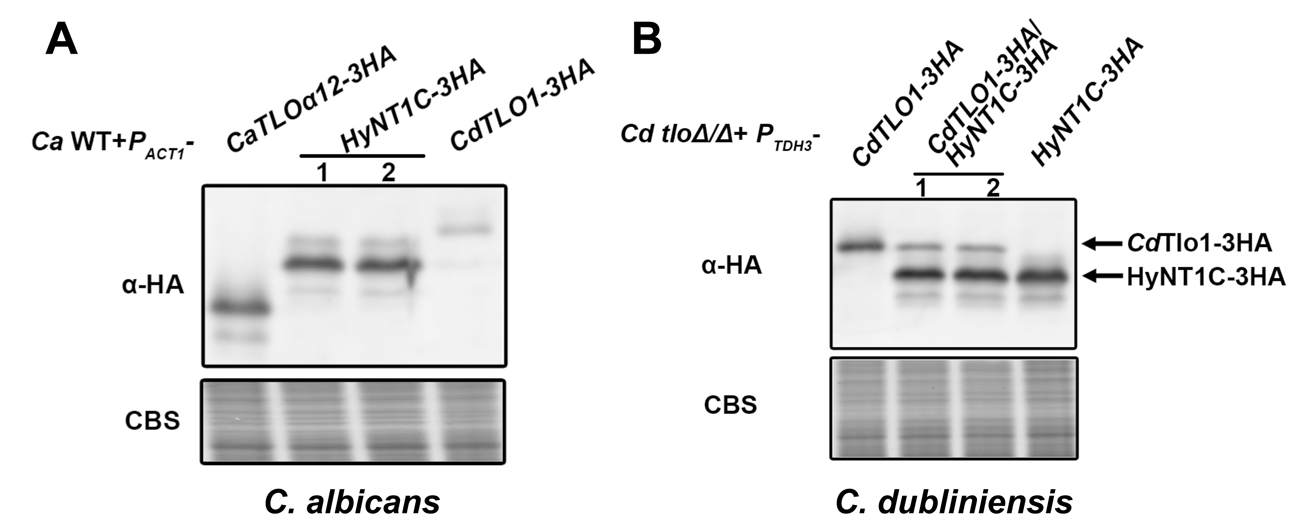

Supplement: S15 Fig — (A) Immunoblot showing over-expressed HA-tagged HyNT1C in C. albicans (yLM408) led to accumulation of protein equivalent to overexpression of CaTLOα12 (yLM389) and exceeding CdTLO1(yLM390). Two independent transformants (‘1’ and ‘2’) were tested. Coomassie blue staining (CBS) was used as a loading control. (B) Immunoblot showing that TDH3 promoter driven co-expression of HA-tagged HyNT1C and CdTLO1 in a tlo null C. dubliniensis strain (yLM330), leads to a decrease in the steady-state level of CdTlo1p when compared to CdTlo1p levels in a strain (yLM302) solely over-expressing CdTLO1. Two independent transformants (‘1’ and ‘2’) were tested. Coomassie blue staining (CBS) was used as a loading control. (TIF) [file pgen.1006373.s015.tif]

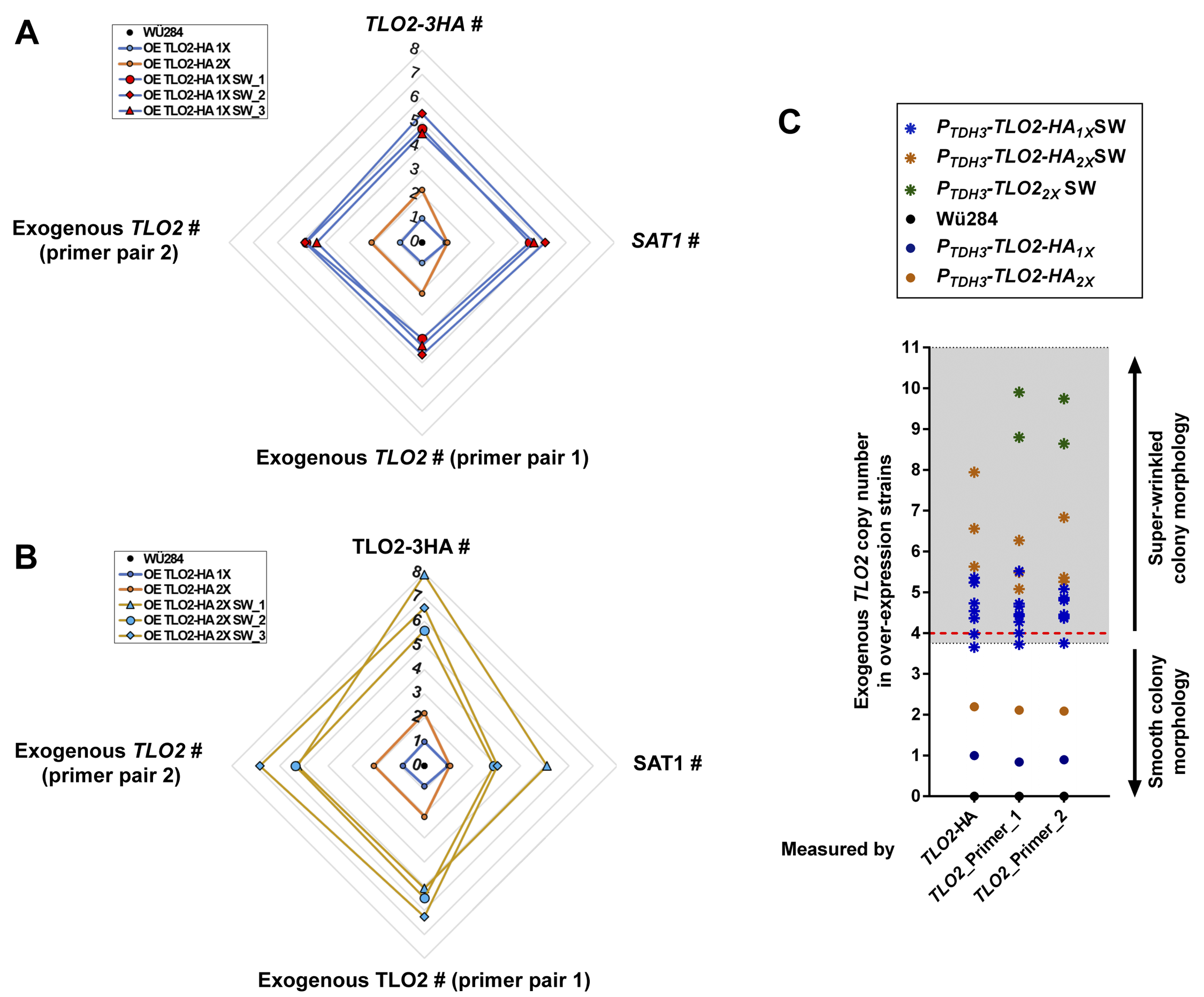

Supplement: S16 Fig — (A) Radar plot shows extra copies of TLO2-HA ORF and SAT1 ORF are present in 3 independent ‘SW’ (yLM343) isolates compared with an isolate of their smooth (yLM339) counterpart when wild type C. dubliniensis (Wü284) is transformed with the CdTLO2-HA1x cassette (one copy of TLO2-HA driven by a TDH3 promoter). Genomic DNA extracted from an overnight YPD culture of the indicated strains was quantified for exogenous TLO2 and SAT1 copy number by qPCR using primer pairs annealing to different regions within the TLO2 ORF. SAT1# is the number of copies of the SAT1 cassette; TLO2-3HA# is the number of HA-tagged CdTLO2 copies measured using one primer to the HA-tag coding sequence and one primer to the CdTLO2 coding sequence; and exogenous TLO2# is the number of copies of CdTLO2 using one of two primer pairs (‘1’ and ‘2’) minus the two endogenous CdTLO2 copies. The CdTLO2-HA2x (two copies of TLO2-HA each driven by a TDH3 promoter) smooth transformant (yLM344) and the parental wild type C. dubliniensis (Wü284) are also included as reference. (B) Radar plot shows extra copies of TLO2-HA ORF and SAT1 ORF are present in 3 independent ‘SW’ (yLM345) isolates compared with an isolate of their smooth (yLM344) counterpart when wild type C. dubliniensis (Wü284) is transformed with the CdTLO2-HA2x cassette (two copies of TLO2-HA driven by a TDH3 promoter with a single SAT1 cassette). Genomic DNA extracted from an overnight YPD culture of the indicated strains was quantified for exogenous TLO2 and SAT1 copy number by qPCR using primer pairs annealing to different regions within the TLO2 ORF. SAT1# is the number of copies of the SAT1 cassette; TLO2-3HA# is the number of HA-tagged CdTLO2 copies measured using one primer to the HA-tag coding sequence and one primer to the CdTLO2 coding sequence; and exogenous TLO2# is the number of copies of CdTLO2 using one of two primer pairs (‘1’ and ‘2’) minus the two endogenous CdTLO2 copies. The CdTLO2-HA2x (one copy of TLO2-HA driven by a TDH3 promote [file pgen.1006373.s016.tif]

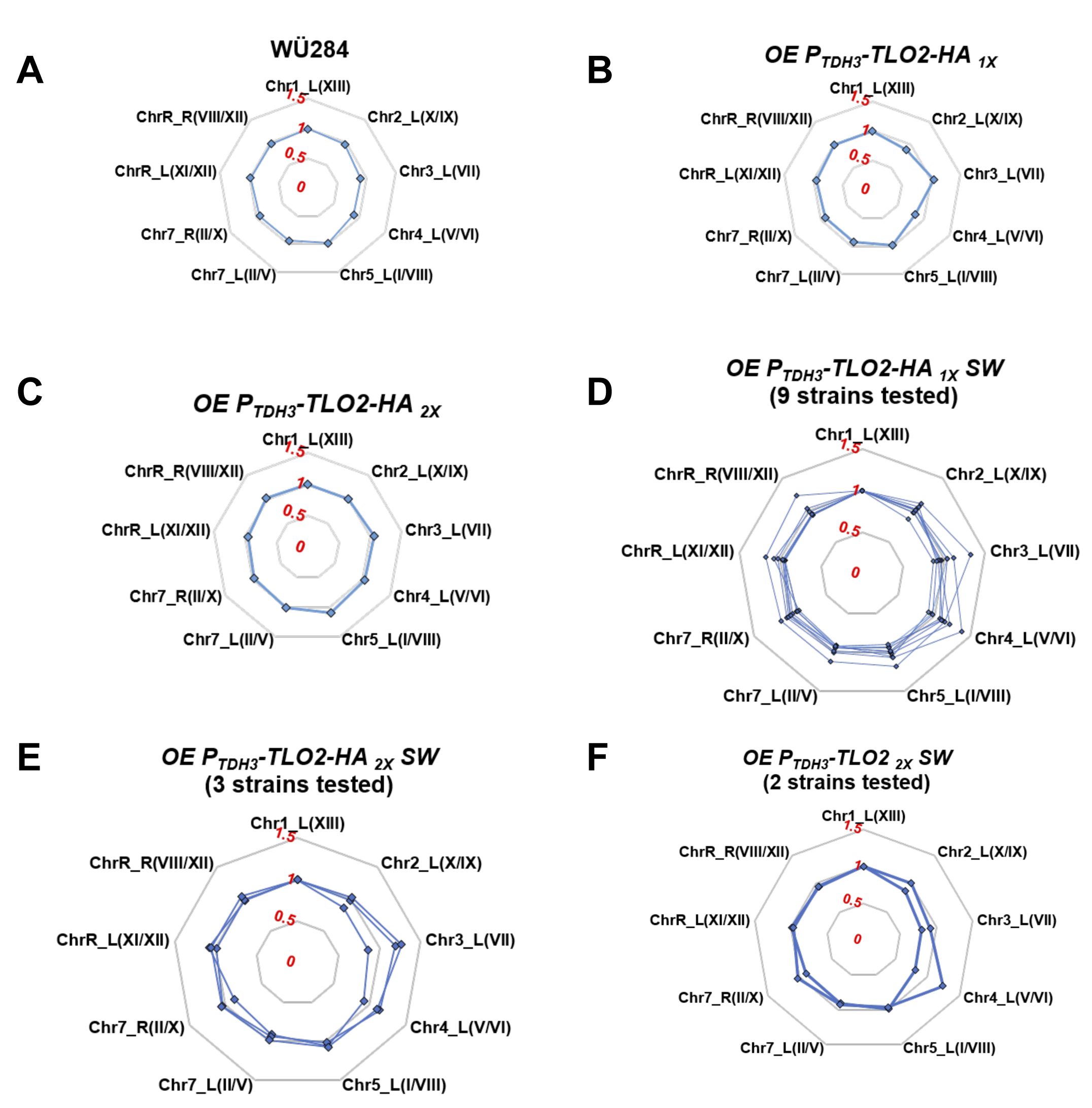

Supplement: S17 Fig — Radar plots showing relative ploidy analysis of: (A) the parental wild type Wü284 C. dubliniensis strain; (B) a C. dubliniensis strain over-expressing one copy (1X) of HA-tagged TLO2 driven by a TDH3 promoter with smooth colony morphology (yLM339); (C) a C. dubliniensis strain over-expressing two copies (2X) of HA-tagged TLO2 each driven by a TDH3 promoter with smooth colony morphology (yLM344); (D) nine independent transformants with ‘super-wrinkled’ morphology obtained by transforming Wü284 with the TLO2-HA1X cassettes (yLM343); (E) three independent transformants with ‘super-wrinkled’ morphology obtained by transforming Wü284 with the TLO2-HA2X cassettes (yLM345); and (F) two independent transformants with ‘super-wrinkled’ morphology obtained by transforming Wü284 with a DNA cassette containing two copies of TDH3 promoter-driven non-tagged TLO2 (yLM347). A value of ‘1’ indicates a given strain has kept the same copy number at a tested chromosomal locus compared to the parental Wü284 strain, while ‘0.5’ and ‘1.5’ respectively indicate missing and acquiring one copy of a given locus during strain construction. Nine chromosomal loci were tested for each strain and annotated as Chr(Chromosome)_’# (chromosome in the assembled C. dubliniensis genome contains the given locus)_L/R (‘L’ and ‘R’ stand for ‘Left’ and ‘Right’ respectively indicating the locus is on which arm of the chromosome)_K(chromosome(s) in C. dubliniensis karyotype that contain the locus). (TIF) [file pgen.1006373.s017.tif]

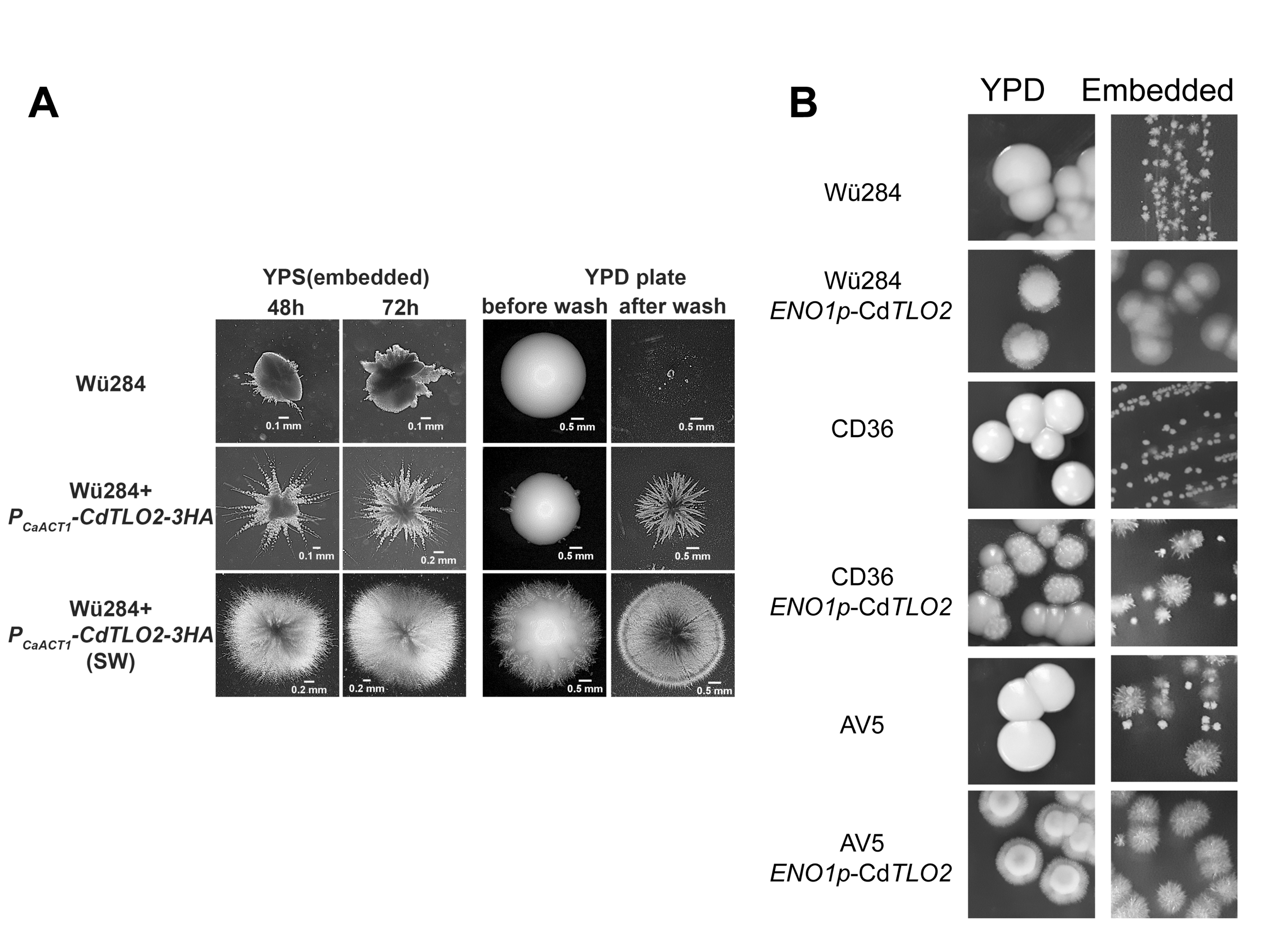

Supplement: S18 Fig — (A) Embedded agar filamentation (two left columns) and agar invasion (two right columns) phenotype analysis with one copy of HA-tagged CdTLO2 overexpressed from the CaACT1 promoter in a wild type (Wü284) C. dubliniensis strain (yLM348). Spontaneous transformants that possessed the ‘SW’ phenotype (yLM349) were also observed when using the CaACT1 promoter. (B) Overexpression of CdTLO2 in different C. dubliniensis backgrounds produces similar phenotypes. The pNAT-ENO1 cassette described by Milne et al. (Yeast 2011;28:833–41.) was inserted upstream of CdTLO2 in each isolate. Transformants produced wrinkled colonies with filamentous fringes on YPD Medium at 30˚C. Strain CD36 yielded mixed smooth/wrinkled colonies at approximately 50/50 frequency. In embedded agar conditions (YPS medium at 25˚C) all strains exhibited enhanced filamentous growth following insertion of the ENO1p promoter. Again, CD36 produced a mixed colony morphology. Strain AV5 was the only wild-type strain capable of filamentous growth under these conditions (approximately 40% wrinkled), however this was greatly enhance following insertion of the ENO1p cassette (100% wrinkled). (TIF) [file pgen.1006373.s018.tif]

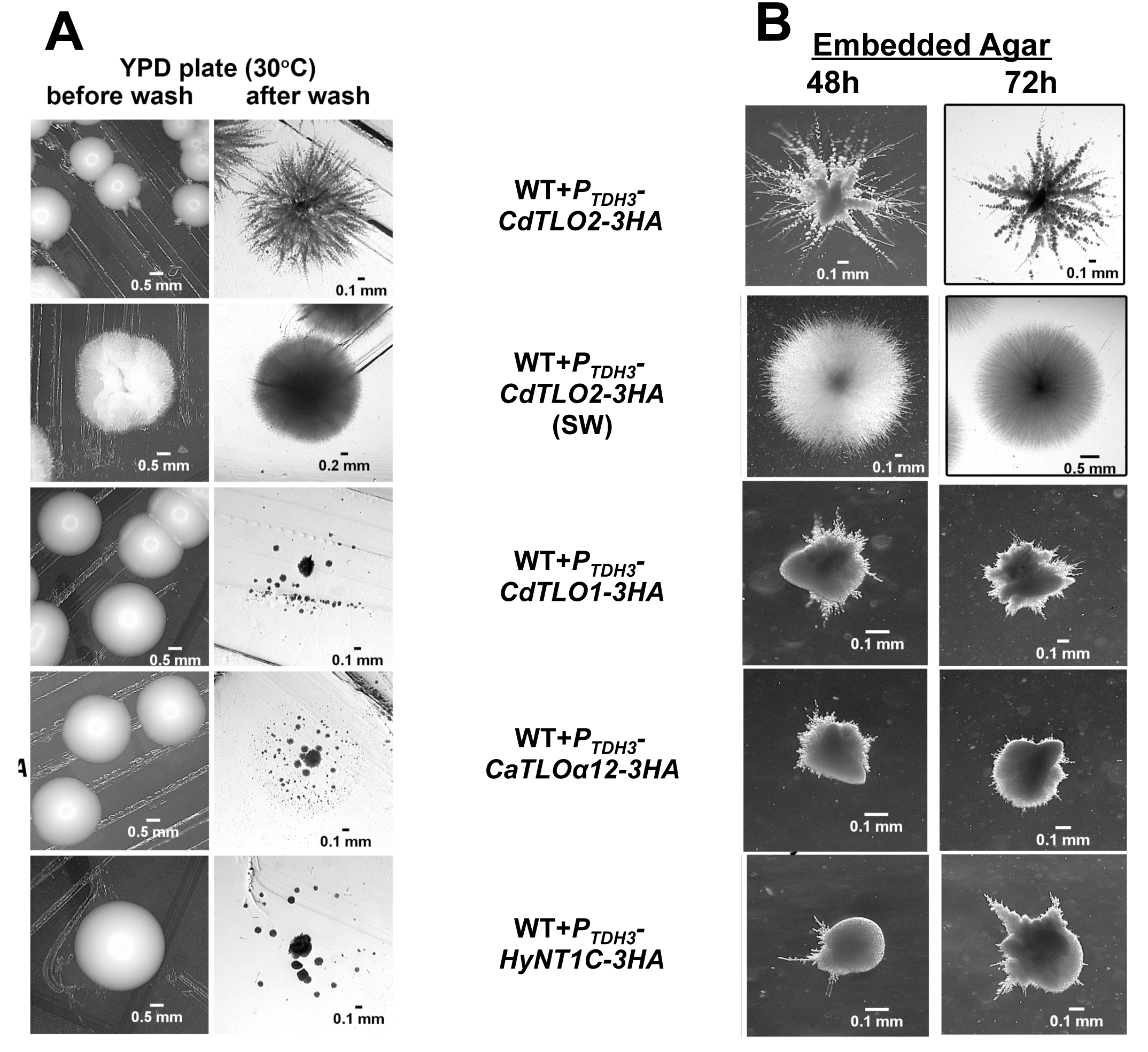

Supplement: S19 Fig — (A) Agar invasion phenotype in CdTLO2 (yLM339), CdTLO2 ‘SW” (yLM343) CdTLO1 (yLM337), CaTLOα12 (yLM335) and HyNT1C (yLM341) overexpression in a wild type (Wü284) C. dubliniensis strain monitored by resistance to washing. (B) Filamentation in embedded agar phenotype in CdTLO2 (yLM339), CdTLO2 ‘SW” (yLM343) CdTLO1 (yLM337), CaTLOα12 (yLM335) and HyNT1C (yLM341) overexpression in a wild type (Wü284) C. dubliniensis strain. (TIF) [file pgen.1006373.s019.tif]

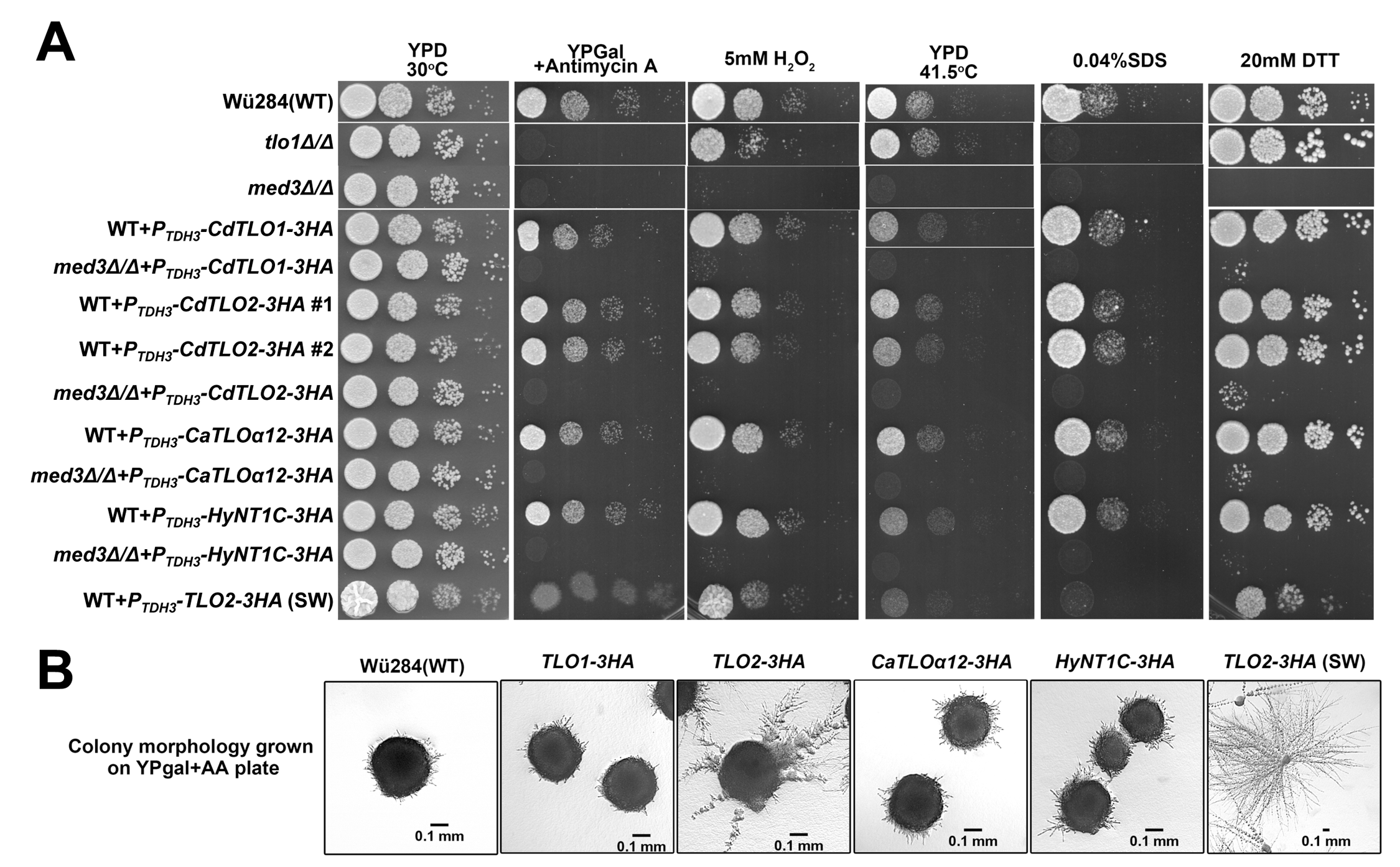

Supplement: S20 Fig — (A) Dilution series of strains over-expressing TLO genes in WT (Wü284 as the parental strain, yLM337 for CdTLO1 over-expression, yLM339 for CdTLO2, yLM335 for CaTLOα12 and yLM341 for HyNT1C) and med3Δ/Δ (yLM300 as the parental strain, yLM338 for overexpressing CdTLO1, yLM340 for CdTLO2, yLM336 for CaTLOα12 and yLM342 for HyNT1C) C. dubliniensis backgrounds. A tlo1Δ/Δ (yLM123) C. dubliniensis strain and the ‘SW’ transformant derived from CdTLO2-3HA1X transformation (yLM343) are also included in the comparison. Growth is tested on agar plates under the conditions listed above the each panel with the base conditions being YPD media at 30°C unless otherwise noted. CdTLO2 over-expression SW strains showed slow growth upon exposure to high temperatures (41.5°C) and SDS. (B) When utilizing galactose as carbon source, colonies from the CdTLO2 overexpression strains, particularly the ‘SW’ isolates, tend to undergo further enhanced filamentous growth. (TIF) [file pgen.1006373.s020.tif]

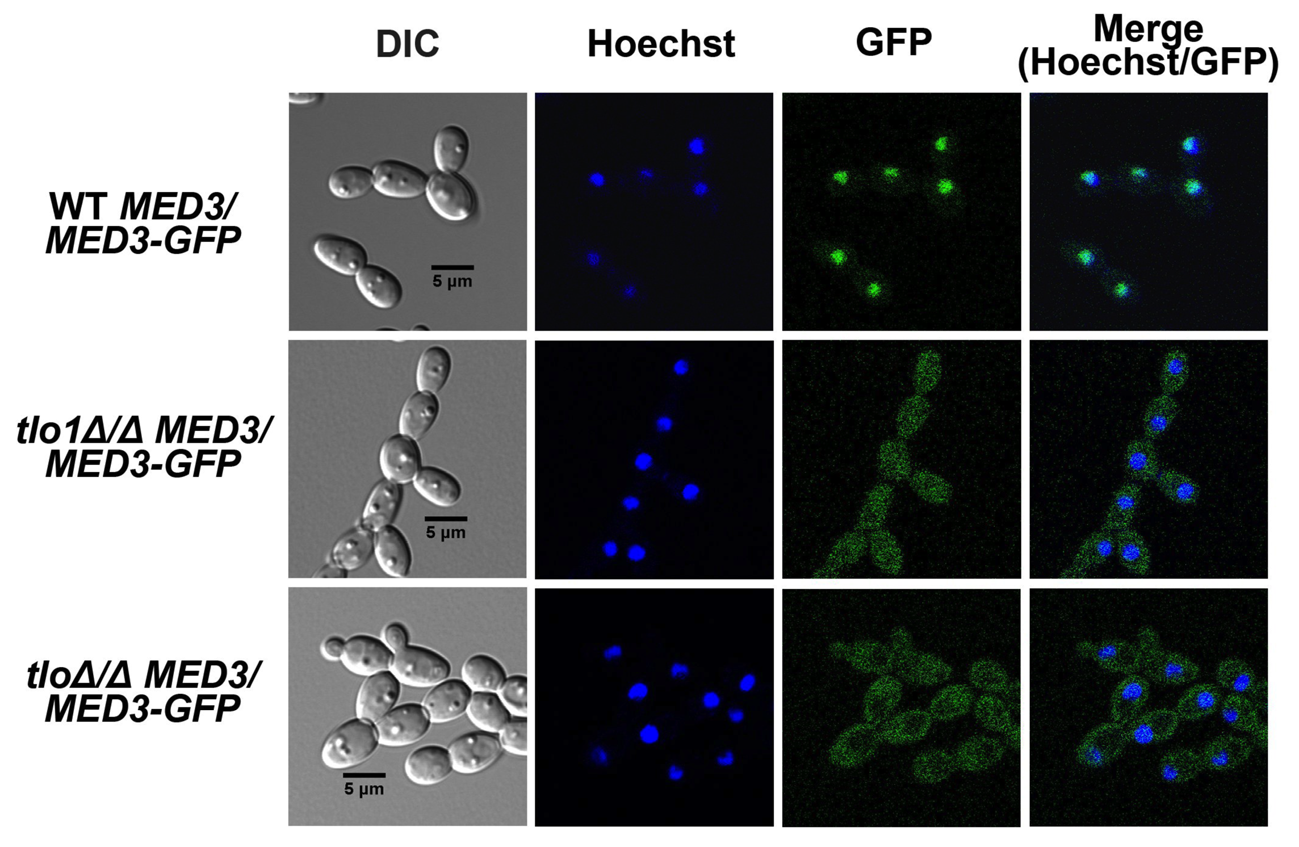

Supplement: S21 Fig — One endogenous copy of CdMed3 was GFP tagged at its C-terminus and its localization was observed in wild type (yLM359), tlo1Δ/Δ (yLM360) and tloΔ/Δ (yLM361) C. dubliniensis strains. Differential contrast (DIC) and fluorescence microscopy were used to visualize GFP localization, while Hoechst staining was used to stain the nuclei. All cells were grown in synthetic complete media overnight, diluted into the same media and grown for 5–6 hours before visualization. (TIF) [file pgen.1006373.s021.tif]

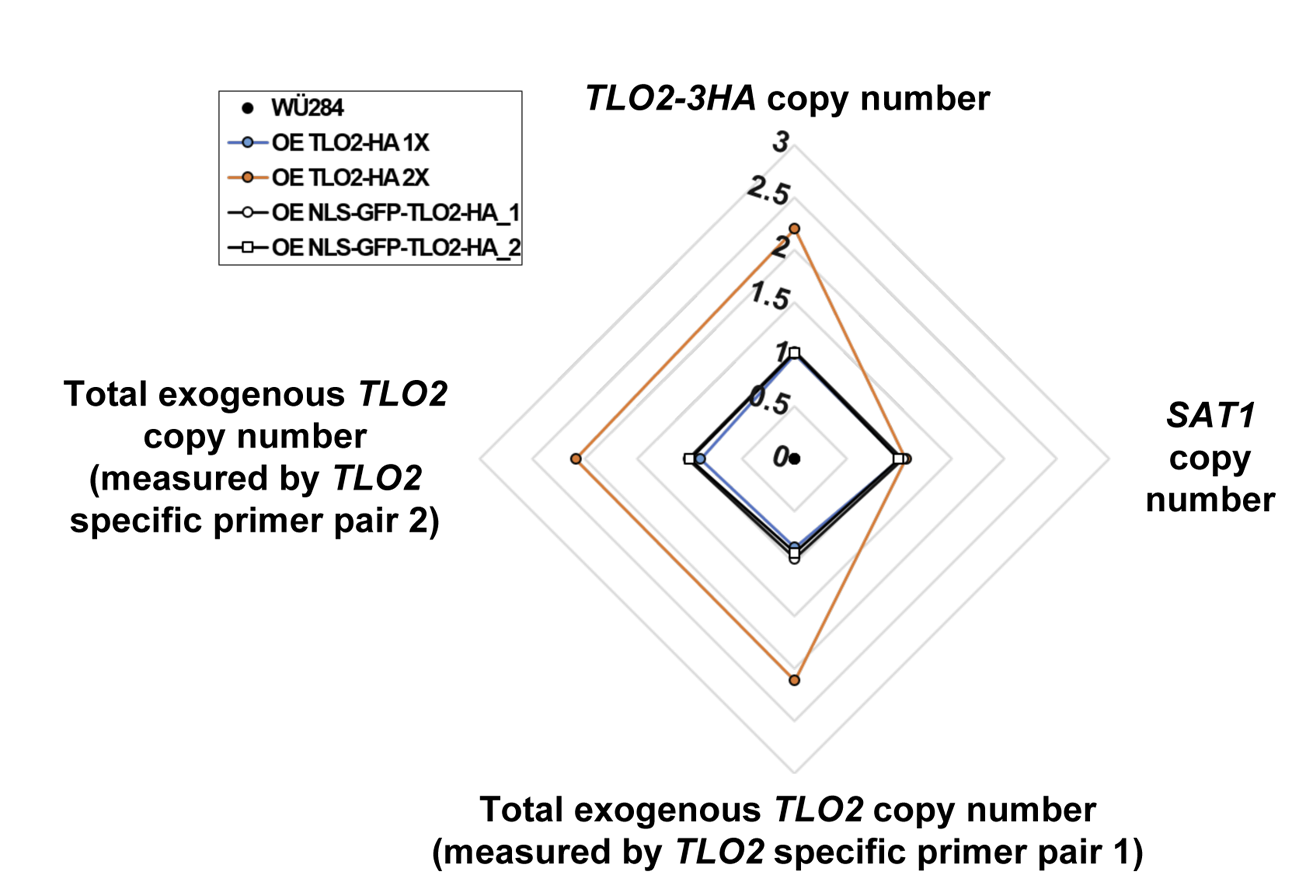

Supplement: S22 Fig — Radar plot of CdTLO2 copy number in strains containing CdTLO2-HA over-expression cassettes with and without a nuclear localization sequence. Genomic DNA of two independent transformants of a C. dubliniensis strain over-expressing TDH3 promoter-driven NLS-GFP-TLO2-HA (yLM367) was extracted from over-night YPD cultures and analyzed by qPCR to quantify the exogenous TLO2 ORF and SAT1 ORF copy number. The result is presented in comparison with the numbers obtained from C. dubliniensis strains over-expressing one copy (1X) and two copies (2X) of TDH3 promoter-driven TLO2-3HA (yLM339 and yLM344 respectively). (TIF) [file pgen.1006373.s022.tif]

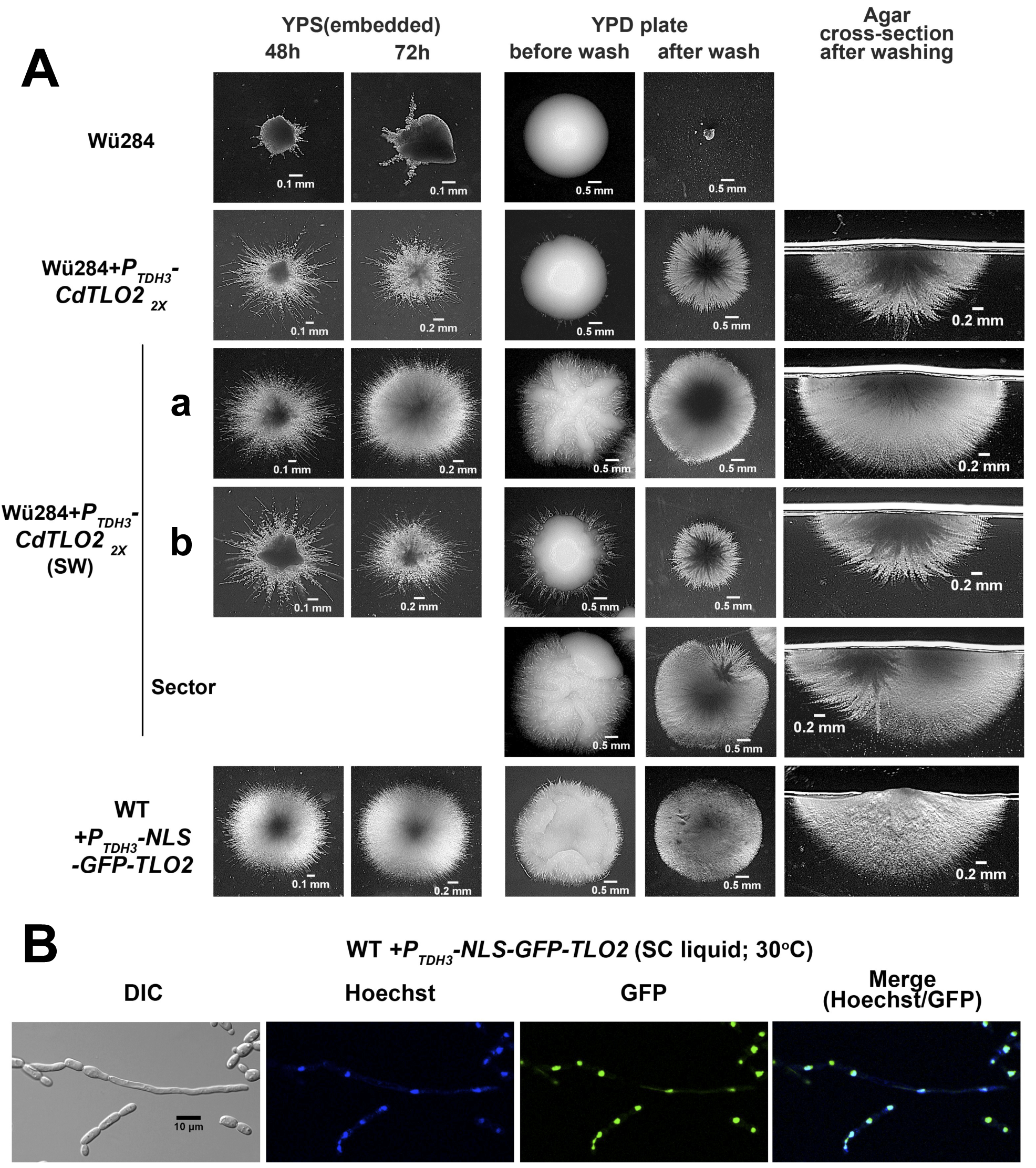

Supplement: S23 Fig — (A) Embedded agar filamentation (two left columns) and agar invasion (three right columns) phenotype analysis with two copies of CdTLO2 (yLM346), or one copy of NLS-GFP-TLO2 (yLM369) overexpressed from a TDH3 promoter in wild type (Wü284) C. dubliniensis strain. ‘SW’ colonies (yLM347), with multiple phenotypes (a & b), also occurred among the transformants with the CdTLO2 without the HA tag. (B) NLS-GFP-TLO2 overexpression strain (yLM369) grown in liquid media and visualized by differential contrast (DIC) and fluorescence microscopy were used to detect GFP localization, while Hoechst staining was used to stain the nuclei. All cells were grown in synthetic complete media overnight, diluted into the same media and grown for 5–6 hours before visualization. (TIF) [file pgen.1006373.s023.tif]

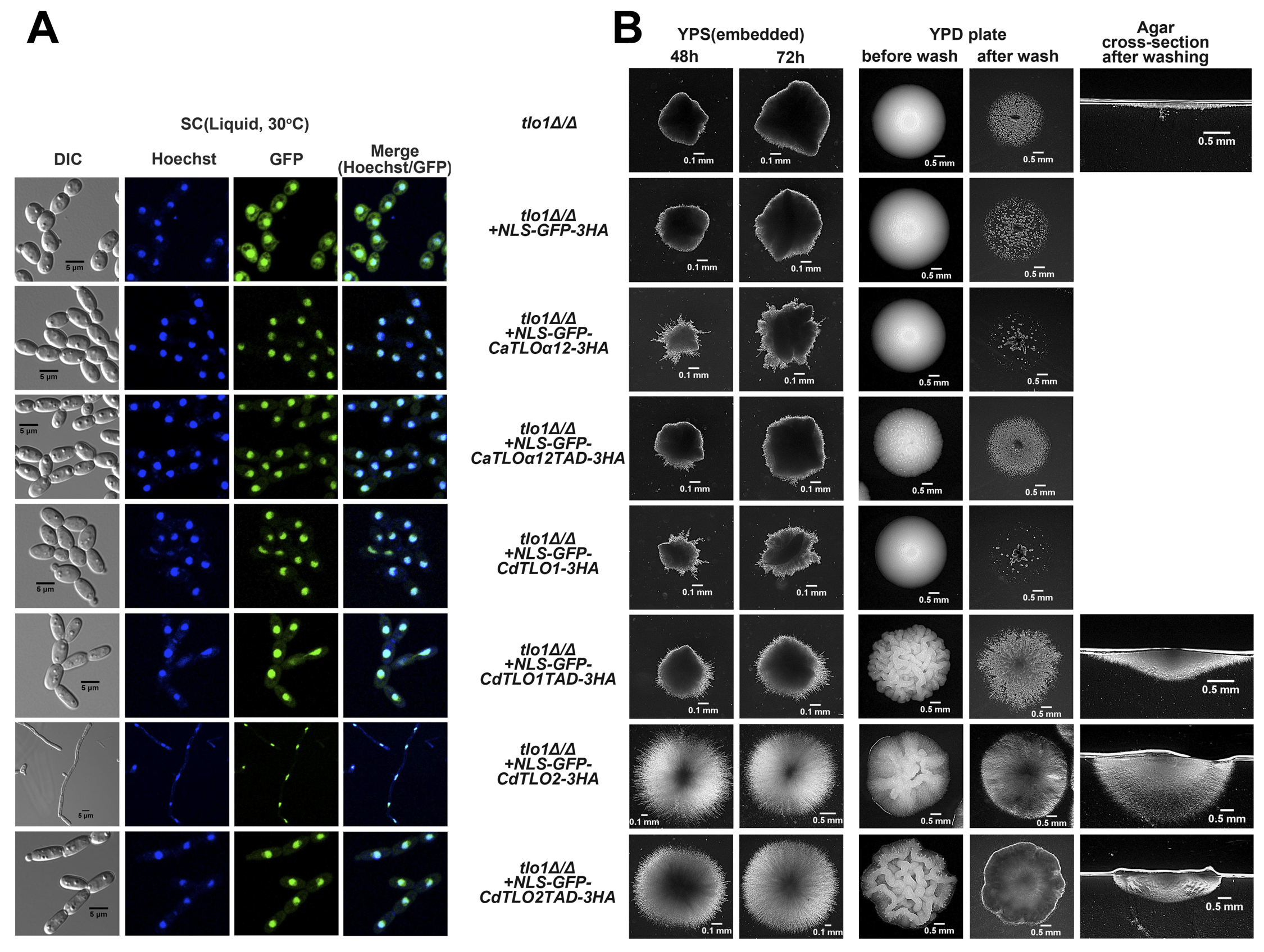

Supplement: S24 Fig — (A) TLO constructs, which were N-terminally fused with NLS-GFP sequence and C-terminally HA-tagged, were over-expressed from a TDH3 promoter in a tlo1Δ/Δ C. dubliniensis strain (yLM370 for over-expression of NLS-GFP-3HA, yLM371 for CaTLOα12, yLM373 for CdTLO1 and yLM375 for CdTLO2). The CaTLOα12TAD (yLM372) strain contained residues 164–252 of CaTLOα12. The CdTLO1TAD (yLM374) strain contained residues 199–320 of CdTLO1. The CdTLO2TAD strain (yLM376) contained residues 255–367 of CdTLO2. Differential contrast (DIC) and fluorescence microscopy were used to visualize GFP localization, while Hoechst staining was used to stain the nuclei. All cells were grown in synthetic complete media overnight, diluted into the same media and grown for 5–6 hours before visualization. (B) Embedded agar filamentation (two left columns) and agar invasion (three right columns) phenotype analysis with NLS-GFP C. dubliniensis strains described in A. (TIF) [file pgen.1006373.s024.tif]

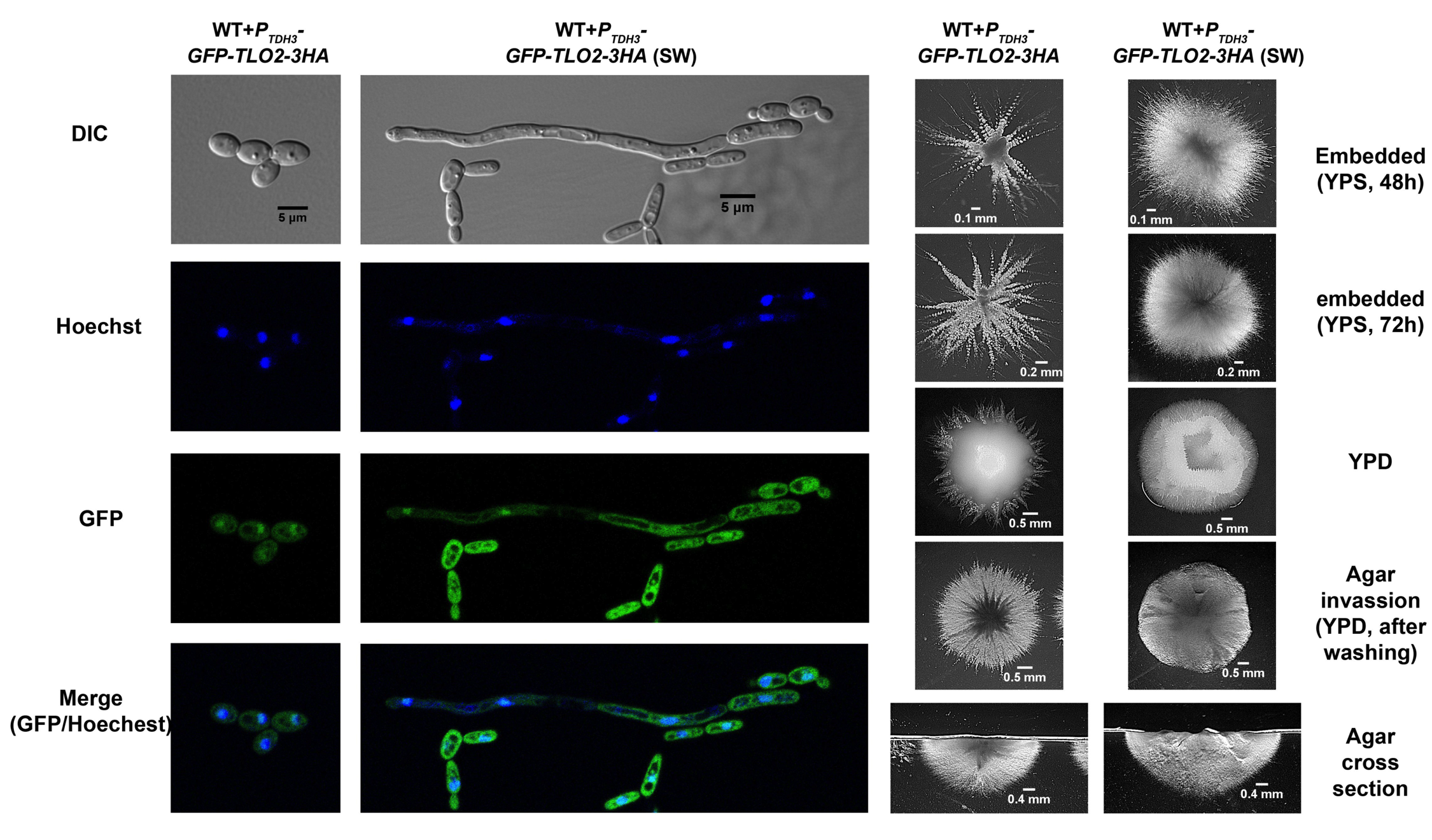

Supplement: S25 Fig — CdTLO2, which was N-terminally tagged with a GFP sequence and C-terminally HA-tagged, was overexpressed from a TDH3 promoter in a wild type (Wü284) C. dubliniensis strain, and produced transformants of both the ‘smooth’ (yLM384) and ‘SW’ (yLM385) colony phenotypes. (Left) Differential contrast (DIC) and fluorescence microscopy were used to visualize GFP localization, while Hoechst staining was used to stain the nuclei. All cells were grown in synthetic complete media overnight, diluted into the same media and grown for 5–6 hours before visualization. (Right) Embedded agar filamentation and agar invasion phenotype analysis with the GFP-CdTLO2 ‘smooth’ and ‘SW’ C. dubliniensis strains. (TIF) [file pgen.1006373.s025.tif]
