# Supplementary material for: Amplification of TLO Mediator Subunit Genes Facilitate Filamentous Growth in Candida Spp
Source: PLoS Genet. 2016 Oct 14;12(10):e1006373. doi: 10.1371/journal.pgen.1006373 (PMC5065183; doi:10.1371/journal.pgen.1006373)
Supplement: S1 Text — (PDF) [file pgen.1006373.s026.pdf]

S1 Text. Supplemental Material and Methods

Construction of integrating plasmids for *TLO1* and *TLOα12* overexpression in the *tloΔ/Δ C. dubliniensis* strain (yLM125)

Overexpression of native or C-terminal 3XHA tagged *CdTLO1* and *CaTLOα12* in *C. dubliniensis* was achieved by integrating each corresponding *CdTDH3* promoter (P_{TDH3}) driven construct in place of a single *TLO1* locus. Specifically, the *TLO1* ORF downstream region was amplified from *C. dubliniensis* genomic DNA by ZL286/ZL287 and inserted as a 3' homologous region (3'HR) into *pFA6a-3HA-SAT1* [1] between the PmeI/SacII sites to generate *pFA-3HA-SAT1-TLO1-3'*. The native promoter driven *TLO1* coding sequence, amplified by ZL282/ZL284, was inserted between the HindIII /BamHI sites, resulting in the *pFA-*P_{TLO1}*-TLO1-3HA* construct. To make the over-expression vector with one copy of P_{TDH3}-driven *TLO1-3HA*, the *TDH3* promoter sequence was amplified by ZL304/ZL305 from genomic DNA, fused to part of *TLO1* coding sequence (+1 to +63) through a second round of PCR by ZL304/ZL306, digested by NheI/StuI and inserted into *pFA-*P_{TLO1}*-TLO1-3HA* cut by the same enzymes. This intermediate construct was linearized by AscI for insertion of the MluI/AscI digested *CdACT1* terminator (T_{ACT1}) sequence, amplified by ZL302/ZL303 from genomic DNA. The plasmid, with the confirmed correct orientation of T_{ACT1}, was named as *pFA-*P_{TDH3}*-TLO1-3HA*$_{1X}$. To generate a single copy over-expression vector for *CaTLOα12-3HA*, P_{TDH3} sequence (amplified by ZL304/ZL307) and the *TLOα12* ORF (amplified by ZL309/ZL311) were sealed by fusion PCR using ZL304/ZL311. This DNA fragment was digested by NheI/BamHI and inserted into *pFA-*P_{TLO1}*-TLO1-3HA* backbone cut by the same enzymes and the resulting plasmid, after insertion of the T_{ACT1} sequence at the AscI site as described above, was named as *pFA-*P_{TDH3}*-TLOα12-3HA*$_{1X}$. *pFA-*P_{TDH3}*-TLO1-3HA*$_{2X}$, the vector containing two copies of P_{TDH3}-driven *TLO1-3HA,* was generated by ligating the P_{TDH3}*-TLO1-3HA-*T_{ACT1} cassette, which was digested from *pFA-*P_{TDH3}*-TLO1-3HA*$_{1X}$ by NheI/NaeI (referred to as 'NN'), to the backbone of the same plasmid digested by SpeI/PmeI (referred to as 'SP'). This 'doubling' strategy was also used to generate *pFA-*P_{TDH3}*-TLOα12-3HA*$_{2X}$. The 'NN' fragments from *pFA-*P_{TDH3}*-TLO1-3HA*$_{1X}$ and *pFA-*P_{TDH3}*-TLOα12-3HA*$_{1X}$ were swapped between constructs to generate *pFA-TLO1-3HA/TLOα12-3HA* and *pFA-TLOα12-3HA/TLO1-3HA* respectively. *pFA-*P_{TDH3}*-TLO1-3HA*$_{1X}$ and *pFA-*P_{TDH3}*-TLOα12-3HA*$_{1X}$ were also used as the templates to amplify P_{TDH3}-driven untagged *TLO1* and *TLOα12* by ZL304/ZL299 and ZL304/ZL320 respectively and individual fragment was digested by NheI/AscI and inserted into *pFA-*P_{TDH3}*-TLO1-3HA*$_{1X}$ backbone cut by the same enzymes. The resulting plasmids, after

we introduced T_{ACT1} at the AscI site, were used to generate *pFA-*P_{TDH3}*-TLO1*$_{2X}$ and *pFA-*P_{TDH3}*-TLOα12*$_{2X}$ following the 'doubling' strategy as described above.

Constructs for C-terminal 3XHA tagged *TLO1* and *TLOα12* overexpression in wild type *C. albicans* strains (SN152/BWP17)

Overexpression of C-terminal 3XHA tagged *TLO1* and *TLOα12* from *CaACT1* promoter (*pACT1*) in *C. albicans* was achieved by introduction of *pACT1-TLO1-3HA-SAT1* and *pACT1-TLOα12-3HA-SAT1* into the SN152 strain, or *pACT1-TLO1-3HA-URA3* and *pACT1-TLOα12-3HA-URA3* into BWP17 at the *RPS10* locus. To construct these plasmids, DNA cassettes containing *TLO1-3HA* and *TLOα12-3HA* were amplified from *pFA-*P_{TDH3}*-TLO1-3HA*$_{1X}$ and *pFA-*P_{TDH3}*-TLOα12-3HA*$_{1X}$, by ZL324/ZL325 and ZL187/ZL325 respectively, digested by HindIII/PstI and inserted to HindIII/PstI digested CIp-LexA backbone [2] to generate *pACT1-TLO1-3HA-URA3* and *pACT1-TLOα12-3HA-URA3*. The *URA3* selective maker, in the above constructs, was replaced by the *SAT1* marker for transformation of the *URA* prototrophic SN152 strain. For this purpose, *SAT1* marker was digested by PvuI/NcoI from *plac-LacZ-SAT1*, a vector derived from *plac-LacZ* [3]. The construction of *plac-LacZ-SAT1* involved multiple steps. The *plac-LacZ* was digested by NaeI/XbaI. One of the fragments, which contained the *LacZ* reporter and Amp^r marker, was circularized by ligating to the *SAT1*-containing fragment digested from *pFA-3HA-SAT1* by PvuII/XbaI. This construct was linearized by XbaI for the re-introduction of another fragment resulting from the previous NaeI/XbaI digestion of *plac-LacZ*, which contains *RPS10* sequence for targeting.

Constructs for overexpression of *CdTlo1/CaTloα12* chimeric proteins in the *tloΔ/Δ C. dubliniensis* strain (yLM125) and SN152 wild type *C. albicans* strain

Fusion PCR was used to make Series A and Series B chimeric genes **(S6 Table)**. For each hybrid gene, two fragments with overlapping sequence were respectively amplified by the indicated primer pair X and primer pair Y using either *pFA-*P_{TDH3}*-TLO1-3HA*$_{1X}$ or *pFA-*P_{TDH3}*-TLOα12-3HA*$_{1X}$ as the template **(S6 Table)**. The two DNA fragments were sealed and amplified by fusion PCR using ZL187/ZL325 (for Series A) or ZL304/ZL325 (for Series B) as primers. The resulting DNA cassette was digested by HindIII/PstI and inserted to *CIp-LexA* backbone cut by the same enzymes. Replacement of *URA3* marker with *SAT1* marker was performed as described above. Once cloned into the *pACT1*-driven *C. albicans* overexpression constructs

[listed in **S5 Table** as the *pACT1-(Hybrid Gene Name)-3HA-SAT1* plasmids], the hybrid genes were also sub-cloned into P_{TDH3}-driven *C. dubliniensis* overexpression system. For this purpose, each of the Series A hybrid genes were amplified from the corresponding *C. albicans* overexpression vector by ZL309/ZL325 and fused to P_{TDH3} sequence (amplified by ZL304/ZL307 as above) by fusion PCR using ZL304/ZL325. The resulting fragments were digested by NheI/AscI and individually ligated to *pFA-*P_{TDH3}*-TLOα12-3HA*$_{1X}$ backbone cut by the same enzymes, generating *pFA-*P_{TDH3}*-(12N-1)-3HA, pFA-*P_{TDH3}*-(12N-2)-3HA, pFA-*P_{TDH3}*-(12N-3)-3HA and pFA-*P_{TDH3}*-(12N-4)-3HA* respectively. To sub-clone the Series B chimeric genes into the *pFA-*P_{TDH3} *C. dubliniensis* overexpression constructs, each ORF was digested from the corresponding *pACT1*-driven *C. albicans* overexpression construct by StuI/AscI and inserted into *pFA-*P_{TDH3}*-TLO1-3HA*$_{1X}$ backbone cut by the same enzymes, resulting in the following constructs: *pFA-*P_{TDH3}*-(TN-1)-3HA, pFA-*P_{TDH3}*-(TN-2)-3HA, pFA-*P_{TDH3}*-(TN-3)-3HA*, *pFA-*P_{TDH3}*-(TN-4)-3HA* and *pFA-*P_{TDH3}*-(TN-5)-3HA.*

Other chimeric genes in Series A and Series B, not included in **S6 Table**, were generated using different strategies. The PCR product amplified from *pFA-*P_{TDH3}*-TLOα12-3HA*$_{1X}$ by ZL350/ZL325 was digested by StuI/AscI and inserted into *pFA-*P_{TDH3}*-TLO1-3HA*$_{1X}$ backbone cut by the same enzymes to generate *pFA-*P_{TDH3}*-(TN-6)-3HA.* The P_{TDH3}*-(TN-7)-3HA-*T_{ACT1} cassette was the fusion PCR product created by P_{TDH3} sequence amplified by ZL304/ZL305 and the DNA fragment amplified from *pFA-*P_{TDH3}*-TLOα12-3HA*$_{1X}$ by ZL351/ZL325; the P_{TDH3}*-T1N12C-3HA-*T_{ACT1} cassette was the fusion PCR product created by the fragment amplified from *pFA-*P_{TDH3}*-TLO1-3HA*$_{1X}$ by ZL304/ZL322 and the fragment amplified from *pFA-*P_{TDH3}*-TLOα12-3HA*$_{1X}$ by ZL323/ZL325. These two DNA cassettes were introduced into a *C. dubliniensis* one copy overexpression construct to generate *pFa-*P_{TDH3}*-(TN-7)-3HA* and *pFA-*P_{TDH3}*-T1N12C-3HA* respectively through NheI/AscI digestion and ligation as described above. *12NT1C-3HA* was first generated by a three parts ligation among the HindIII/EcoRI digested PCR fragment amplified from *pFA-*P_{TDH3}*-TLOα12-3HA*$_{1X}$ by ZL187/ ZL321, the EcoRI/AscI digested fragment from *pFA-*P_{TDH3}*-TLO1-3HA*$_{1X}$, which contained the coding sequence for Tlo1-3HA C' terminal part, and HindIII/AscI digested *pFA-3HA-SAT1-TLO1-3'. 12NT1C-3HA* then was amplified by ZL309/ZL325, fused to *TDH3* promoter, digested by NheI/AscI and inserted into *pFA-*P_{TDH3}*-TLO1-3HA*$_{1X}$ backbone cut by the same enzymes, resulting in *pFA-*P_{TDH3}*-12NT1C-3HA*. The above four chimeric genes were also amplified by ZL187/ZL325 (for *12NT1C*) or by ZL324/ZL325 (for *TN-6*, *TN-7*, and *T1N12C*) and sub-cloned into HindIII/PstI digested *CIp-LexA*.

Replacement of *URA3* marker with *SAT1* marker was performed as described above, resulting in the corresponding *pACT1*-driven *C. albicans* overexpression constructs.

The remaining chimeric genes used in this study were constructed as described below and in **S7 Table**: for each hybrid gene, two fragments with overlapping sequence were individually amplified by the indicated primer pair X and primer pair Y from the listed vectors. The two DNA fragments were sealed and amplified by fusion-PCR using ZL282/ZL325. Each of the resulting fragments, which contained (from 5' to 3') 5' HR for *CdTLO1* locus targeting, *TDH3* promoter sequence, and the *3XHA* tagged chimeric gene, were digested by HindIII/BamHI and cloned into *pFA-*P_{TDH3}*-TLO1-3HA*$_{1X}$ backbone digested by the same enzymes to generate each corresponding plasmid listed in **S7 Table**. A construct over-expressing untagged *HyNT1C* was made by insertion of the HindIII/AscI digested PCR product, which was amplified from *pFA-*P_{TDH3}*-HyNT1C-3HA*$_{1X}$ by ZL282/ZL299, into *pFA-*P_{TDH3}*-TLO1-3HA*$_{1X}$ backbone cut by the same enzymes. The resulting plasmid, after insertion of the T_{ACT1} sequence at its AscI site, was named as *pFA-*P_{TDH3}*-HyNT1C*$_{1X}$. *pFA-*P_{TDH3}*-HyNT1C-3HA*$_{1X}$ and *pFA-*P_{TDH3}*-HyNT1C*$_{1X}$ were used to construct *pFA-*P_{TDH3}*-HyNT1C-3HA*$_{2X}$ and *pFA-*P_{TDH3}*-HyNT1C*$_{2X}$ respectively, which contain either two copies of P_{TDH3}-driven *HyNT1C-3HA* or P_{TDH3}-driven untagged *HyNT1C*, following the 'doubling' strategy described above. One intermediate DNA product of the 'doubling' process, the 'NN' fragment (above) of *pFA-*P_{TDH3}*-HyNT1C-3HA*$_{1X}$, was also ligated to the 'SP' fragment (above) of *pFA-*P_{TDH3}*-TLO1-3HA*$_{1X}$ to generate *pFA-TLO1-3HA/HyNT1C-3HA*. *HyNT1C-3HA* was also cloned into *pACT1*-driven *C. albicans* over-expression vector following the strategy as described above. Additionally, DNA sequence encoding the C-terminal *3XHA* tag was removed from *pFA-*P_{TDH3}*-HyNΔC-3HA*$_{1X}$ by digesting the plasmid with BamHI and circularizing the digested backbone by self-ligation to generate *pFA-*P_{TDH3}*-HyNΔC*$_{1X}$, which after 'doubling' the over expression cassettes, became *pFA-*P_{TDH3}*-HyNΔC*$_{2X}$.

Constructs for expression of C-terminal *6His3FLAG* tagged *TLO* variants

These constructs were used for expression of C-terminal *6His3FLAG* tagged *TLO* variants from *TDH3* promoter at *TLO1* locus. Specifically, a 3' HR amplified by ZL286/ZL287 for *TLO1* locus targeting was inserted into *pFA-6His3FLAG-SAT1* [1] between the PmeI/SacII sites to generate *pFA-6His3FLAG-SAT1-TLO1-3'*. In order to create *pFA-*P_{TDH3}*-TLOα12-6His3FLAG*$_{2X}$, the [P_{TDH3}-*TLOα12*] DNA cassette cut from *pFA-*P_{TDH3}*-TLOα12-3HA*$_{1X}$ by HindIII/BamHI was placed into *pFA-6His3FLAG-SAT1-TLO1-3'*. Introducing the T_{ACT1} sequence at the AscI site and 'doubling'

the [P_{TDH3}-*TLOα12-6His3FLAG*] cassette were performed as described above. To generate the constructs for expression of the C-terminal 6His3FLAG tagged chimeric protein in **Fig 3 and S14 Fig**., each indicated coding sequence, together with *TDH3* promoter and the 5' HR for targeting the *CdTLO1* locus, was removed from the corresponding *pFA-*P_{TDH3}-3HA plasmid by HindIII/BamHI digestion and sub-cloned into the *pFA-6His3FLAG-SAT1-TLO1-3'* backbone cut by the same enzymes. Following the same strategy, *pFA-TLO2-6His3FLAG*$_{1X}$*-SAT1* was generated from *pFA-TLO2-3HA-SAT1* (See below for construction details).

pFA-P_{TDH3}- *TLO2-6His3FLAG*$_{2X}$ was derived from *pFA-*P_{TDH3}- *TLO2-6His3FLAG*$_{1X}$ through the 'doubling' strategy as described above and additional *TLO1* upstream sequence was inserted into this construct as described below to generate *EXpFA-*P_{TDH3}*-TLO2-6His3FLAG*$_{2X}$ for over-expressing and purifying Tlo2 in the Wü284 wild type *C. dubliniensis* strain.

Construction of additional *pFA-*P_{TDH3} plasmids used in this study

To generate *pFA-*P_{TDH3}*-TLO2-3HA*$_{1X}$, the parental construct for other *CdTLO2* overexpression vectors, the *CdTLO2* ORF was amplified from *C. dubliniensis* genomic DNA by ZL362/ZL361, fused to *TDH3* promoter sequence amplified by ZL304/ZL307, digested by NheI/BamHI and inserted into *pFA-*P_{TDH3}*-TLO1-3HA*$_{1X}$ backbone cut by the same enzymes. Notably, a G->A (+1037) silent mutation was intentionally introduced to the *CdTLO2* coding sequence by the ZL361 primer to allow for the use of ZL313/ZL201 as a versatile qPCR primer pair to directly compare mRNA levels of C-terminal 3XHA tagged *CdTLO1*, *CdTLO2*, *CaTLOα12* and their derivatives between strains.

The constructs (*pFA-*P_{TDH3}*-TLO1-GFP* and *pFA-*P_{TDH3}*-TLOα12-GFP*) for overexpression of C-terminal *GFP* tagged *CdTLO1* and *CaTLOα12*, were made by amplifying the GFP coding sequence from pMB126 [4] with ZL401/402, digesting the fragment by BamHI/AscI, and insertion of the fragment into *pFA-*P_{TDH3}*-TLO1-3HA*$_{1X}$ and *pFA-*P_{TDH3}*-TLOα12-3HA*$_{1X}$ backbone cleaved by the same enzymes.

Constructs for overexpression of native or C-terminal 3XHA tagged *TLO* variants in wild type (Wü284) and *med3Δ/Δ* (yLM300) *C. dubliniensis* strains

The short 5'HR for *TLO1* locus targeting (~200bp DNA sequence between the HindIII/NheI sites) makes the *pFA-P*$_{TDH3}$ constructs unable to efficiently target the intact *CdTLO1* loci within WT and *med3Δ/Δ C. dubliniensis* strains, since the recombination event could happen between the *TLO1* ORF on the genome and the *TLO* variant ORF carried by the overexpression construct and result in the loss of *P*$_{TDH3}$ sequence after integration. To overcome this deficiency, additional *TLO1* gene up-stream sequence was amplified by ZL458/ZL459, digested by HindIII and inserted into HindIII linearized *pFA-P*$_{TDH3}$ vectors including *pFA-P*$_{TDH3}$*-TLO1-3HA*$_{1X}$, *pFA-P*$_{TDH3}$*-TLOα12-3HA*$_{1X}$, *pFA-P*$_{TDH3}$*-TLO2-3HA*$_{1X}$, *pFA-P*$_{TDH3}$*-HyNT1C-3HA*$_{1X}$, *pFA-P*$_{TDH3}$*-TLO1-GFP, pFA-P*$_{TDH3}$*-TLOα12-GFP, pFA-P*$_{TDH3}$*-T1NT2C-3HA, pFA-P*$_{TDH3}$*- T2NT1C-3HA and pFA-P*$_{TDH3}$*-HyNT2C-3HA* to in the appropriate orientation to generate the corresponding '*ExpFA-P*$_{TDH3}$' version.

To introduce a second copy of the *CdTLO2-3HA* overexpression cassette into *ExpFA-P*$_{TDH3}$*-TLO2-3HA*$_{1X}$*, pFA-P*$_{TDH3}$*-TLO2-3HA*$_{1X}$ was digested by AscI, blunted by Klenow fragment and then cut by NheI. The resulting fragment, which contains the *P*$_{TDH3}$*-TLO2-3HA* cassette, was inserted into *ExpFA-P*$_{TDH3}$*-TLO2-3HA*$_{1X}$, digested by Spe1/Pme1, to generate Ex*pFA-P*$_{TDH3}$*-TLO2-3HA*$_{2X}$.

To generate *ExpFA-P*$_{TDH3}$*-TLO2-GFP*, the 3XHA tag coding sequence between the BamHI/AscI sites of *ExpFA-P*$_{TDH3}$*-TLO2-3HA* was replaced by GFP coding sequence cut from *pFA-P*$_{TDH3}$*-TLO1-GFP* by the two enzymes.

To generate an overexpression construct with two copies of untagged *CdTLO2* driven by the *TDH3* promoter, *ExpFA-P*$_{TDH3}$*-TLO2-3HA*$_{1X}$ was digested by NaeI/AscI and filled in with native *CdTLO2* sequence amplified from genomic DNA by ZL068/ZL497 to generate *ExpFA-P*$_{TDH3}$*-TLO2*$_{1X}$. Following the same strategy that we used to generate Ex*pFA-P*$_{TDH3}$*-TLO2-3HA*$_{2X}$ from Ex*pFA-P*$_{TDH3}$*-TLO2-3HA*$_{1X}$, a second copy of the *P*$_{TDH3}$*-TLO2* cassette was cloned into *ExpFA-P*$_{TDH3}$*-TLO2*$_{1X}$ to generate *ExpFA-P*$_{TDH3}$*-TLO2*$_{2X}$. *CdTLO2* coding sequence in *ExpFA-P*$_{TDH3}$*-TLO2*$_{2X}$ and its parental plasmid *ExpFA-P*$_{TDH3}$*-TLO2*$_{1X}$ does not contain the G->A mutation mentioned above, since it has been corrected by introducing the *TLO2* native genomic sequence amplified by ZL068/ZL497 in the first step.

For the *TLO2-3HA* overexpression construct *EXpFA-P*$_{ACT1}$*-TLO2-3HA*, *CaACT1* promoter was utilized in place of the *CdTDH3* promoter. Specifically, the *pACT1-TLOα12-GFP-SAT1* plasmid

(see below) was digested by HindIII, blunted by Klenow, and further digested by AscI to remove the fragment that contains *TLOα12-GFP* sequence. The resulting cut vector was ligated to a *TLO2-3HA* cassette, which was released from *pFA-P$_{TDH3}$-TLO2-3HA*$_{1X}$ by sequentially treating the plasmid with PacI restriction enzyme, Klenow and AscI. This intermediate construct, *pACT1-TLO2-3HA-SAT1*, was digested by ApaI, blunted by Klenow fragment and further digested by AscI to release a *pACT1-TLO2-3HA* cassette, which was then inserted into the plasmid backbone generated by sequentially treating *EXpFA-P$_{TDH3}$-TLOα12-3HA*$_{1X}$ with KpnI, Klenow fragment and AscI. The resulting plasmid is the final form of *EXpFA-P$_{ACT1}$-TLO2-3HA*.

Constructs for overexpression of *NLS-GFP-TLO* variants in *C. dubliniensis*

To generate the parental vector, *EXpFA-NLS-GFP-3HA*, we fused SV40 nuclear localization signal (NLS) coding sequence to the 5' end of the GFP gene by using ZL505/ZL506 to amplify GFP coding sequence from *pFA-P$_{TDH3}$-TLO1-GFP.* The resulting DNA fragment was fused to ZL304/ZL307 amplified *TDH3* promoter sequence, digested by AgeI/BamHI and ligated into the *ExpFA-P$_{TDH3}$-TLO2-3HA*$_{1X}$ backbone cut by the same enzymes. *CdTLO1*, *CdTLO1TAD*, *CdTLO2*, *CdTLO2TAD*, *CaTLOα12* and *CaTLOα12TAD* were amplified by using ZL511, ZL227, ZL503, ZL504, ZL168 and ZL169 respectively as the forward primer and ZL325 as the common reverse primer from the corresponding *pFA-P$_{TDH3}$-3HA* plasmids. Each fragment was digested by BamHI/Mlu1 and inserted into the *EXpFA-NLS-GFP-3HA* backbone digested by the same enzymes. *EXpFA-NLS-GFP-TLO2,* which was used to overexpress the C-terminal untagged form of NLS-GFP-TLO2, was generated by replacing the DNA sequence between the XhoI/AscI sites of *EXpFA-NLS-GFP-TLO2-3HA* with the counterpart from *ExpFA-P$_{TDH3}$-TLO2*$_{1X}$.

Constructs used for Tlo and Med3 protein localization studies

The construction of *EXpFA-P$_{TDH3}$-TLO1-GFP, EXpFA-P$_{TDH3}$-TLOα12-GFP and EXpFA-P$_{TDH3}$-TLO2-GFP*, which were used to express each C-terminal GFP tagged Tlo protein in *C. dubliniensis* WT and *med3Δ/Δ* strains, has been described above.

To express *TLOα12-GFP* driven by *ACT1* promoter in wild type *C. albicans* (SN152), *med3Δ/Δ* (yLM119)*, med15Δ/Δ* (cTTR01) *and med16Δ/Δ* (AZC34) strains, we constructed *pACT1-TLOα12-GFP-SAT1* by the following strategy: amplifying a DNA fragment that contains the coding sequence of C-terminally GFP tagged Tloα12 and *SAT1* selective marker from *pFA-*

P_{TDH3}-*TLOα12-GFP* by ZL187/ZL287, digesting it with HindIII/PstI and inserting the digested fragment into the CIp-LexA backbone cut by the same enzymes.

To C-terminal-GFP tag *CdMED3* at its native locus in wild type (Wü284), *tlo1Δ/Δ* (yLM123) and *tloΔΔ* (yLM125) *C. dubliniensis* strains, first, an intermediate vector, *pFA-P_{TDH3}-MED3-3HA* was generated by fusing *TDH3* promoter sequence to the *MED3* ORF amplified by ZL308/ZL310 and using the resulting fragment to replace the P_{TDH3}-*TLO1* cassette between the NheI/BamHI sites of *pFA-P_{TDH3}-TLO1-3HA$_{1X}$*. The GFP coding sequence was cut from *pFA-P_{TDH3}-TLO1-GFP* by BamHI/AscI and inserted into the *pFA-P_{TDH3}-MED3-3HA* backbone digested by the same enzymes to generate *pFA-P_{TDH3}-MED3-GFP.* In the last step, the 3'HR for *TLO1* locus targeting between the PmeI/SacII sites was removed from *pFA-P_{TDH3}-MED3-GFP*, allowing the insertion of the 3'HR for *CdMED3* locus targeting, which was amplified from genomic DNA by ZL396/ZL397 and digested by SnaBI/SacII. The resulting plasmid was named as *pFA-gCdMED3-GFP*. Digestion of this plasmid through a HindIII site within the *MED3* coding sequence and the SacII site downstream of the 3'HR released the DNA fragment for C-terminal GFP tagging endogenous *CdMED3* with a *SAT1* selective marker.

Introducing *TLO2* over-expression cassettes into *C. dubliniensis* resulted in transformants with two different colony morphologies, smooth or super-wrinkled, as described in the main text. *EXpFA-P_{TDH3}-GFP-TLO2-3HA* was constructed for comparing CdTlo2p cellular localization between the two types of *C. dubliniensis* colonies. Specifically, PCR product amplified by ZL510/ZL427 from *EXpFA-NLS-GFP-TLO2-3HA* was fused to *TDH3* promoter sequence (amplified by ZL304/ZL307), digested by AgeI/XhoI and inserted into EXpF*A-P_{TDH3}-TLO2-3HA*$_{1X}$ backbone cut by the same enzymes.

Ploidy analysis in *C. dubliniensis*

The nine genomic loci used for ploidy analysis represent the left arms of all *C. dubliniensis* chromosomes as assembled in the current GCD database except Chromosome 6 and the right arms of Chromosome 7 and Chromosome X. Each locus was annotated as Chr(Chromosome)_'# (chromosome in the assembled *C. dubliniensis* genome contains the given locus)_L/R ('L' and 'R' stand for 'Left' and 'Right' respectively indicating the locus is on which arm of the chromosome)_K(chromosome(s) in *C. dubliniensis* karyotype that contain the locus).

The primers used to detect each locus are: ZL724/ZL725 for Chr1_L(XIII), ZL386/ZL387 for Chr2_L(X/IX), ZL728/ZL729 for Chr3_L(VII), ZL730/ZL331 for Chr4_L(V/VI), ZL809/ZL810 for Chr5_L(I/VIII), ZL813/ZL814 for Chr7_L(II/V), ZL815/ZL816 for Chr7_R(II/X), ZL817/ZL818 for ChrR_L(XI/XII) and ZL456/ZL457 for ChrR_R(VIII/XII). To perform the analysis, genomic DNA was extracted from 5 mL over-night YPD culture of the testing strains and the reference strain Wü284 following the protocol described in [5], but instead of TE buffer the final product was re-suspended in 400 µL Qiagen EB buffer. After concentration measurement, DNA samples were diluted to 10 ng/µL in EB and 2.5 µL (25 ng genomic DNA per testing reaction) of the diluted samples were mixed into 20 µL SYBR-Green real-time PCR reactions for quantification. The real-time PCR program was set and analyzed using the 'relative standard curve' method. For testing a given locus, first, a linear standard curve was set by using 6.25 ng, 12.5 ng, 25 ng and 50 ng of Wü284 genomic DNA with the reading for 25 ng reaction set to '1' (accordingly 6.25 ng reaction to '0.25', 12.5 ng reaction to '0.5', and 50 ng reaction to '2') and then the relative concentration of DNA molecule containing that locus in a testing sample could be calculated by matching its Ct value to the linear standard curve. Strains maintaining the same ploidy as Wü284 are expected to have value around '1' for all the nine loci included in the test. Also, for strains maintaining their parental ploidy, inaccuracy during DNA concentration measurement or dilution will lead to an overall increase or decrease value at all the testing loci, which was eliminated through normalization by setting the value from any locus to '1' (in this study, Chr1_L(XIII) was chosen for this purpose). A value greater than 1.5 which was not able to be eliminated by normalization indicates one or more extra copies of the corresponding locus (and likely its representing chromosome) are acquired by a strain during genetic modification, while a value of 0.5 which is not able to be eliminated by normalization suggested missing a copy of the corresponding locus (and likely its representing chromosome). Polyploidy is not able be revealed by the above analysis.

Quantification of exogenous *TLO2* copy number in *C. dubliniensis TLO2* over-expression strains

Exogenous *TLO2* copy number in an over-expression strain was quantified by a qPCR based analysis on the genomic DNA sample prepared from over-night YPD culture of the strain, similar to the method used for ploidy analysis.

TLO2 copy number in a given strain was determined by qPCR using yLM339, yLM344 and Wü284 as reference strains. As described in detail below, yLM339 was confirmed to have a single insertion of *TLO2-HA$_{1X}$* cassette (one copy of *TLO2-HA* driven by a *TDH3* promoter followed by one *SAT1* marker) and yLM344 was confirmed to have a single insertion of *TLO2-HA$_{2X}$* cassette (two copies of *TLO2-HA* driven each by a *TDH3* promoter spaced by one *SAT1* marker). Based on this information, exogenous *TLO2* copy number in other HA-tagged *TLO2* over-expression strains (yLM343, yLM345 and yLM367) was quantified when their genomic DNA was compared with the genomic DNA of yLM339 and yLM344 by qPCR using the primer pair ZL313/ZL201. Two other *TLO2* specific primer pairs, '*TLO2_1*'(ZL426/ZL427) and '*TLO2_2*'(ZL424/425), which respectively anneal in the middle and close to the 5' end of the*TLO2* ORF, were also used to evaluate exogenous *TLO2* copy number as described below. In addition, *SAT1* copy number was tested by the primer pair of ZL819/ZL820 to reveal the integrity of over-expression cassettes during integration.

We first examined yLM339 (one copy *TLO2-HA* driven by a *TDH3* promoter; smooth) and yLM344 (two copies *TLO2-HA* each driven by a *TDH3* promoter; smooth) with the parental wild type *C. dubliniensis* strain (Wü284) as the reference. No aneuploidy was detected in yLM339 and yLM344. Identical amounts (25 ng) of genomic DNA from each strain was amplified by ZL426/ZL427 (primers pair '*TLO2_1*', specifically annealing in the middle of the *TLO2* coding region) and ZL724/ZL725 in qPCR reaction to compare their relative *TLO2* ORF and Chr1_L(XIII) locus contents. After normalized to the Chr1_L(XIII) locus, *TLO2* ORF contents of Wü284, yLM339 and yLM344 showed a 2: 2.92: 4.06 ratio. This result is consistent with the theoretical ratio, 2:3:4, assuming Wü284 has two copies of endogenous *TLO2* gene (one allele might be partially truncated close to the 5' end due to a chromosome events) and yLM399 has three copies in total (two endogenous copies and one exogenous copy introduced by a single insertion of the *TLO2-HA$_{1X}$* cassette) and yLM344 has four copies in total (two endogenous copies and two exogenous copies introduced by a single insertion of the *TLO2-HA$_{2X}$* cassette). A similar result (Wü284:yLM339:yLM344=2:1.84:4.20) was observed by using ZL424/ZL425 (primers pair '*TLO2_2*'), which anneal close to the 5' of the *TLO2 ORF*. With the above analysis, exogenous *TLO2* copy number in other *C. dubliniensis* strains over-expressing HA-tagged *TLO2* (yLM343, yLM345 and yLM367) was quantified when their genomic DNA was compared with the genomic DNA of yLM339 (one copy reference) and yLM344 (two copies reference) by qPCR using the primer pair ZL313/ZL201.

Exogenous *TLO2* copy number in the non-tagged *TLO2* over-expressing strain (yLM347) was measured by primer pair '*TLO2_1*' and '*TLO2_2*' using Wü284 as the reference strain. Similar to the method described above, relative *TLO2* ORF contents on genomic DNA was measured in yLM347 and Wü284 after normalization to Chr1_L(XIII) locus contents. The absolute exogenous *TLO2* ORF copy number in yLM347 was calculated by {[yLM347 contents]/[Wü284 contents]-1}*2. This method was also applied to HA-tagged *TLO2* over-expression strains to confirm results obtained from primer pair ZL313/ZL201.

As a part of the *TLO2* over-expression cassettes, *SAT1* ORF copy number was also evaluated in strains to determine whether the any extra copies of the *TLO2* ORF also carried the *SAT1* cassette with them. *SAT1* copy number was quantified by primer pair ZL819/ZL820 through the same method used for *TLO2-HA* contents measurement (ZL313/ZL201).

References

1. Zhang A, Petrov KO, Hyun ER, Liu Z, Gerber SA, et al. The Tlo proteins are stoichiometric components of Candida albicans mediator anchored via the Med3 subunit. Eukaryot Cell 2012;11: 874-884.
2. Russell CL, Brown AJ . Expression of one-hybrid fusions with Staphylococcus aureus lexA in Candida albicans confirms that Nrg1 is a transcriptional repressor and that Gcn4 is a transcriptional activator. Fungal Genet Biol. 2005;42: 676-683.
3. Munro CA, Selvaggini S, de Bruijn I, Walker L, Lenardon MD, et al. The PKC, HOG and Ca2+ signalling pathways co-ordinately regulate chitin synthesis in Candida albicans. Mol Microbiol. 2007;63:1399-1413.
4. Lohse MB, Johnson AD. Temporal anatomy of an epigenetic switch in cell programming: the white-opaque transition of C. albicans. Mol Microbiol. 2010;78: 331-343.
5. Lenardon MD, Nantel A. Rapid detection of aneuploidy following the generation of mutants in Candida albicans. Methods Mol Biol. 2012;845: 41-49.
