# Supplementary material for: Amplification of TLO Mediator Subunit Genes Facilitate Filamentous Growth in Candida Spp
Source: PLoS Genet. 2016 Oct 14;12(10):e1006373. doi: 10.1371/journal.pgen.1006373 (PMC5065183; doi:10.1371/journal.pgen.1006373)
Supplement: S1 Table — (PDF) [file pgen.1006373.s027.pdf]

S1 Table. Growth conditions tested for phenotypes caused by overexpression of *CaTLOα12*(yLM331)*, CdTLO1* (yLM332) and *HyNT1C* (yLM333)*

**Media**	**Temperature/ Concentration**	**Phenotype (vs. endogenous expression of *CdTLO1*)**
YPD	30°C	Fitness unchanged
YPD	37°C	Fitness unchanged; No wrinkling
YPD + Serum	37°C; 10%	No wrinkling
YPD	42°C	Fitness unchanged
Spider medium	30°C	No wrinkling
YPGalactose + Antimycin A	30°C; 1 µg/mL	Fitness unchanged
YPD+NaCl	30°C; 1.5 M	Fitness unchanged
YPD+Sorbitol	30°C; 1 M	Fitness unchanged
synthetic complete	30°C	Fitness unchanged
YPD+H_2O_2	30°C; 4 mM	Fitness unchanged
YPD+H_2O_2	30°C; 5 mM	Fitness unchanged
YPD+H_2O_2	30°C; 6 mM	Fitness unchanged
YP sucrose (embedded)	30°C; 2% agar	Not hyper-filamentous

* The over-expression strains tested in this table were generated by integrating two copies of a non-tagged *TLO* variant (*CaTLOα12, CdTLO1 or HyNT1C*), driven by a *TDH3* promoter, into the *TLO1* locus in *a tlo1tlo2* double null *C. dubliniensis* strain (yLM125). *HyNT1C* (CaTloα12(1-38)/CdTlo1(39-74)/CaTloα12(75-165)/CdTlo1(199-320) is the stable and *C. dubliniensis* Mediator complex incorporation competent *Ca*Tloα12p/*Cd*Tlo1p hybrid protein as characterized in Fig.3.
