# Supplementary material for: Amplification of TLO Mediator Subunit Genes Facilitate Filamentous Growth in Candida Spp
Source: PLoS Genet. 2016 Oct 14;12(10):e1006373. doi: 10.1371/journal.pgen.1006373 (PMC5065183; doi:10.1371/journal.pgen.1006373)
Supplement: S2 Table — (PDF) [file pgen.1006373.s028.pdf]

S2 Table. List of *C. dubliniensis* strains used in this study

Strain	Parental Strain	Genotype	Ref.
Wü284 (yLM122)		*Candida dubliniensis* Wild-type Isolate	[1]
tlo1Δ/Δ (yLM123)		*tlo1Δ::FRT/ tlo1Δ::FRT*	[2]
tlo2Δ/Δ (yLM124)		*tlo2Δ::FRT/-**	[3]
tloΔΔ (yLM125)		*tlo1Δ::FRT/ tlo1Δ::FRT; tlo2Δ::FRT/-*	[2]
med3Δ/Δ (yLM300)		*med3Δ::FRT/ med3Δ::FRT*	[3]
Tlo1-HA (yLM301)		*TLO1/TLO1::TLO1-3HA-SAT1*	[3]
yLM302	*tloΔΔ*	*tlo2Δ::FRT/- tlo1Δ::FRT/ tlo1Δ::*P_{TDH3}*-CdTLO1-3HA-SAT1*	This study
yLM303	*tloΔΔ*	*tlo2Δ::FRT/- tlo1Δ::FRT/ tlo1Δ::*P_{TDH3}*-CaTLOα12-3HA-SAT1*	This study
yLM304	*tloΔΔ*	*tlo2Δ::FRT/- tlo1Δ::FRT/ tlo1Δ::*P_{TDH3}*-CdTLO1-3HA-SAT1-* P_{TDH3}*-CdTLO1-3HA*	This study
yLM305	*tloΔΔ*	*tlo2Δ::FRT/- tlo1Δ::FRT/ tlo1Δ::*P_{TDH3}*-CaTLOα12-3HA-SAT1-* P_{TDH3}*-CaTLOα12-3HA*	This study
yLM306	*tloΔΔ*	*tlo2Δ::FRT/- tlo1Δ::FRT/ tlo1Δ::*P_{TDH3}*-CaTLOα12-3HA-SAT1-* P_{TDH3}*-CdTLO1-3HA*	This study
yLM307	*tloΔΔ*	*tlo2Δ::FRT/- tlo1Δ::FRT/ tlo1Δ::*P_{TDH3}*-CdTLO1-3HA-SAT1-* P_{TDH3}*-CaTLOα12-3HA*	This study
yLM308	*med3Δ/Δ*	*med3Δ::FRT/med3Δ::FRT TLO1/TLO1::TLO1-3HA-SAT1*	This study
yLM309	*tloΔΔ*	*tlo2Δ::FRT/- tlo1Δ::FRT/ tlo1Δ::*P_{TDH3}*-CaTLOα12-6His3FLAG-SAT1-* P_{TDH3}*-CaTLOα12-6His3FLAG*	This study
yLM310	*tloΔΔ*	*tlo2Δ::FRT/- tlo1Δ::FRT/ tlo1Δ::*P_{TDH3}*-12NT1C-3HA-SAT1*	This study
yLM311	*tloΔΔ*	*tlo2Δ::FRT/- tlo1Δ::FRT/ tlo1Δ::*P_{TDH3}*-(12N-1)-3HA-SAT1*	This study

yLM312	*tloΔΔ*	*tlo2Δ::FRT/- tlo1Δ::FRT/ tlo1Δ::P_{TDH3}-(12N-2)-3HA-SAT1*	This study
yLM313	*tloΔΔ*	*tlo2Δ::FRT/- tlo1Δ::FRT/ tlo1Δ::P_{TDH3}-(12N-3)-3HA-SAT1*	This study
yLM314	*tloΔΔ*	*tlo2Δ::FRT/- tlo1Δ::FRT/ tlo1Δ::P_{TDH3}-(12N-4)-3HA-SAT1*	This study
yLM315	*tloΔΔ*	*tlo2Δ::FRT/- tlo1Δ::FRT/ tlo1Δ::P_{TDH3}-T1N12C-6His3FLAG-SAT1*	This study
yLM316	*tloΔΔ*	*tlo2Δ::FRT/- tlo1Δ::FRT/ tlo1Δ::P_{TDH3}-(TN-1)-6His3FLAG-SAT1*	This study
yLM317	*tloΔΔ*	*tlo2Δ::FRT/- tlo1Δ::FRT/ tlo1Δ::P_{TDH3}-(TN-2)-6His3FLAG-SAT1*	This study
yLM318	*tloΔΔ*	*tlo2Δ::FRT/- tlo1Δ::FRT/ tlo1Δ::P_{TDH3}-(TN-4)-6His3FLAG-SAT1*	This study
yLM319	*tloΔΔ*	*tlo2Δ::FRT/- tlo1Δ::FRT/ tlo1Δ::P_{TDH3}-(TN-5)-6His3FLAG-SAT1*	This study
yLM320	*tloΔΔ*	*tlo2Δ::FRT/- tlo1Δ::FRT/ tlo1Δ::P_{TDH3}-(TN-7)-6His3FLAG-SAT1*	This study
yLM321	*tloΔΔ*	*tlo2Δ::FRT/- tlo1Δ::FRT/ tlo1Δ::P_{TDH3}-12NT1C-6His3FLAG-SAT1*	This study
yLM322	*tloΔΔ*	*tlo2Δ::FRT/- tlo1Δ::FRT/ tlo1Δ::P_{TDH3}-(12N-1)-6His3FLAG-SAT1*	This study
yLM323	*tloΔΔ*	*tlo2Δ::FRT/- tlo1Δ::FRT/ tlo1Δ::P_{TDH3}-(12N-3)-6His3FLAG-SAT1*	This study
yLM324	*tloΔΔ*	*tlo2Δ::FRT/- tlo1Δ::FRT/ tlo1Δ::P_{TDH3}-(12N-4)-6His3FLAG-SAT1*	This study
yLM325	*tloΔΔ*	*tlo2Δ::FRT/- tlo1Δ::FRT/ tlo1Δ::P_{TDH3}-T12H_2-6His3FLAG-SAT1*	This study
yLM326	*tloΔΔ*	*tlo2Δ::FRT/- tlo1Δ::FRT/ tlo1Δ::P_{TDH3}-12TH_2-6His3FLAG-SAT1*	This study
yLM327	*tloΔΔ*	*tlo2Δ::FRT/- tlo1Δ::FRT/ tlo1Δ::P_{TDH3}-CdTLO2-6His3FLAG-SAT1*	This study
yLM328	*tloΔΔ*	*tlo2Δ::FRT/- tlo1Δ::FRT/ tlo1Δ::P_{TDH3}-HyNT1C-3HA-SAT1*	This study
yLM329	*tloΔΔ*	*tlo2Δ::FRT/- tlo1Δ::FRT/ tlo1Δ::P_{TDH3}-HyNT1C-3HA-SAT1- P_{TDH3}-HyNT1C-3HA*	This study

yLM330	*tloΔΔ*	*tlo2Δ::FRT/- tlo1Δ::FRT/ tlo1Δ::P$_{TDH3}$-CdTLO1-3HA-SAT1- P$_{TDH3}$-HyNT1C-3HA*	This study
yLM251		*tlo2Δ::FRT/- tlo1Δ::FRT/ tlo1Δ::P$_{TLO1}$-TLO1-SAT1;*	[4]
yLM252		*tlo2Δ::FRT/- tlo1Δ::FRT/ tlo1Δ::P$_{TLO1}$-TLO1ΔC-SAT1;*	[4]
yLM331	*tloΔΔ*	*tlo2Δ::FRT/- tlo1Δ::FRT/ tlo1Δ::P$_{TDH3}$- CaTLOα12-SAT1-P$_{TDH3}$- CaTLOα12*	This study
yLM332	*tloΔΔ*	*tlo2Δ::FRT/- tlo1Δ::FRT/ tlo1Δ::P$_{TDH3}$-CdTLO1-SAT1-P$_{TDH3}$-CdTLO1*	This study
yLM333	*tloΔΔ*	*tlo2Δ::FRT/- tlo1Δ::FRT/ tlo1Δ::P$_{TDH3}$-HyNT1C-SAT1-P$_{TDH3}$-HyNT1C*	This study
yLM334	*tloΔΔ*	*tlo2Δ::FRT/- tlo1Δ::FRT/ tlo1Δ::P$_{TDH3}$-HyNTΔC-SAT1-P$_{TDH3}$-HyNTΔC*	This study
yLM335 (caTLO *α12*-3HA-1XOE)	Wü284	*TLO1/tlo1Δ::P$_{TDH3}$-CaTLOα12-3HA-SAT1*	This study
yLM336	*med3Δ/Δ*	*med3Δ::FRT/med3Δ::FRT TLO1/tlo1Δ::P$_{TDH3}$-CaTLOα12-3HA-SAT1*	This study
yLM337 (TLO1-3HA-1XOE)	Wü284	*TLO1/tlo1Δ::P$_{TDH3}$-CdTLO1-3HA-SAT1*	This study
yLM338	*med3Δ/Δ*	*med3Δ::FRT/med3Δ::FRT TLO1/tlo1Δ::P$_{TDH3}$-CdTLO1-3HA-SAT1*	This study
yLM339 (TLO2-3HA-1XOE)	Wü284	*TLO1/tlo1Δ::P$_{TDH3}$-CdTLO2-3HA-SAT1*	This study
yLM340	*med3Δ/Δ*	*med3Δ::FRT/med3Δ::FRT TLO1/tlo1Δ::P$_{TDH3}$-CdTLO2-3HA-SAT1*	This study
yLM341	Wü284	*TLO1/tlo1Δ::P$_{TDH3}$-HyNT1C-3HA-SAT1*	This study
yLM342	*med3Δ/Δ*	*med3Δ::FRT/med3Δ::FRT TLO1/tlo1Δ::P$_{TDH3}$-HyNT1C-3HA-SAT1*	This study
yLM343	Wü284	*TLO1/tlo1Δ::P$_{TDH3}$-CdTLO2-3HA-SAT1***(SW**)**	This study
yLM344 (TLO2-	Wü284	*TLO1/ tlo1Δ::P$_{TDH3}$-CdTLO2-3HA-SAT1- P$_{TDH3}$-CdTLO2-3HA*	This study

3HA-2XOE)			
yLM345	Wü284	*TLO1/tlo1Δ::P_{TDH3}-CdTLO2-3HA-SAT1- P_{TDH3}-CdTLO2-3HA* **(SW)**	This study
yLM346 (TLO2 -2XOE)	Wü284	*TLO1/tlo1Δ::P_{TDH3}-CdTLO2-SAT1- P_{TDH3}-CdTLO2*	This study
yLM347	Wü284	*TLO1/tlo1Δ::P_{TDH3}-CdTLO2-SAT1- P_{TDH3}-CdTLO2* **(SW)**	This study
yLM348	Wü284	*TLO1/tlo1Δ::P_{CaACT1}-CdTLO2-3HA-SAT1*	This study
yLM349	Wü284	*TLO1/tlo1Δ::P_{CaACT1}-CdTLO2-3HA-SAT1***(SW)**	This study
yLM350	Wü284	*TLO1/tlo1Δ::P_{TDH3}-T1NT2C-3HA-SAT1*	This study
yLM351	Wü284	*TLO1/tlo1Δ::P_{TDH3}-T2NT1C-3HA-SAT1*	This study
yLM352	Wü284	*TLO1/tlo1Δ::P_{TDH3}-HyNT2C-3HA-SAT1*	This study
yLM353	Wü284	*TLO1/tlo1Δ::P_{TDH3}-CaTLOα12-GFP-SAT1*	This study
yLM354	*med3Δ/Δ*	*med3Δ::FRT/med3Δ::FRT TLO1/tlo1Δ::P_{TDH3}-CaTLOα12-GFP-SAT1*	This study
yLM355	Wü284	*TLO1/tlo1Δ::P_{TDH3}-CdTLO1-GFP-SAT1*	This study
yLM356	*med3Δ/Δ*	*med3Δ::FRT/med3Δ::FRT TLO1/tlo1Δ::P_{TDH3}-CdTLO1-GFP-SAT1*	This study
yLM357	Wü284	*TLO1/tlo1Δ::P_{TDH3}-CdTLO2-GFP-SAT1*	This study
yLM358	*med3Δ/Δ*	*med3Δ::FRT/med3Δ::FRT TLO1/tlo1Δ::P_{TDH3}-CdTLO2-GFP-SAT1*	This study
yLM359	Wü284	*MED3/MED3::MED3-GFP-SAT1*	This study
yLM360	*tlo1Δ/Δ*	*tlo1Δ::FRT/ tlo1Δ::FRT MED3/MED3::MED3-GFP-SAT1*	This study
yLM361	*tloΔ/Δ*	*tlo1Δ::FRT/ tlo1Δ::FRT tlo2Δ::FRT/-MED3/MED3::MED3-GFP-SAT1*	This study
yLM362 (NLS-GFP-3HA-1XOE)	Wü284	*TLO1/tlo1Δ::P_{TDH3}-NLS-GFP-3HA-SAT1*	This study
yLM363 (NLS-	Wü284	*TLO1/tlo1Δ::P_{TDH3}-NLS-GFP- CaTLOα12-3HA-SAT1*	This study

GFP-Ca TLOα12-3HA-1XOE)			
yLM364 (NLS-GFP-Ca TLOα12 TAD-3HA-1XOE)	Wü284	*TLO1/tlo1Δ::P_{TDH3}-NLS-GFP- CaTLOα12TAD-3HA-SAT1*	This study
yLM365	Wü284	*TLO1/tlo1Δ::P_{TDH3}-NLS-GFP- CdTLO1-3HA-SAT1*	This study
yLM366	Wü284	*TLO1/tlo1Δ::P_{TDH3}-NLS-GFP- CdTLO1TAD-3HA-SAT1*	This study
yLM367 (NLS-GFP-TLO2-3HA-1XOE)	Wü284	*TLO1/tlo1Δ::P_{TDH3}-NLS-GFP- CdTLO2-3HA-SAT1*	This study
yLM368 (NLS-GFP-TLO2 TAD-3HA-1XOE)	Wü284	TLO1/tlo1Δ::P_{TDH3}-NLS-GFP- CdTLO2TAD-3HA-SAT1	This study
yLM369 (NLS-GFP-TLO2-1X OE)	Wü284	*TLO1/tlo1Δ::P_{TDH3}-NLS-GFP-CdTLO2-SAT1*	This study
yLM370	*tlo1Δ/Δ*	*tlo1Δ::FRT/tlo1Δ::P_{TDH3}-NLS-GFP-3HA-SAT1*	This study
yLM371	*tlo1Δ/Δ*	*tlo1Δ::FRT/tlo1Δ::P_{TDH3}-NLS-GFP- CaTLOα12-3HA-SAT1*	This study
yLM372 (NLS-GFP-Ca TLOα12 TAD-3HA-1XOE)	*tlo1Δ/Δ*	*tlo1Δ::FRT/tlo1Δ::P_{TDH3}-NLS-GFP- CaTLOα12TAD-3HA-SAT1*	This study
yLM373	*tlo1Δ/Δ*	*tlo1Δ::FRT/tlo1Δ::P_{TDH3}-NLS-GFP- CdTLO1-3HA-*	This study

		SAT1	
yLM374	*tlo1Δ/Δ*	*tlo1Δ::FRT/tlo1Δ::P_{TDH3}-NLS-GFP- CdTLO1TAD-3HA-SAT1*	This study
yLM375	*tlo1Δ/Δ*	*tlo1Δ::FRT/tlo1Δ::P_{TDH3}-NLS-GFP- CdTLO2-3HA-SAT1*	This study
yLM376 (NLS-GFP-CdTLO2TAD-3HA-1XOE)	*tlo1Δ/Δ*	*tlo1Δ::FRT/tlo1Δ::P_{TDH3}-NLS-GFP- CdTLO2TAD-3HA-SAT1*	This study
yLM377	*med3Δ/Δ*	*med3Δ::FRT/med3Δ::FRT TLO1/tlo1Δ::P_{TDH3}-NLS-GFP-3HA-SAT1*	This study
yLM378	*med3Δ/Δ*	*med3Δ::FRT/med3Δ::FRT TLO1/tlo1Δ::P_{TDH3}-NLS-GFP- CaTLOα12-3HA-SAT1*	This study
yLM379	*med3Δ/Δ*	*med3Δ::FRT/med3Δ::FRT TLO1/tlo1Δ::P_{TDH3}-NLS-GFP- CaTLOα12TAD-3HA-SAT1*	This study
yLM380	*med3Δ/Δ*	*med3Δ::FRT/med3Δ::FRT TLO1/tlo1Δ::P_{TDH3}-NLS-GFP- CdTLO1-3HA-SAT1*	This study
yLM381	*med3Δ/Δ*	*med3Δ::FRT/med3Δ::FRT TLO1/tlo1Δ::P_{TDH3}-NLS-GFP- CdTLO1TAD-3HA-SAT1*	This study
yLM382	*med3Δ/Δ*	*med3Δ::FRT/med3Δ::FRT TLO1/tlo1Δ::P_{TDH3}-NLS-GFP- CdTLO2-3HA-SAT1*	This study
yLM383	*med3Δ/Δ*	*med3Δ::FRT/med3Δ::FRT TLO1/tlo1Δ::P_{TDH3}-NLS-GFP- CdTLO2TAD-3HA-SAT1*	This study
yLM384	Wü284	*TLO1/tlo1Δ::P_{TDH3}-GFP-CdTLO2-3HA-SAT1*	This study
yLM385	Wü284	*TLO1/tlo1Δ::P_{TDH3}-GFP- CdTLO2-3HA-SAT1***(SW)**	This study
yLM386	Wü284	*TLO2::TLO2-3HA-SAT1/-*	This study
yLM387	*tlo1Δ/Δ*	*tlo1Δ::FRT/tlo1Δ::FRT TLO2::TLO2-3HA-SAT1/-*	This study
Tlo1-HF (yLM415)	Wü284	*TLO1/TLO1::TLO1-6His3FLAG-SAT1*	[3]
yLM416	Wü284	*TLO1/tlo1Δ::P_{TDH3}-CdTLO2-6HisFLAG-SAT1-P_{TDH3}-CdTLO2-6HisFLAG*	This study
Wü284 ENO1p-CdTLO2	Wü284	*TLO2::pNAT-ENO1-TLO2 /-*	This study

CD36		*Candida dubliniensis* Wild-type Isolate	[5]
CD36 ENO1p-CdTLO2	*CD36*	*TLO2::pNAT-ENO1-TLO2 /TLO2*	This study
AV5		*Candida dubliniensis* Wild-type Isolate	[6]
AV5 ENO1p-CdTLO2	*AV5*	*TLO2::pNAT-ENO1-TLO2 /TLO2*	This study

* There is only one intact allele of *TLO2* present in all *C. dubliniensis* strains used in this study due to a chromosomal truncation event happened in Wü284, the common parental wild type strain.

SW stands for strains with 'S**uper-**W**rinkled' colony morphology.

1. Morschhauser J, Ruhnke M, Michel S, Hacker J. Identification of CARE-2-negative Candida albicans isolates as Candida dubliniensis. Mycoses 1999;42: 29-32.

2. Jackson AP, Gamble JA, Yeomans T, Moran GP, Saunders D, et al. Comparative genomics of the fungal pathogens Candida dubliniensis and Candida albicans. Genome Res. 2009;19: 2231-2244.

3. Haran J, Boyle H, Hokamp K, Yeomans T, Liu Z, et al. Telomeric ORFs (TLOs) in Candida spp. Encode Mediator Subunits That Regulate Distinct Virulence Traits. PLoS Genet. 2015;10: e1004658.

4. Liu Z, Myers LC. Fungal mediator tail subunits contain classical transcriptional activation domains. Mol Cell Biol. 2015;35:1363-75.

5. Sullivan DJ, Westerneng TJ, Haynes KA, Bennett DE, Coleman DC. Candida dubliniensis sp. nov.: phenotypic and molecular characterization of a novel species associated with oral candidosis in HIV-infected individuals. Microbiology 1995;141:1507–1521.

6. McManus BA, Sullivan DJ, Moran GP, d'Enfert C, Bougnoux ME, Nunn MA, Coleman DC.Genetic differences between avian and human isolates of Candida dubliniensis. Emerg Infect Dis. 2009;15:1467-70.
