# Supplementary material for: Amplification of TLO Mediator Subunit Genes Facilitate Filamentous Growth in Candida Spp
Source: PLoS Genet. 2016 Oct 14;12(10):e1006373. doi: 10.1371/journal.pgen.1006373 (PMC5065183; doi:10.1371/journal.pgen.1006373)
Supplement: S3 Table — (PDF) [file pgen.1006373.s029.pdf]

S3 Table. List of *C. albicans* strains used in this study

Strain	Parental Strain	Genotype	Ref.
BWP17		MTLa/α *ura3Δ::λimm*^{434}*/ura3Δ::λimm*^{434} *his1Δ::hisG/his1Δ::hisG arg4Δ::hisG/arg4Δ::hisG*	[1]
SN152		*arg4/arg4 leu2/leu2 his1/his1 URA3/ura3::imm434 IRO1/iro1::imm434*	[2]
cTTR01		*arg4/arg4 URA3/ura3::imm434 IRO1/iro1::imm434 med15Δ::HIS1/med15Δ::LEU2*	[3]
cTTR03		*MTLa/α ura3Δ::λimm*^{434}*/ura3Δ::λimm*^{434} *his1Δ::hisG/his1Δ::hisG arg4Δ::hisG/arg4Δ::hisG MED8::MED8-6His3FLAG-SAT1/ MED8::MED8-6His3FLAG-HIS1 MED3/MED3::MED3-3HA-ARG4*	[3]
AZC34		*arg4/arg4 URA3/ura3::imm434 IRO1/iro1::imm434 med16Δ::HIS1/med16Δ::LEU2*	[3]
yLM388	SN152	*arg4/arg4 leu2/leu2 his1/his1 URA3/ura3::imm434 IRO1/iro1::imm434 CaTLOα12/ CaTLOα12:: CaTLOα12-3HA-SAT1*	This study
yLM389	SN152	*arg4/arg4 leu2/leu2 his1/his1 URA3/ura3::imm434 IRO1/iro1::imm434 RPS10/rps10Δ::*P_{ACT1}*-CaTLOα12-3HA-SAT1*	This study
yLM390	SN152	*arg4/arg4 leu2/leu2 his1/his1 URA3/ura3::imm434 IRO1/iro1::imm434 RPS10/rps10Δ::*P_{ACT1}*-CdTLO1-3HA-SAT1*	This study
yLM391	SN152	*arg4/arg4 leu2/leu2 his1/his1 URA3/ura3::imm434 IRO1/iro1::imm434 CaTLOα34/CaTLOα34:: CaTLOα34-3HA-SAT1*	This study
yLM116	SN152	*arg4/arg4 leu2/leu2 his1/his1 URA3/ura3::imm434 IRO1/iro1::imm434 med3Δ::HIS1/MED3*	This study
yLM119	yLM116	*arg4/arg4 leu2/leu2 his1/his1 URA3/ura3::imm434 IRO1/iro1::imm434 med3Δ::HIS1/med3Δ::LEU2*	This study
yLM120	yLM119	*arg4/arg4 leu2/leu2 his1/his1 URA3/ura3::imm434 IRO1/iro1::imm434 med3Δ::HIS1/med3Δ::MED3-ARG4*	This study
yLM392	yLM119	*arg4/arg4 leu2/leu2 his1/his1 URA3/ura3::imm434 IRO1/iro1::imm434 med3Δ::HIS1/med3Δ::LEU2 CaTLOα34/CaTLOα34:: CaTLOα34-3HA-SAT1*	This study
yLM393	BWP17	MTLa/α *ura3Δ::λimm*^{434}*/ura3Δ::λimm*^{434} *his1Δ::hisG/his1Δ::hisG arg4Δ::hisG/arg4Δ::hisG*	This study

		RPS10/rps10Δ::P_{ACT1}-CaTLOα12-3HA-SAT1	
yLM394	BWP17	MTLa/α *ura3Δ::λimm^{434}/ura3Δ::λimm^{434} his1Δ::hisG/his1Δ::hisG arg4Δ::hisG/arg4Δ::hisG RPS10/rps10Δ::P_{ACT1}-CdTLO1-3HA-SAT1*	This study
yLM395	SN152	*arg4/arg4 leu2/leu2 his1/his1 URA3/ura3::imm434 IRO1/iro1::imm434 RPS10/rps10Δ::P_{ACT1}-12NT1C-3HA-SAT1*	This study
yLM396	SN152	*arg4/arg4 leu2/leu2 his1/his1 URA3/ura3::imm434 IRO1/iro1::imm434 RPS10/rps10Δ::P_{ACT1}-(12N-1)-3HA-SAT1*	This study
yLM397	SN152	*arg4/arg4 leu2/leu2 his1/his1 URA3/ura3::imm434 IRO1/iro1::imm434 RPS10/rps10Δ::P_{ACT1}-(12N-2)-3HA-SAT1*	This study
yLM398	SN152	*arg4/arg4 leu2/leu2 his1/his1 URA3/ura3::imm434 IRO1/iro1::imm434 RPS10/rps10Δ::P_{ACT1}-(12N-3)-3HA-SAT1*	This study
yLM399	SN152	*arg4/arg4 leu2/leu2 his1/his1 URA3/ura3::imm434 IRO1/iro1::imm434 RPS10/rps10Δ::P_{ACT1}-(12N-4)-3HA-SAT1*	This study
yLM400	SN152	*arg4/arg4 leu2/leu2 his1/his1 URA3/ura3::imm434 IRO1/iro1::imm434 RPS10/rps10Δ::P_{ACT1}-T1N12C-3HA-SAT1*	This study
yLM401	SN152	*arg4/arg4 leu2/leu2 his1/his1 URA3/ura3::imm434 IRO1/iro1::imm434 RPS10/rps10Δ::P_{ACT1}-(TN-1)-3HA-SAT1*	This study
yLM402	SN152	*arg4/arg4 leu2/leu2 his1/his1 URA3/ura3::imm434 IRO1/iro1::imm434 RPS10/rps10Δ::P_{ACT1}-(TN-2)-3HA-SAT1*	This study
yLM403	SN152	*arg4/arg4 leu2/leu2 his1/his1 URA3/ura3::imm434 IRO1/iro1::imm434 RPS10/rps10Δ::P_{ACT1}-(TN-3)-3HA-SAT1*	This study
yLM404	SN152	*arg4/arg4 leu2/leu2 his1/his1 URA3/ura3::imm434 IRO1/iro1::imm434 RPS10/rps10Δ::P_{ACT1}-(TN-4)-3HA-SAT1*	This study
yLM405	SN152	*arg4/arg4 leu2/leu2 his1/his1 URA3/ura3::imm434 IRO1/iro1::imm434 RPS10/rps10Δ::P_{ACT1}-(TN-5)-3HA-SAT1*	This study
yLM406	SN152	*arg4/arg4 leu2/leu2 his1/his1 URA3/ura3::imm434 IRO1/iro1::imm434 RPS10/rps10Δ::P_{ACT1}-(TN-6)-3HA-SAT1*	This study

yLM407	SN152	*arg4/arg4 leu2/leu2 his1/his1 URA3/ura3::imm434 IRO1/iro1::imm434 RPS10/rps10Δ::P_{ACT1}-(TN-7)-3HA-SAT1*	This study
yLM408	SN152	*arg4/arg4 leu2/leu2 his1/his1 URA3/ura3::imm434 IRO1/iro1::imm434 RPS10/rps10Δ::P_{ACT1}-HyNT1C-3HA-SAT1*	This study
yLM258		*arg4/arg4 leu2/leu2 his1/his1 URA3/ura3 ::imm434 IRO1/iro1 ::imm434 med15Δ::HIS1/med15Δ::LEU2 MED8/MED8::MED8-6His3FLAG-SAT1 MED3/MED3-3HA-HIS1*	[4]
yLM409	SN152	*arg4/arg4 leu2/leu2 his1/his1 URA3/ura3::imm434 IRO1/iro1::imm434 RPS10/rps10Δ::URA3-P_{ACT1}-CaTLOα12-GFP-SAT1*	This study
yLM410	yLM119	*arg4/arg4 leu2/leu2 his1/his1 URA3/ura3::imm434 IRO1/iro1::imm434 med3Δ::HIS1/med3Δ::LEU2 RPS10/rps10Δ::URA3-P_{ACT1}-CaTLOα12-GFP-SAT1*	This study
yLM411	cTTR01	*arg4/arg4 URA3/ura3::imm434 IRO1/iro1::imm434 med15Δ::HIS1/med15Δ::LEU2 RPS10/rps10Δ::URA3-P_{ACT1}-CaTLOα12-GFP-SAT1*	This study
yLM412	AZC34	*arg4/arg4 URA3/ura3::imm434 IRO1/iro1::imm434 med16Δ::HIS1/med16Δ::LEU RPS10/rps10Δ::URA3-P_{ACT1}-CaTLOα12-GFP-SAT1*	This study
yLM413	cRC106	*ura3Δ::λimm^{434}/ura3Δ::λimm^{434} ade2::hisG/ade2::hisG::[pOPlacZ] RPS10/rps10Δ::URA3-P_{MAL}-LEXADBD-CdTLO2*	This study
yLM414	cRC106	*ura3Δ::λimm^{434}/ura3Δ::λimm^{434} ade2::hisG/ade2::hisG::[pOPlacZ] RPS10/rps10Δ::URA3-P_{MAL}-LEXADBD-CdTLO2ΔC*	This study

1. Wilson RB, Davis D, Mitchell AP. Rapid hypothesis testing with Candida albicans through gene disruption with short homology regions. J Bacteriol 1999;181: 1868-1874.
2. Noble SM, Johnson AD. Strains and strategies for large-scale gene deletion studies of the diploid human fungal pathogen Candida albicans. Eukaryot Cell 2005;4: 298-309.
3. Zhang A, Liu Z, Myers LC. Differential regulation of white-opaque switching by individual subunits of Candida albicans mediator. Eukaryot Cell 2013;12: 1293-1304.
4. Willger SD, Liu Z, Olarte RA, Adamo ME, Stajich JE, Myers LC, Kettenbach AN, Hogan DA. Analysis of the Candida albicans Phosphoproteome. Eukaryot Cell 2015;14:474-85.
