# Supplementary material for: Amplification of TLO Mediator Subunit Genes Facilitate Filamentous Growth in Candida Spp
Source: PLoS Genet. 2016 Oct 14;12(10):e1006373. doi: 10.1371/journal.pgen.1006373 (PMC5065183; doi:10.1371/journal.pgen.1006373)
Supplement: S4 Table — (PDF) [file pgen.1006373.s030.pdf]

S4 Table. List of plasmids used in this study

Plasmid name	Description	Enzymes*	Checking primers	
			5'	3'
pFA-3HA-SAT1-TLO1-3'	Intermediate construct			
pFA-P$_{TLO1}$- TLO1-3HA-SAT1	Intermediate construct			
pFA-P$_{TDH3}$- TLO1-3HA$_{1X}$	Vector for integrating 1 copy of *P$_{TDH3}$*-driven *TLO1-3HA* to *CdTLO1* locus with *SAT1* marker in *tloΔΔ*	HindIII/SacII	ZL288/ZL307	KPP63/ZL289
pFA-P$_{TDH3}$-TLOα12-3HA$_{1X}$	Vector for integrating 1 copy of *P$_{TDH3}$*-driven *TLOα12-3HA* to *CdTLO1* locus with *SAT1* marker in *tloΔΔ*	HindIII/SacII	ZL288/ZL307	KPP63/ZL289
pFA-P$_{TDH3}$- TLO1-3HA$_{2X}$	Vector for integrating 2 copies of *P$_{TDH3}$*-driven *TLO1-3HA* to *CdTLO1* locus with *SAT1* marker in *tloΔΔ*	HindIII/SacII	ZL288/ZL307	ZL302/ZL289
pFA-P$_{TDH3}$-TLOα12-3HA$_{2X}$	Vector for integrating 2 copies of *P$_{TDH3}$*-driven *TLOα12-3HA* to *CdTLO1* locus with *SAT1* marker in *tloΔΔ*	HindIII/SacII	ZL288/ZL307	ZL302/ZL289
pFA-TLO1-3HA/TLOα12-3HA	Vector for co-expressing *TLO1-3HA* and *TLOα12-3HA* from *CdTLO1* locus in *tloΔΔ*	HindIII/SacII	ZL288/ZL307	ZL302/ZL289
pFA-TLOα12-3HA/TLO1-3HA	Vector for co-expressing *TLO1-3HA* and *TLOα12-3HA* from *CdTLO1* locus in *tloΔΔ*	HindIII/SacII	ZL288/ZL307	ZL302/ZL289
pFA-P$_{TDH3}$-TLO1$_{2X}$	Vector for integrating 2 copies of *P$_{TDH3}$*-driven *TLO1* to *CdTLO1* locus with *SAT1* marker in *tloΔΔ*	HindIII/SacII	ZL288/ZL307	ZL302/ZL289
pFA-P$_{TDH3}$-TLOα12$_{2X}$	Vector for integrating 2 copies of *P$_{TDH3}$*-driven *TLOα12* to *CdTLO1* locus with *SAT1* marker in *tloΔΔ*	HindIII/SacII	ZL288/ZL307	ZL302/ZL289
pACT1- TLO1-3HA-URA3	Vector for integrating 1 copy of *P$_{ACT1}$*-driven *TLO1-3HA* to *RPS10* locus with *URA3* marker in *C. albicans* BWP17	NcoI/AgeI	ZL093/ZL174	ZL313/ZL094

pACT1-TLOα12-3HA-URA3	Vector for integrating 1 copy of P_{ACT1}-driven *TLOα12-3HA* to *RPS10* locus with *URA3* marker in *C. albicans* BWP17	NcoI/AgeI	ZL093/ZL174	ZL313/ZL094
pACT1- TLO1-3HA-SAT1	Vector for integrating 1 copy of P_{ACT1}-driven *TLO1-3HA* to *RPS10* locus with *SAT1* marker in *C. albicans* SN152	NcoI/AgeI	ZL093/KPP63	ZL313/ZL094
pACT1-TLOα12-3HA-SAT1	Vector for integrating 1 copy of P_{ACT1}-driven *TLOα12-3HA* to *RPS10* locus with *SAT1* marker in *C. albicans* SN152	NcoI/AgeI	ZL093/KPP63	ZL313/ZL094
plac-LacZ-SAT1	SAT1 marker 'donor' for C. albicans overexpression vectors; derived from *plac-LacZ* [4]			
pACT1-(12N-1)-3HA-SAT1	Vector for overexpression of *(12N-1)-3HA* chimeric gene in *C. albicans* SN152	NcoI/AgeI	ZL093/KPP63	ZL313/ZL094
pACT1-(12N-2)-3HA-SAT1	Vector for overexpression of *(12N-2)-3HA* chimeric gene in *C. albicans* SN152	NcoI/AgeI	ZL093/KPP63	ZL313/ZL094
pACT1-(12N-3)-3HA-SAT1	Vector for overexpression of *(12N-3)-3HA* chimeric gene in *C. albicans* SN152	NcoI/AgeI	ZL093/KPP63	ZL313/ZL094
pACT1-(12N-4)-3HA-SAT1	Vector for overexpression of *(12N-4)-3HA* chimeric gene in *C. albicans* SN152	NcoI/AgeI	ZL093/KPP63	ZL313/ZL094
pACT1-(TN-1)-3HA-SAT1	Vector for overexpression of *(TN-1)-3HA* chimeric gene in *C. albicans* SN152	NcoI/AgeI	ZL093/KPP63	ZL313/ZL094
pACT1-(TN-2)-3HA-SAT1	Vector for overexpression of *(TN-2)-3HA* chimeric gene in *C. albicans* SN152	NcoI/AgeI	ZL093/KPP63	ZL313/ZL094
pACT1-(TN-3)-3HA-SAT1	Vector for overexpression of *(TN-3)-3HA* chimeric gene in *C. albicans* SN152	NcoI/AgeI	ZL093/KPP63	ZL313/ZL094
pACT1-(TN-4)-3HA-SAT1	Vector for overexpression of *(TN-4)-3HA* chimeric gene in *C. albicans* SN152	NcoI/AgeI	ZL093/KPP63	ZL313/ZL094

pACT1-(TN-5)-3HA-SAT1	Vector for overexpression of *(TN-5)-3HA* chimeric gene in *C. albicans* SN152	NcoI/AgeI	ZL093/KPP63	ZL313/ZL094
pFA-P_{TDH3}- (12N-1)-3HA	Vector for overexpression of *(12N-1)-3HA* chimeric gene in *C. dubliniensis tloΔΔ*	HindIII/SacII	ZL288/ZL307	KPP63/ZL289
pFA-P_{TDH3}- (12N-2)-3HA	Vector for overexpression of *(12N-2)-3HA* chimeric gene in *C. dubliniensis tloΔΔ*	HindIII/SacII	ZL288/ZL307	KPP63/ZL289
pFA-P_{TDH3}- (12N-3)-3HA	Vector for overexpression of *(12N-3)-3HA* chimeric gene in *C. dubliniensis tloΔΔ*	HindIII/SacII	ZL288/ZL307	KPP63/ZL289
pFA-P_{TDH3}- (12N-4)-3HA	Vector for overexpression of *(12N-4)-3HA* chimeric gene in *C. dubliniensis tloΔΔ*	HindIII/SacII	ZL288/ZL307	KPP63/ZL289
pFA-P_{TDH3}- (TN-1)-3HA	Intermediate construct			
pFA-P_{TDH3}- (TN-2)-3HA	Intermediate construct			
pFA-P_{TDH3}- (TN-4)-3HA	Intermediate construct			
pFA-P_{TDH3}- (TN-5)-3HA	Intermediate construct			
pFA-P_{TDH3}-T1N12C-3HA	Intermediate construct			
pACT1-T1N12C-3HA-SAT1	Vector for overexpression of *T1N12C-3HA* chimeric gene in *C. albicans* SN152	NcoI/AgeI	ZL093/KPP63	ZL313/ZL094
pFA-P_{TDH3}- (TN-6)-3HA	Intermediate construct			
pACT1-(TN-6)-3HA-SAT1	Vector for overexpression of *(TN-6)-3HA* chimeric gene in *C. albicans* SN152	NcoI/AgeI	ZL093/KPP63	ZL313/ZL094
pFA-P_{TDH3}- (TN-7)-3HA	Intermediate construct			

pACT1-(TN-7)-3HA-SAT1	Vector for overexpression of *(TN-7)-3HA* chimeric gene in *C. albicans* SN152	NcoI/AgeI	ZL093/KPP63	ZL313/ZL094
pFA-P_{TDH3}-12NT1C-3HA	Vector for overexpression of *12NT1C-3HA* chimeric gene in *C. dubliniensis tloΔΔ*	HindIII/SacII	ZL288/ZL307	KPP63/ZL289
pACT1-12NT1C-3HA-SAT1	Vector for overexpression of *12NT1C-3HA* chimeric gene in *C. albicans* SN152	NcoI/AgeI	ZL093/KPP63	ZL313/ZL094
pFA-P_{TDH3}- T12H_2-3HA	Intermediate construct			
pFA-P_{TDH3}- 12TH_2-3HA	Intermediate construct			
pFA-P_{TDH3}-HyNT1C-$3HA_{1X}$	Vector for integrating 1 copy of *P_{TDH3}*-driven *HyNT1C-3HA* to *CdTLO1* locus with *SAT1* marker in *tloΔΔ*	HindIII/SacII	ZL288/ZL307	KPP63/ZL289
pFA-P_{TDH3}-HyNT1C-$3HA_{2X}$	Vector for integrating 2 copies of *P_{TDH3}*-driven *HyNT1C-3HA* to *CdTLO1* locus with *SAT1* marker in *tloΔΔ*	HindIII/SacII	ZL288/ZL307	ZL302/ZL289
pACT1-HyNT1C-3HA-SAT1	Vector for overexpression of *HyNT1C-3HA* chimeric gene in *C. albicans* SN152	NcoI/AgeI	ZL093/KPP63	ZL313/ZL094
pFA-TLO1-3HA/HyNT1C-3HA	Vector for co-expressing *TLO1-3HA* and *HyNT1C-3HA* from *TLO1* locus in *tloΔΔ*	HindIII/SacII	ZL288/ZL307	ZL302/ZL289
pFA-P_{TDH3}-HyNΔC-$3HA_{1X}$	Vector for integrating 1 copy of *P_{TDH3}*-driven *HyNΔC-3HA* to *CdTLO1* locus with *SAT1* marker in *tloΔΔ*	HindIII/SacII	ZL288/ZL307	KPP63/ZL289
pFA-P_{TDH3}-HyNΔC-$3HA_{2X}$	Vector for integrating 1 copies of *P_{TDH3}*-driven *HyNΔC-3HA* to *CdTLO1* locus with *SAT1* marker in *tloΔΔ*	HindIII/SacII	ZL288/ZL307	ZL302/ZL289
pFA-P_{TDH3}-$HyNT1C_{1X}$	Vector for integrating 1 copy of *P_{TDH3}*-driven *HyNT1C* to *CdTLO1* locus with *SAT1* marker in *tloΔΔ*	HindIII/SacII	ZL288/ZL307	KPP63/ZL289

pFA-P$_{TDH3}$-HyNT1C$_{2X}$	Vector for integrating 2 copies of *P$_{TDH3}$*-driven *HyNT1C* to *CdTLO1* locus with *SAT1* marker in *tloΔΔ*	HindIII/SacII	ZL288/ZL307	ZL302/ZL289
pFA-P$_{TDH3}$-HyNΔC$_{1X}$	Vector for integrating 1 copy of *P$_{TDH3}$*-driven *HyNΔC* to *CdTLO1* locus with *SAT1* marker in *tloΔΔ*	HindIII/SacII	ZL288/ZL307	KPP63/ZL289
pFA-P$_{TDH3}$-HyNΔC$_{2X}$	Vector for integrating 1 copies of *P$_{TDH3}$*-driven *HyNΔC* to *CdTLO1* locus with *SAT1* marker in *tloΔΔ*	HindIII/SacII	ZL288/ZL307	ZL302/ZL289
pFA-P$_{TDH3}$-T1NT2C-3HA	Intermediate construct			
pFA-P$_{TDH3}$-T2NT1C-3HA	Intermediate construct			
pFA-P$_{TDH3}$-HyNT2C-3HA	Intermediate construct			
pFA-P$_{TDH3}$-TLOα12-6His3FLAG$_{2X}$	Vector for expression of *TLOα12-6His3FLAG* in *C. dubliniensis tloΔΔ*	HindIII/SacII	ZL288/ZL307	ZL302/ZL289
pFA-P$_{TDH3}$-T1N12C-6His3FLAG	Vector for expression of *T1N12C-6His3FLAG* chimeric gene in *C. dubliniensis tloΔΔ*	HindIII/SacII	ZL288/ZL307	KPP63/ZL289
pFA-P$_{TDH3}$- (TN-1)-6His3FLAG	Vector for expression of *(TN-1)-6His3FLAG* chimeric gene in *C. dubliniensis tloΔΔ*	HindIII/SacII	ZL288/ZL307	KPP63/ZL289
pFA-P$_{TDH3}$- (TN-2)-6His3FLAG	Vector for expression of *(TN-2)-6His3FLAG* chimeric gene in *C. dubliniensis tloΔΔ*	HindIII/SacII	ZL288/ZL307	KPP63/ZL289
pFA-P$_{TDH3}$- (TN-4)-6His3FLAG	Vector for expression of *(TN-4)-6His3FLAG* chimeric gene in *C. dubliniensis tloΔΔ*	HindIII/SacII	ZL288/ZL307	KPP63/ZL289
pFA-P$_{TDH3}$- (TN-5)-6His3FLAG	Vector for expression of *(TN-5)-6His3FLAG* chimeric gene in *C. dubliniensis tloΔΔ*	HindIII/SacII	ZL288/ZL307	KPP63/ZL289
pFA-P$_{TDH3}$- (TN-7)-6His3FLAG	Vector for expression of *(TN-7)-6His3FLAG* chimeric gene in *C. dubliniensis tloΔΔ*	HindIII/SacII	ZL288/ZL307	KPP63/ZL289

pFA-P_{TDH3}*-12NT1C-6His3FLAG*	Vector for expression of *12NT1C-6His3FLAG* chimeric gene in *C. dubliniensis tloΔΔ*	HindIII/SacII	ZL288/ZL307	KPP63/ZL289
pFA-P_{TDH3}*-(12N-1)-6His3FLAG*	Vector for expression of *(12N-1)-6His3FLAG* chimeric gene in *C. dubliniensis tloΔΔ*	HindIII/SacII	ZL288/ZL307	KPP63/ZL289
pFA-P_{TDH3}*-(12N-3)-6His3FLAG*	Vector for expression of *(12N-3)-6His3FLAG* chimeric gene in *C. dubliniensis tloΔΔ*	HindIII/SacII	ZL288/ZL307	KPP63/ZL289
pFA-P_{TDH3}*-(12N-4)-6His3FLAG*	Vector for expression of *(12N-4)-6His3FLAG* chimeric gene in *C. dubliniensis tloΔΔ*	HindIII/SacII	ZL288/ZL307	KPP63/ZL289
pFA-P_{TDH3}*- T12*H_2*-6His3FLAG*	Vector for expression of *T12H2-6His3FLAG* chimeric gene in *C. dubliniensis tloΔΔ*	HindIII/SacII	ZL288/ZL307	KPP63/ZL289
pFA-P_{TDH3}*- 12T*H_2*-6His3FLAG*	Vector for expression of *12T*H_2*-6His3FLAG* chimeric gene in *C. dubliniensis tloΔΔ*	HindIII/SacII	ZL288/ZL307	KPP63/ZL289
pFA-P_{TDH3}*- TLO2-6His3FLAG*$_{1X}$	Vector for expression of *TLO2-6His3FLAG* chimeric gene in *C. dubliniensis tloΔΔ*	HindIII/SacII	ZL288/ZL307	KPP63/ZL289
pFA-P_{TDH3}*- TLO2-3HA*$_{1X}$	Intermediate construct			
pFA-P_{TDH3}*- TLO1-GFP*	Intermediate construct			
pFA-P_{TDH3}*-TLOα12-GFP*	Intermediate construct			
EXpFA-P_{TDH3}*-TLO1-3HA*$_{1X}$	Vector for overexpression of *TLO1-3HA* in *C. dubliniensis* WT and *med3Δ/Δ* strains	NruI/SacII	ZL476/ZL477	KPP63/ZL289
EXpFA-P_{TDH3}*-TLOα12-3HA*$_{1X}$	Vector for overexpression of *TLOα12-3HA* in *C. dubliniensis* WT and *med3Δ/Δ* strains	NruI/SacII	ZL476/ZL477	KPP63/ZL289
EXpFA-P_{TDH3}*-TLO2-3HA*$_{1X}$	Vector for integrating 1 copy of P_{TDH3}-driven *TLO2-3HA* to *CdTLO1* locus with *SAT1* marker in *C. dubliniensis* WT and *med3Δ/Δ* strains	NruI/SacII	ZL476/ZL477	KPP63/ZL289

EXpFA-P$_{TDH3}$-TLO2-3HA$_{2X}$	Vector for integrating 2 copies of *P$_{TDH3}$*-driven *TLO2-3HA* to *CdTLO1* locus with *SAT1* marker in *C. dubliniensis* WT and *med3Δ/Δ* strains	NruI/SacII	ZL476/ZL477	ZL426/ZL289
EXpFA-P$_{TDH3}$-TLO2$_{1X}$	Intermediate construct			
EXpFA-P$_{TDH3}$-TLO2$_{2X}$	Vector for integrating 2 copies of *P$_{TDH3}$*-driven *TLO2* to *CdTLO1* locus with *SAT1* marker in *C. dubliniensis* WT and *med3Δ/Δ* strains	NruI/SacII	ZL476/ZL477	ZL426/ZL289
EXpFA-P$_{TDH3}$-HyNT1C-3HA$_{1X}$	Vector for overexpression of *HyNT1C-3HA* chimeric gene in *C. dubliniensis* WT and *med3Δ/Δ strains*	NruI/SacII	ZL476/ZL477	KPP63/ZL289
EXpFA-P$_{TDH3}$-T1NT2C-3HA	Vector for overexpression of *T1NT2C-3HA* in *C. dubliniensis* WT and *med3Δ/Δ strains*	NruI/SacII	ZL476/ZL477	KPP63/ZL289
EXpFA-P$_{TDH3}$-T2NT1C-3HA	Vector for overexpression of *T2NT1C-3HA* in *C. dubliniensis* WT and *med3Δ/Δ strains*	NruI/SacII	ZL476/ZL477	KPP63/ZL289
EXpFA-P$_{TDH3}$-HyNT2C-3HA	Vector for overexpression of *HyNT2C-3HA* in *C. dubliniensis* WT and *med3Δ/Δ strains*	NruI/SacII	ZL476/ZL477	KPP63/ZL289
EXpFA-P$_{TDH3}$-TLO1-GFP	Vector for expression of *TLO1-GFP* in *C. dubliniensis* WT and *med3Δ/Δ* strains	NruI/SacII	ZL476/ZL477	KPP63/ZL289
EXpFA-P$_{TDH3}$-TLOα12-GFP	Vector for expression of *TLOα12-GFP* in *C. dubliniensis* WT and *med3Δ/Δ* strains	NruI/SacII	ZL476/ZL477	KPP63/ZL289
EXpFA-P$_{TDH3}$-TLO2-GFP	Vector for expression of *TLO2-GFP* in *C. dubliniensis* WT and *med3Δ/Δ* strains	NruI/SacII	ZL476/ZL477	KPP63/ZL289
EXpFA-NLS- GFP-3HA	Vector for overexpression of nuclear-localized *GFP-3HA* in *C. dubliniensis*	NruI/SacII	ZL476/ZL477	KPP63/ZL289
EXpFA-NLS- GFP-TLO1-3HA	Vector for overexpression of nuclear-localized *GFP-TLO1-3HA* in *C. dubliniensis*	NruI/SacII	ZL476/ZL477	KPP63/ZL289

EXpFA-NLS-GFP-TLO1TAD-3HA	Vector for overexpression of nuclear-localized *GFP-TLO1TAD-3HA* in *C. dubliniensis*	NruI/SacII	ZL476/ZL477	KPP63/ZL289
EXpFA-NLS- GFP-TLO2-3HA	Vector for overexpression of nuclear-localized *GFP-TLO2-3HA* in *C. dubliniensis*	NruI/SacII	ZL476/ZL477	KPP63/ZL289
EXpFA- NLS-GFP-TLO2	Vector for overexpression of nuclear-localized *GFP-TLO2* in *C. dubliniensis*	NruI/SacII	ZL476/ZL477	KPP63/ZL289
EXpFA-NLS-GFP-TLO2TAD-3HA	Vector for overexpression of nuclear-localized *GFP-TLO2TAD-3HA* in *C. dubliniensis*	NruI/SacII	ZL476/ZL477	KPP63/ZL289
EXpFA-NLS-GFP-TLOα12-3HA	Vector for overexpression of nuclear-localized *GFP-TLOα12-3HA* in *C. dubliniensis*	NruI/SacII	ZL476/ZL477	KPP63/ZL289
EXpFA-NLS-GFP-TLOα12TAD-3HA	Vector for overexpression of nuclear-localized *GFP-TLOα12TAD-3HA* in *C. dubliniensis*	NruI/SacII	ZL476/ZL477	KPP63/ZL289
pACT1-TLOα12-GFP-SAT1	Vector for expression of *TLOα12-GFP* in *C. albicans* WT and mutant strains	StuI	ZL093/ZL174	KPP63/ZL094
pFA-P$_{TDH3}$- MED3-3HA	Intermediate construct			
pFA-P$_{TDH3}$- MED3-GFP	Intermediate construct			
pFA- gCdMED3-GFP	Vector for C-terminal GFP tagging *MED3* at its native locus in *C. dubliniensis*	HindIII/SacII	ZL398/ZL402	KPP63/ZL399
EXpFA-P$_{TDH3}$-GFP-TLO2-3HA	Vector for overexpression of *GFP-TLO2-3HA* from *TLO1* locus in *C. dubliniensis*	NruI/SacII	ZL476/ZL477	KPP63/ZL289
pMAL2-LexA-CdTLO2ΔN-URA3	Vector for integrating *MAL2* promoter driven *LexA(DBD)-CdTLO2ΔN* to *CaRPS10* locus	StuI	ZL093/ZL174	ZL173/ZL094

EXpFA-P$_{TDH3}$*-TLO2-6His3FLAG*$_{2X}$	Vector for integrating 2 copies of *P*$_{TDH3}$-driven *TLO2-6HIS3FLAG* to *CdTLO1* locus with *SAT1* marker in *C. dubliniensis* WT strain	NruI/SacII	ZL476/ZL477	ZL426/ZL289
pMAL2-LexA-CdTLO2-URA3	Vector for integrating *MAL2* promoter driven *LexA(DBD)-CdTLO2* to *CaRPS10* locus	StuI	ZL093/ZL174	ZL173/ZL094
pACT1- TLO2-3HA-SAT1	Intermediate construct			
EXpFA-P$_{ACT1}$*-TLO2-3HA*	*TLO2-3HA* over-expression vector driven by *CaACT1* promoter targeting *CdTLO1 locus*	NruI/SacII	ZL476/LM21	KPP63/ZL289

*: Plasmids are linearized by the listed restriction enzymes before used for transforming *C. albicans* or *C. dubliniensis* cells.
