# Supplementary material for: Amplification of TLO Mediator Subunit Genes Facilitate Filamentous Growth in Candida Spp
Source: PLoS Genet. 2016 Oct 14;12(10):e1006373. doi: 10.1371/journal.pgen.1006373 (PMC5065183; doi:10.1371/journal.pgen.1006373)
Supplement: S5 Table — (PDF) [file pgen.1006373.s031.pdf]

S5 Table. List of primers used in this study

Primer	Primer sequence (5'→3')
ZL282	GACATTAAGCTTTCTGACAACCG
ZL284	GGTACCCGGGGATCCGTCGACCATTACCAAATTGGTTCAAGAAGC
ZL286	tgGTAgtttaaacGTAGGGTAGTGGGTAAAAGCg
ZL287	ACGGAccgcggCTGGAGTTCGATTTTCGGCTAGG
ZL304	AAAAAGCTAGCGTCGACGGCGTGAAATGAGTTGTAAC
ZL305	tCTTGTTTGTAAGTTAGCTGACATTGTTAATTAATTTGATTTGTAAAAATTTGTTT GATG
ZL306	CCCTGAGGCCTTTAATATATCGTTGAGTGAGTTCTCTAGTCTTGTTTGTAAGTT AGC
ZL307	TGTTAATTAATTTGATTTGTAAAAATTTGTTTGATG
ZL308	CAAATTTTTACAAATCAAATTAATTAACAatgtctgataaaccaaaaagtg
ZL311	GGTACCCGGGGATCCGTCGACCATTACCAAACTGGTTTAAAAAGC
ZL302	TTCATACGCGTGGATCCTAAGTTAAATCTGGAACTGATTTTG
ZL303	TATCTGGCGCGCCAACTAGCCGGCaTACAAACTCTCGAGCTTTGAATAGATC ATTACATG
ZL304	AAAAAGCTAGCGTCGACGGCGTGAAATGAGTTGTAAC
ZL305	tCTTGTTTGTAAGTTAGCTGACATTGTTAATTAATTTGATTTGTAAAAATTTGTTT GATG
ZL306	CCCTGAGGCCTTTAATATATCGTTGAGTGAGTTCTCTAGTCTTGTTTGTAAGTT AGC
ZL307	TGTTAATTAATTTGATTTGTAAAAATTTGTTTGATG
ZL309	CAAATTTTTACAAATCAAATTAATTAACAATGCCAGAAAACCTCCAAAC
ZL311	GGTACCCGGGGATCCGTCGACCATTACCAAACTGGTTTAAAAAGC
ZL299	GAGCTGGCGCGCCACTACCCTACACATCAATTACCAAATTGGTTCAAGAAGC
ZL320	TACTGGCGCGCCCTAATTACCAAACTGGTTTAAAAAGC
ZL324	ctgtcaaagcttaaaATGTCAGCTAACTTACAAAC
ZL187	ctgtcaaagcttaaaATGCCAGAAAACCTCCAAAC
ZL325	TCAAACTGCAGCTCTAGTTTTGACGCTGG
ZL332	CGCTTCCATTTCTTCTTTCTTTCTTGCCGCTTCTTC
ZL333	AAAGAAGAAATGGAAGCG
ZL334	TTCGAGCTTTTGTTTGTTTTCAACCATAACACCGAG
ZL335	AACAAACAAAAGCTCGAA
ZL340	CATAACGGCGACACACCACTTTGCATCATTGAGTTTCGACAC

ZL341	AAGTGGTGTGTCGCCGTTATG
ZL342	GATTAGTTCATTGTTGGGGCCAGTAATCACATTGCTTTGC
ZL343	GGCCCCAACAATGAACTAATC
ZL322	TAAAGTCGTCAAAGTTGTCAAACTTTGGGGCCTGGTTCTC
ZL323	GACAACTTTGACGACTTTA
ZL326	GTTGTCAAACTTTTGTGGTTCTTCTTGCTTCTTTCTCAATGCTTCC
ZL327	CCACAAAAGTTTGACAAC
ZL328	GGCCTCTTCTTCACATTTCAACCGCCTAGCTTCTGC
ZL329	AAATGTGAAGAAGAGGCC
ZL330	caattCGTCATGTTTCTTTTTCTTCTCAATCATAACGGCGACACA
ZL331	AAGAAAAAGAAACATGACGAATTG
ZL336	CATAACACCGAGACACCACTCTGCATCATTAAATTTGGAAAC
ZL337	GAGTGGTGTCTCGGTGTTATG
ZL338	GATTAGTTCGTTGTTGGGGCTAGTTATCAAATTGCTTTGC
ZL339	AGCCCCAACAACGAACTAATC
ZL350	GATATATTAAAGGCCTCAGGGTACATATTAGAGGTAATCGACCAAAAC
ZL351	GCTAACTTACAAACAAGaTTACATAACTCACTCGACGAGATA
ZL321	cCAATGAATTCGTCAAAGTTGTCAAACTT
ZL458	CTTATAAGCTTTTACAACCACCGATTGCAATTC
ZL459	GTCAGAAAGCTTAATGTCGTTAC
ZL361	CCGGGGATCCGTCGACCATTACCAAACTGGTTTAAGAAACTATCtACATCAAA CTCTTCA
ZL362	CAAATTTTTACAAATCAAATTAATTAACAATGTCAGCCAATTTACAAATAAAAC
ZL444	AAGAAAATCATCAAAATTGTCAAACTTTGGGGCCTGGTTCTC
ZL445	GACAATTTTGATGATTTTCTTGG
ZL446	GTCGAATCCAATGAATTCATCAAAATTGTCAAACTTTTG
ZL447	GAATTCATTGGATTCGAC
ZL401	TCGACGGATCCCCGGTGGTGGTTCTAAAGGTGAAGAATTA
ZL402	GAACTGGCGCGCCTTATTTGTACA ATTCATCCATACC
ZL068	cgacagcgccagtctcgac
ZL497	CGCTGGCGCGCCTCAATTACCAAACTGGTTTAAGAA
ZL505	CAAACAAATTTTTACAAATCAAATTAATTAACAATGGGTGCTCCTCCAAAAAAG AAGAGAAAGGTAggtactggtggtggttctaaag
ZL506	CCCGGGGATCCgaacgcgtgGtttgtacaattcatccatac
ZL511	ggtccacgcgttGGTGGAGGTCCAGGTGGAATGTCAGCTAACTTACAAACa

ZL227	CGGGacgcgtTAAGTTTGACAATTTTGATG
ZL503	ggtccacgcgttGGTGGAGGTCCAGGTGGAATGTCAGCCAATTTACAAATA
ZL504	ggtccacgcgttGGTGGAGGTCCAGGTGGAAAGTTTGACAATTTTGATG
ZL168	ggtccacgcgttGGTGGAGGTCCAGGTGGAATGCCAGAAAACCTCCAAAC
ZL169	ggtccacgcgttGGTGGAGGTCCAGGTGGATTTGACAACTTTGACG
ZL308	CAAATTTTTACAAATCAAATTAATTAACAatgtctgataaaccaaaaagtg
ZL310	GGTACCCGGGGATCCGTCGACCaataacaattggttgattagc
ZL396	ATTGGCGGCCGCAGAGCCAAGGTGACACTGTAC
ZL397	ATTCTCCGCGGTGCCAACAAATAGGAGAGACGCC
ZL510	CAAACAAATTTTTACAAATCAAATTAATTAACAATGGCTggttctaaaggtgaagaatta
ZL426	ACGCCTTACAAACTCAAA
ZL427	TCAAGCAAGTTCATATTCAAG
ZL495	CGGGAATATCAATTCCAAGAAAATCGTCAAAGTTGTCAAACTT
ZL496	cTTGGAATTGATATTCCCGatg
ZL313	AATGAAGAGTTTGATGTAGA
ZL201	TAGTCAGGAACATCGTATG
ZL288	TATAGTTGAATTCTGATTTATGG
ZL289	GCACTGGGCATCTCAAATTGCG
KPP63	CACAGGATGACGCCTAAC
ZL093	CTCACGCACGCCCATACTAC
ZL094	GAGAGAAACTATATTATACAC
ZL174	GGAATGCTTATTTGAAAAAGACTGGC
ZL476	CTCATTCGTCTGCCCCACTTTTC
ZL477	GGAGAACACTATATCCCGTCAATTG
ZL173	Gatgaagcaactgtcaaacgc
LM21	CTAATTAACGTGTGTGTATGGATC
ZL398	ATCAACGGGAATTACTACCATTGG
ZL399	CTATGGTAACTTTGTTTGATCTCCG
ZL422	GGGGACAATAGCAACAACGACAATCAAGTCAATGAAGAGTTTGATGTAGATA GCTTCTTGAACCAATTTGGTAATggtcgacggatcccc
ZL423	ATATAATGTTGCTTATACTTGAAATACAAATGGTTGATGGTAAAGAAACGCTTT TACCCACTACCCTACACAtcgatgaattcgagctcg
ZL512	TTGCTTGATGACGCCAACACCTTCAACGATAATGAAGAGTTTGATGTGGATAG TTTCTTAAACCAGTTTGGTAATggtcgacggatcccc
ZL513	AAAAACTACTTGTGCGAGGTTTTCTGTAATATTATTGGTTCTTCCTATTTGAGC TAGACACACTACCCTAtcgatgaattcgagctcg

KPP035	GGGTTGAACATGACATTGCTCGACAATGGCGACCACGTAAACGAAGAGTTTG<br>ATGTAGACAGCTTTTTAAACCAGTTTGGTAATGGTCGACGGATCCCCG
KPP037	CTTTGGGTCTTTAGTTCATCCGTTAGTTCATTTAAGGTTATAGGTCTTCCATAT<br>GGAAACAAAAAGGTCAAATGATCGATGAATTCGAGCTCGTT
KPP041	TGCTTTTGACACGTATTTTATATTTGATCGTGGCTGATTGTGATTGTTAGGGTG<br>ATTCTGAGATAACCATTCAGTCGATGAATTCGAGCTCGTT
AZq026	TGGTGATGGTGTTACTCACG
AZq027	GACAATTTCTCTTTCAGCAC
ZL190	ACAACTAAGCGACCACATTC
ZL191	CAATCATAACGGCGACACA
ZL315	CAAGAAAGCAGAGGAAAT
ZL316	TGATGTCAAAGCCAATAAAG
ZL318	ACTTTCAATTCGTCATGTTTC
ZL386	CGGTAAGGTTACTGGTAAG
ZL387	AATGGCAATCTCAATGGT
ZL724	CCATAGCCAATAACCTCAA
ZL725	GACGACACATCCATAAGAA
ZL728	GACTCTTAGAATTGATGATG
ZL729	TTGATTATTGGACGGATAT
ZL730	CCGAATAGTCTTGTGTATC
ZL731	TCATTACTGGCAATAGGA
ZL809	TTCTGTTCCATTCCATTCG
ZL810	TCTTGAGGCTTGACTGAT
ZL813	TTCTATATTGACGACCTACCT
ZL814	TACAGTTGCCACCTACAT
ZL815	GCAAGTCGGTCAATTCTAA
ZL816	GCCAACTCATAATCCTCAG
ZL817	GCTATAACCTCAGACACTATT
ZL818	CATTCGCTTCATCAACAAT
ZL819	GATGATGACTCTGATGAAG
ZL820	TAGATCGTTCCATGTTGA
ZL424	TGCAGGGTTCATGTTTGAG
ZL425	TTTGAATGTTGTCGTTTAGTTGT
ZL456	ACTGAATCTGCTGTGTCT

ZL457	CTCGGAATCGTTGTATAATGT
