# Supplementary material for: Amplification of TLO Mediator Subunit Genes Facilitate Filamentous Growth in Candida Spp
Source: PLoS Genet. 2016 Oct 14;12(10):e1006373. doi: 10.1371/journal.pgen.1006373 (PMC5065183; doi:10.1371/journal.pgen.1006373)
Supplement: S6 Table — (PDF) [file pgen.1006373.s032.pdf]

S6 Table. Construction details for Series A and Series B Chimeric Genes

Chimeric Gene Name	Primer Pair X	Template for Primer Pair X	Primer Pair Y	Template or Primer Pair Y	Resulting *Ca* Over-expression Construct
12N-1	ZL187/ ZL332	*TLOα12-3HA**	ZL333/ ZL325	*TLO1-3HA***	*pACT1-(12N-1)-3HA-SAT1*
12N-2	ZL187/ ZL334	*TLOα12-3HA*	ZL335/ ZL325	*TLO1-3HA*	*pACT1-(12N-2)-3HA-SAT1*
12N-3	ZL187/ ZL340	*TLOα12-3HA*	ZL341/ ZL325	*TLO1-3HA*	*pACT1-(12N-3)-3HA-SAT1*
12N-4	ZL187/ ZL342	*TLOα12-3HA*	ZL343/ ZL325	*TLO1-3HA*	*pACT1-(12N-4)-3HA-SAT1*
TN-1	ZL324/ ZL326	*TLO1-3HA*	ZL327/ ZL325	*TLOα12-3HA*	*pACT1-(TN-1)-3HA-SAT1*
TN-2	ZL324/ ZL328	*TLO1-3HA*	ZL329/ ZL325	*TLOα12-3HA*	*pACT1-(TN-2)-3HA-SAT1*
TN-3	ZL324/ ZL330	*TLO1-3HA*	ZL331/ ZL325	*TLOα12-3HA*	*pACT1-(TN-3)-3HA-SAT1*
TN-4	ZL324/ ZL336	*TLO1-3HA*	ZL337/ ZL325	*TLOα12-3HA*	*pACT1-(TN-4)-3HA-SAT1*
TN-5	ZL324/ ZL338	*TLO1-3HA*	ZL339/ ZL325	*TLOα12-3HA*	*pACT1-(TN-5)-3HA-SAT1*

* ‘TLOα12-3HA’ = $pFA\text{-}P_{TDH3}\text{-}TLO\alpha12\text{-}3HA_{1X}$
** ‘TLO1-3HA’ = $pFA\text{-}P_{TDH3}\text{-}TLO1\text{-}3HA_{1X}$
