# Supplementary material for: Amplification of TLO Mediator Subunit Genes Facilitate Filamentous Growth in Candida Spp
Source: PLoS Genet. 2016 Oct 14;12(10):e1006373. doi: 10.1371/journal.pgen.1006373 (PMC5065183; doi:10.1371/journal.pgen.1006373)
Supplement: S7 Table — (PDF) [file pgen.1006373.s033.pdf]

S7 Table. Construction details for ‘non-series A/B’ chimeric genes.

Chimeric Gene Name	Primer Pair X	Template for Primer Pair X	Primer Pair Y	Template or Primer Pair Y	Resulting *Ca* Overexpression Construct
$T12H_2$	ZL282/ ZL338	*(TN-4)-3HA**	ZL339/ ZL325	*(12N-3)-3HA*	*pFA-P_{TDH3}-$T12H_2$-3HA*
$12TH_2$	ZL282/ ZL342	*(12N-3)-3HA*	ZL343/ ZL325	*(TN-4)-3HA*	*pFA-P_{TDH3}-$12TH_2$-3HA*
HyNT1C	ZL282/ ZL336	*$12TH_2$-3HA*	ZL327/ ZL325	*12NT1C-3HA*	*pFA-P_{TDH3}-HyNT1C-3HA*
HyNΔC	ZL282/ ZL336	*HyNT1C-3HA*	ZL337/ ZL325	*TLOα12ΔC-3HA***	*pFA-P_{TDH3}-HyNΔC-3HA*
T1NT2C	ZL282/ ZL444	*TLO1-3HA*	ZL445/ ZL325	*TLO2-3HA*	*pFA-P_{TDH3}-T1NT2C-3HA*
T2NT1C	ZL282/ ZL446	*TLO2-3HA*	ZL447/ ZL325	*TLO1-3HA*	*pFA-P_{TDH3}-T2NT1C-3HA*
HyNT2C	ZL282/ ZL495	*12TH2*	ZL496/ ZL352	*TLO2-3HA*	*pFA-P_{TDH3}-HyNT2C-3HA*

* ‘*(TN-4)-3HA*’ stands for the *pFA-P_{TDH3}-(TN-4)-3HA* plasmid throughout this table.

**‘*TLOα12ΔC-3HA*’ stands for the *pFA-P_{TDH3}-TLOα12ΔC-3HA* plasmid. It differs from *pFA-P_{TDH3}-TLOα12-$3HA_{1X}$* in that a C’ terminal truncated version of *TLOα12,* which only encodes the first 166 amino acid residues, was *3HA*-tagged and driven by a *TDH3* promoter.
